# Environmental and Socio–Cultural Factors Impacting the Unique Gene Pool Pattern of Mae Hong-Son Chicken

**DOI:** 10.3390/ani13121949

**Published:** 2023-06-10

**Authors:** Wongsathit Wongloet, Worapong Singchat, Aingorn Chaiyes, Hina Ali, Surachai Piangporntip, Nattakan Ariyaraphong, Trifan Budi, Worawit Thienpreecha, Wannapa Wannakan, Autchariyapron Mungmee, Kittipong Jaisamut, Thanyapat Thong, Thitipong Panthum, Syed Farhan Ahmad, Artem Lisachov, Warong Suksavate, Narongrit Muangmai, Rattanaphon Chuenka, Mitsuo Nunome, Wiyada Chamchumroon, Kyudong Han, Aniroot Nuangmek, Yoichi Matsuda, Prateep Duengkae, Kornsorn Srikulnath

**Affiliations:** 1 Animal Genomics and Bioresource Research Unit (AGB Research Unit), Faculty of Science, Kasetsart University, 50 Ngamwongwan, Chatuchak, Bangkok 10900, Thailand; wongsathit.w@ku.th (W.W.); fsciwos@ku.ac.th (W.S.); hinaali.ibs@suit.edu.pk (H.A.); chai.ppt21@gmail.com (S.P.); nattakan.ari@ku.th (N.A.); trifanbudi26@gmail.com (T.B.); worawit.t@ku.th (W.T.); wannapa.wannakan12327@gmail.com (W.W.); autchariyapron.tam@gmail.com (A.M.); kjaisamut10@gmail.com (K.J.); thongthanyapat@gmail.com (T.T.); thitipong.pa@ku.th (T.P.); syedfarhan.a@ku.th (S.F.A.); aplisachev@gmail.com (A.L.); fforwos@ku.ac.th (W.S.); ffisnrm@ku.ac.th (N.M.); kyudong.han@gmail.com (K.H.); yoimatsu@nuagr1.agr.nagoya-u.ac.jp (Y.M.); prateep.du@ku.ac.th (P.D.); 2 Special Research Unit for Wildlife Genomics (SRUWG), Department of Forest Biology, Faculty of Forestry, Kasetsart University, 50 Ngamwongwan, Chatuchak, Bangkok 10900, Thailand; 3 School of Agriculture and Cooperatives, Sukhothai Thammathirat Open University, Nonthaburi 11120, Thailand; chaiyes.stou@gmail.com; 4 School of Integrated Science, Kasetsart University, 50 Ngamwongwan, Chatuchak, Bangkok 10900, Thailand; 5 Bureau of Conservation and Research, Zoological Park Organization of Thailand, Bangkok 10300, Thailand; 6 Laboratory of Animal Cytogenetics and Comparative Genomics (ACCG), Department of Genetics, Faculty of Science, Kasetsart University, 50 Ngamwongwan, Chatuchak, Bangkok 10900, Thailand; 7 Department of Fishery Biology, Faculty of Fisheries, Kasetsart University, Bangkok 10900, Thailand; 8 Faculty of Humanities, Kasetsart University, Bangkok 10900, Thailand; rattanaphon.c@ku.th; 9 Department of Zoology, Faculty of Science, Okayama University of Science, Ridai-cho 1-1, Kita-ku, Okayama 700-0005, Japan; mtnunome@ous.ac.jp; 10 Department of National Park, Wildlife and Plant Conservation, Ministry of Natural Resources and Environment, Bangkok 10900, Thailand; wbd1680@gmail.com; 11 Department of Microbiology, Dankook University, Cheonan 31116, Republic of Korea; 12 Bio-Medical Engineering Core Facility Research Center, Dankook University, Cheonan 31116, Republic of Korea; 13 Mae Hong Son Provincial Livestock Office, Department of Livestock Development, Ministry of Agriculture and Cooperatives, Mae Hong Son 58000, Thailand; anirootm@yahoo.com; 14 Amphibian Research Center, Hiroshima University, 1-3-1, Kagamiyama, Higashihiroshima 739-8526, Japan

**Keywords:** gene pool, selective sweep, Mae Hong Son chicken, habitat suitability, food security

## Abstract

**Simple Summary:**

Mae Hong Son chicken is a local breed of North Thai domestic chicken widely distributed in Mae Hong Son; however, its genetic characterization, origin, and diversity remain unclear. Here, we studied the socio–cultural, environmental, and genetic aspects of the Mae Hong Son chicken breed, and we investigated its genetic diversity and footprint using the genotyping of 28 microsatellite markers and analyzed mitochondrial D-loop sequencing data. Genetic diversity analysis indicated that the Mae Hong Son chicken population is genetically highly diverse, with 35 haplotypes clustered into haplogroups A, B, E, and F, mostly in the North ecotype. Allelic gene pool patterns showed a unique DNA fingerprint of the Mae Hong Son chicken, as compared to other breeds and red junglefowl. A genetic introgression of some parts of the gene pool of red junglefowl and other indigenous breeds was identified in the Mae Hong Son chicken, supporting the hypothesis of the origin of the Mae Hong Son chicken as a crossbreed between red junglefowl and Thai indigenous village chickens that adapted to the environmental, social, and cultural conditions in its habitat. These findings enrich our understanding of the genetic blueprint, origin, and evolutionary process of the Mae Hong Son chickens and lay the foundation for future studies to improve domestic chickens using this indigenous chicken breed.

**Abstract:**

Understanding the genetic diversity of domestic chicken breeds under the impact of socio–cultural and ecological dynamics is vital for the conservation of natural resources. Mae Hong Son chicken is a local breed of North Thai domestic chicken widely distributed in Mae Hong Son Province, Thailand; however, its genetic characterization, origin, and diversity remain poorly understood. Here, we studied the socio–cultural, environmental, and genetic aspects of the Mae Hong Son chicken breed and investigated its diversity and allelic gene pool. We genotyped 28 microsatellite markers and analyzed mitochondrial D-loop sequencing data to evaluate genetic diversity and assessed spatial habitat suitability using maximum entropy modeling. Sequence diversity analysis revealed a total of 188 genotyped alleles, with overall nucleotide diversity of 0.014 ± 0.007, indicating that the Mae Hong Son chicken population is genetically highly diverse, with 35 (M1–M35) haplotypes clustered into haplogroups A, B, E, and F, mostly in the North ecotype. Allelic gene pool patterns showed a unique DNA fingerprint of the Mae Hong Son chicken, as compared to other breeds and red junglefowl. A genetic introgression of some parts of the gene pool of red junglefowl and other indigenous breeds was identified in the Mae Hong Son chicken, supporting the hypothesis of the origin of the Mae Hong Son chicken. During domestication in the past 200–300 years after the crossing of indigenous chickens and red junglefowl, the Mae Hong Son chicken has adapted to the highland environment and played a significant socio–cultural role in the Northern Thai community. The unique genetic fingerprint of the Mae Hong Son chicken, retaining a high level of genetic variability that includes a dynamic demographic and domestication history, as well as a range of ecological factors, might reshape the adaptation of this breed under selective pressure.

## 1. Introduction

Poultry farming is important in the agricultural industry, accounting for 33% of global agricultural production [1]. Chickens are postulated to have been domesticated from the red junglefowl [2], native across Southeast Asia to Southwest China, approximately 10,000 years ago [3]. Domestic chicken (*Gallus gallus domesticus*) has been one of the most ubiquitous globally domesticated animal bioresources throughout human civilization, providing food security as a high source of protein. Domestic chickens are additionally used as companion animals, with socio–cultural roles as ornamental, long-crowing, and game-fighting birds [4,5,6,7,8,9]. Intensive human-directed selection for economic traits has led to the development of breeds from a large diversity of domestic chickens and red junglefowl.

**Figure 1:**
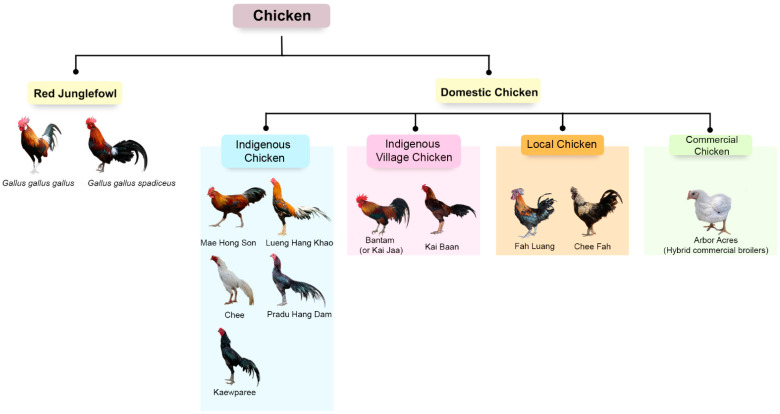
Schematic representation of red junglefowl and domestic chickens, including local, indigenous village, commercial chicken, and indigenous chickens. Thai indigenous chickens such as Mae Hong Son, Keawparee, Pradu Hang Dam, Lueng Hang Khao, or Chee, displaying special visual characteristics such as comb type, skin color, and feather color may have promoted preferential selection by smallholder farmers, thereby increasing frequencies of desirable phenotypes [17]. In contrast, Thai indigenous village chickens such as Kai Baan and bantam (or Kai Jaa) are maintained under relaxed human selection in local communities for food and are distinct from commercial breeds reared in industrialized poultry farms. Local breeds such as Chee Fah and Fah Luang are possibly derived from indigenous chickens hailing from other geographic regions that adapted to the prevailing environmental conditions [18].

In Thailand, domestic chickens are tentatively classified into four major groups: indigenous, indigenous village, commercial, and local chickens (Figure 1) [10]. These groups play pivotal roles in the food supply chain and adapt to local environmental conditions under smallholder farmers or the local community [11]. Thai domestic chickens have acquired diverse genetic characteristics and adapted to various challenging conditions in disparate locations to overcome heat stress, humidity, disease, and various agroecosystems, with stringent human selection for production and/or aesthetic values, resulting in the predicted genetic selective sweep for some breeds [12,13,14,15,16]. The domestication process has shaped the genomic landscape of domestic chickens, resulting in a wide spectrum of breeds and ecotypes. Evaluations of the diversity and structure of various domestic breeds are important to identify valuable genetic resources and better understand the domestication process from red junglefowl [17]. Recent research on the genetic diversity of red junglefowl and Thai indigenous chickens revealed a large and diverse genetic origin or gene pool of the red junglefowl that has many geographically different populations (ecotypes) [15,16]. Surprisingly, based on microsatellite genotyping, the present gene pool of most red junglefowl populations may be different from those of the ancestral populations of Thai indigenous chickens [15,16]. This finding leads us to believe that highly adaptive processes such as genetic sweep with selection and mutation could have occurred in Thai indigenous chickens during the domestication process. To further elucidate the evolutionary process of Thai chicken domestication, the genetic footprint of a larger number of domestic chicken breeds of different origins needs to be investigated.

Mae Hong Son chicken, of Mae Hong Son Province, Thailand (19°18′2.40″ N, 97°58′7.19″ E), an area mostly consisting of highlands as complex mountain ranges with rainforests, is a classical chicken breed of North Thailand [19]. The Mae Hong Son chickens have a crest on top of their heads, white hair at the base of their tails, and a body covered in black feathers with dark yellow stripes on their neck and tail. A Mae Hong Son chicken produces 40 to 123 eggs per year, and its body weight at 20 weeks ranges from 842 to 1130 g [20]. While the Mae Hong Son chicken has lower productivity than commercial chicken breeds, its production is not significantly different from other Thai local breeds [21]. However, the Mae Hong Son chicken breed is raised in rural areas, mostly in highland villages of the Karen, Lahu, Pgakenyor, Hmong, and Tai Yai communities. The chickens are bred for consumption, regional marketing, and recreational purposes (Supplementary Data S1). The local people believe that the Mae Hong Son chicken originated from a crossbreed between the red junglefowl of Northern Thailand and Thai indigenous village chicken [22]. The Mae Hong Son chicken should, therefore, possess some portions of the gene pool (genetic footprint) of the Northern ecotype of red junglefowl. The Mae Hong Son chicken might additionally have undergone a genetic selective sweep. This study aimed to analyze the gene pool of three populations of Mae Hong Son chicken, using 28 microsatellite markers and mitochondrial (mt) D-loop sequences to investigate the genetic diversity. The findings were then compared to a broad gene pool library, available under the “Siam Chicken Bioresource project” [15,16]. The maximum entropy modeling (MaxEnt) [23] was used, based on spatial suitability analysis of Mae Hong Son chicken to precisely assess land suitability for chicken production. This study evaluated genetic footprints of evolutionary history to identify breeds/ecotypes of the Mae Hong Son chicken that originated from a cross between the red junglefowl and Thai domestic chicken and later evolved to suit various local environments.

## 2. Materials and Methods

### 2.1. Collection of Social and Cultural Diversity Parameters of Mae Hong Son Chicken

Cultural diversity was investigated by a literature search from the Northern Thailand Cultural Encyclopedia [24], Aphiwan Phansuk’s Chicken and Lanna Folklife [25], and Pollavat Prapattong’s research project Fighting Cocks as Intangible Cultural Heritage of Upper-Northern Thailand [26]. Other local information was additionally gleaned from the written literature within the Lanna community and the databases of the Institute of Tai Yai Studies, Mae Hong Son Community College [24,25,26].

### 2.2. Analysis of Study Area for Habitat Suitability of Mae Hong Son Chicken

**Figure 2:**
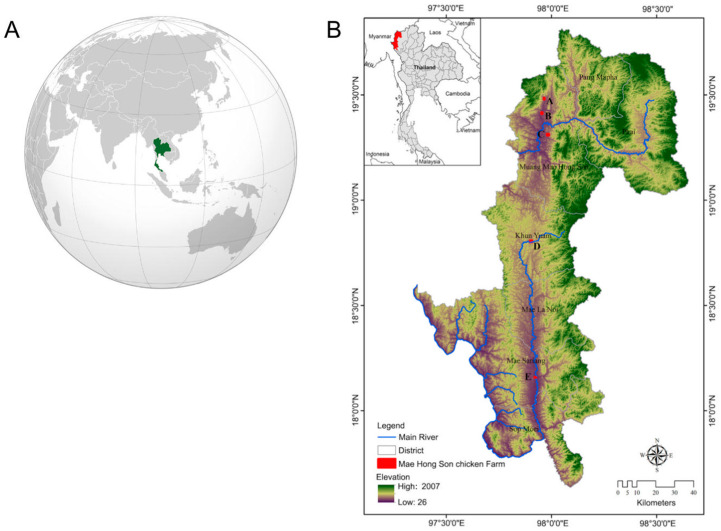
The location of Thailand on the world map (**A**). Illustration of the geography of Thailand (**B**), with a focus on the Mae Hong Son province, including topographical features and Mae Hong Son chicken farms.

**Figure 3:**
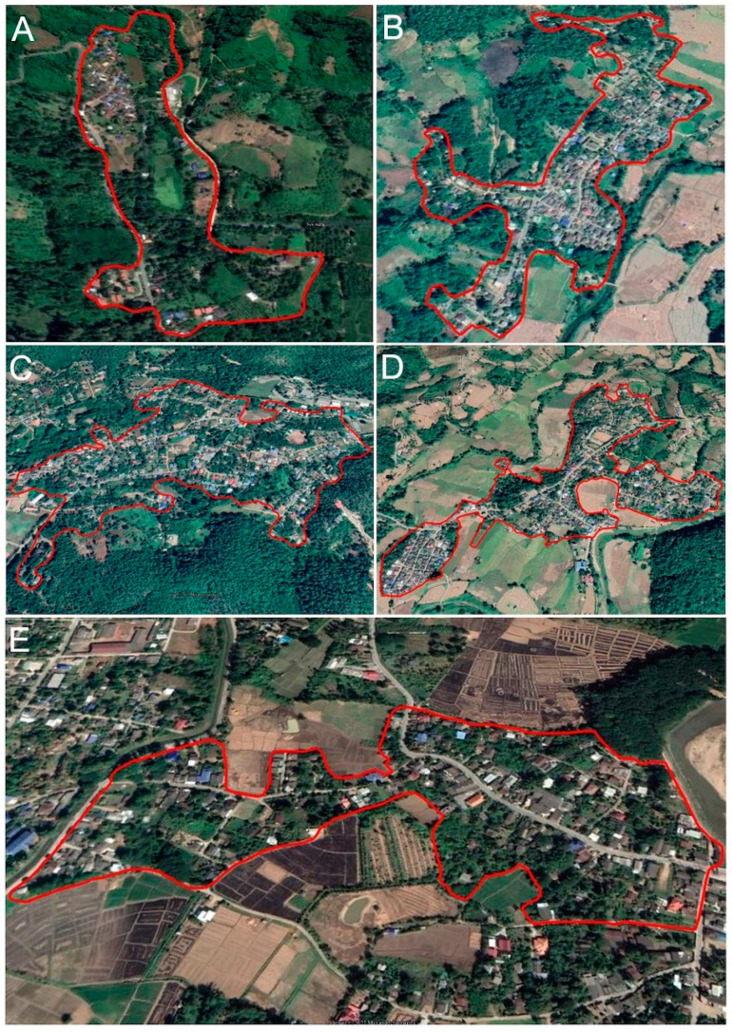
Study area of Mae Hong Son chickens. (**A**) Ban Thop Sok, Mueang Mae Hong Son District, Mae Hong Son; (**B**) Ban Mae Sanga, Mueang Mae Hong Son District; (**C**) Ban Chanmuang, Mueang Mae Hong Son District, Mae Hong Son; (**D**) Ban Luang, Khun Yuam District, Mae Hong Son; and (**E**) Ban Klong, Mae Sariang District, Mae Hong Son.

The Mae Hong Son Province in Northern Thailand (97°20′–98°39′ E, 17°38′–19°48′ N) has seven districts, namely Mueang Mae Hong Son, Khun Yuam, Pai, Mae Sariang, Mae La Noi, Sop Moei, and Pang Mapha (Figure 2 and Figure 3). The Mae Hong Son Province covers an area of 12,681 km^2^, with a landscape characterized by mountainous topography and an elevation ranging from 26 to 2005 m.

### 2.3. Occurrence Data of Mae Hong Son Chicken Collection

Data on the occurrence of the Mae Hong Son chicken were collected from smallholder poultry and backyard chicken farms. The data were analyzed to construct a potential suitability model for the Mae Hong Son chicken. Sampling occurrence data covered five villages in four subdistricts of three districts in the study area where the purpose of the Mae Hong Son chicken was focused on poultry production rather than socio–cultural roles by the local communities (Figure S1A–D). We created fishnet polygons of 30 m^2^ within the boundaries of 5 villages for predictive modeling of the Mae Hong Son chicken and obtained a random sample point of 30% from the total polygons using ArcGIS 10.4.1 [27].

### 2.4. Environmental Data Collection

We aimed to investigate the potential impact of various environmental variables on the Mae Hong Son chicken. The environmental variables under consideration were elevation, distance to water, the normalized difference vegetation index (NDVI), tree canopy cover, and forest canopy height. To gather the required elevation data, we accessed the Department of National Parks, Wildlife and Plant Conservation of Thailand, Ministry of Natural Resources and Environment’s database. The elevation data were obtained at a scale of 30 m-resolution (Figure S1A). Distance to water was calculated as the Euclidean distance to main rivers (Figure S1B). Data for inland water layers were obtained from the Land Development Department, Ministry of Agriculture and Cooperatives, Thailand. The NDVI-estimated vegetation activity, measured by Landsat 8 satellite images for the period of January 2021 to July 2022, was provided by the U.S. Geological Survey from the Earth Explorer website (Figure S1C). Global Landsat analysis-ready data were used to extrapolate the Global Ecosystem Dynamics Investigation footprint-level forest canopy height measurements [28] (Figure S1D), and ArcGIS was used to interpolate environmental factors at the same spatial resolution of approximately 30 m into a raster format.

### 2.5. Species Distribution Modeling

The MaxEnt algorithm was used with the available software package MaxEnt ver. 3.4.4 to perform species distribution modeling of the Mae Hong Son chicken [23,29]. MaxEnt combines the environmental predictors and location data as input [30] and uses the location-only data as appropriate for species with small locations [31,32,33] (Supplementary Data S1).

### 2.6. Specimen Collection and DNA Extraction

Fifty Mae Hong Son chickens were sampled at the Mae Hong Son Livestock Research and Breeding Center, Mae Hong Son, Thailand and the military base in Ban Klang, Chiang Mai, Thailand, as three populations derived from the farmer community, including Mae Hong Son, Thailand (MHSF) (*n* = 10), Mae Hong Son Livestock Research and Breeding Center (MLRBC) (*n* = 30), and Chiang Mai Livestock Research and Breeding Center, Chiang Mai, Thailand (CLRBC) (*n* = 10). Detailed information regarding the individuals sampled are presented in Table S1 and Figure S2. The blood samples obtained from the live chickens were carefully collected using Vacuette ^®^ 21-gauge needles and then transferred into vials that contained 5 mM EDTA (Greiner Bio-One, Kremsmünster, Austria). These vials were then stored at 4 °C, until the subsequent analysis required their use. The extraction of total gDNA from the blood samples was conducted, following the methodology established by Supikamolseni et al. [34]. The quality and quantity of the DNA were evaluated, using both 1% agarose gel electrophoresis and a NanoDrop ™ 2000 Spectrophotometer (Thermo Fisher Scientific, Wilmington, DE, USA). The Animal Experiment Committee of Kasetsart University approved all the animal care and experimental procedures used in this study (approval number: ACKU65-SCI-021). We ensured that the welfare and ethical treatment of the Mae Hong Son chickens were prioritized during the collection of blood samples and DNA extraction procedures. The study was conducted in compliance with the Regulations on Animal Experiments at Kasetsart University.

### 2.7. Mitochondrial D-Loop Sequencing and Data Analysis

Partial fragments of the Mitochondrial D-loop (mt D-loop) sequence were amplified by PCR using the following primers: Gg_D-loop_1F (5′-AGGACTACGGCTTGAAAAGC-3′) and Gg_D-loop_4R (5′-CGCAACGCAGGTGTAGTC-3′) [15,16,35,36]. Mt D-loop PCR amplification was conducted, as previously described by Singchat et al. [16]. The PCR reactions were performed under the following conditions: An initial denaturation was conducted at 94 °C for 4 min, followed by 40 cycles consisting of 30 s at 94 °C, 45 s at 58 °C, and 30 s at 72 °C. The reaction was completed with a final extension step at 72 °C for 10 min. The PCR products were visualized on a 1% agarose gel and cleaned up using the GenUP™ PCR Cleanup Kit (Biotechrabbit, Hennigsdorf, Germany), according to the manufacturer’s instructions. Fragment DNA sequences were determined using an ABI 3730XL automatic sequencer (Applied Biosystems, Foster City, CA, USA) at the DNA sequencing service of First Base Laboratories Sdn Bhd (Seri Kembangan, Selangor, Malaysia). Fragment DNA sequence identity was confirmed using the BLASTn and BLASTx programs (http://blast.ncbi.nlm.nih.gov/Blast.cgi, accessed on 10 October 2022) in the National Center for the Biotechnology Information (NCBI) database. Mt D-loop sequences from this study were submitted to the DNA Data Bank of Japan (DDBJ) (https://www.ddbj.nig.ac.jp/, accessed on 10 October 2022) (accession number: LC731861–LC731880 and LC731886–LC731915) (Table S1). Genetic diversity and population structure analyses based on mt D-loop sequences were conducted, as described in previous studies [15,16,37,38] (Supplementary Data S1).

To determine the phylogenetic positions and haplogroups of the Mae Hong Son chicken, multiple sequence alignments were performed using 496 (50 + 446) sequences in the mt D-loop dataset, including 446 chicken sequences (125 domestic chicken breeds and 321 red junglefowl) retrieved from the reference dataset of the Siam Chicken Bioresource Project [15,16]. Sequence alignment and the related sequence preparation were performed, as mentioned above. MrBayes version 3.2.6 was utilized to construct phylogenetic relationships, based on the Bayesian inference method [39]. The most appropriate DNA substitution model was identified using Kakusan4 [40]. To perform the Markov chain Monte Carlo (MCMC) analysis, 4 independent runs were executed for 1 million generations, with samples taken every 100 generations to produce a total of 10,000 trees. A majority-rule consensus tree was then generated, with mean branch lengths, and all initial sample points were eliminated during the burn-in period. The Bayesian posterior probability of the sampled tree population was expressed as a percentage.

### 2.8. Genotyping of Microsatellite Markers and Microsatellite Data Analysis

From the 30 markers suggested by the Food and Agriculture Organization [41] for investigating chicken biodiversity, a total of 28 microsatellite primer sets were chosen and listed in Table S2. Each forward primer was tagged at the 5′ end with fluorescent dye (6-FAM or HEX; Macrogen Inc., Seoul, Republic of Korea). The microsatellite PCR amplifications were conducted, based on previous studies [15,16,38]. To account for false allele amplification, each sample was run as a minimum of triplicates. The PCR reaction was performed with an initial denaturation at 95 °C for 10 min, followed by 35 cycles of 95 °C for 30 s, 55–58 °C for 30 s, and 72 °C for 30 s, with a final extension at 72 °C for 5 min (Table S2). The PCR products were checked using electrophoresis in a 1% agarose gel. Fragment length analysis based on fluorescently labeled DNA was run on an ABI 3730XL automatic sequencer (Applied Biosystems, Foster City, CA, USA) at the DNA sequencing service of Macrogen Inc. Peak Scanner version 1.0 software (Applied Biosystems) was utilized to determine the allelic size of each sample. Microsatellite genotyping data have been deposited in the Dryad Digital Repository Dataset (https://datadryad.org/stash/share/x2qlPmboMgCROXO8kQjFc5wF6INadoYmciW1y3WRJ88, accessed on 17 October 2022). Genetic diversity and population structure analyses based on microsatellite data of the Mae Hong Son chicken populations were conducted, as described in previous studies [15,16,37,38] (Supplementary Data S1).

### 2.9. Investigation of Genetic Origin of Mae Hong Son Chicken

The genetic lineage of the Mae Hong Son chicken was established by analyzing the microsatellite genotyping data of red junglefowl and Thai domestic chicken breeds/ecotypes, available at the “Siam Chicken Bioresource project” [15,16,38], which could be accessed from the Dryad Digital Repository Dataset (https://datadryad.org/stash/share/x2qlPmboMgCROXO8kQjFc5wF6INadoYmciW1y3WRJ88, accessed on 17 October 2022). We treated all three populations of the Mae Hong Son chicken as the same group and compared the results with other breeds/ecotypes. Pairwise genetic distances amongst breeds/ecotypes and clustering analysis with Principal Component Analysis (PCoA), Discriminant Analysis of Principal Components (DAPC), and STRUCTURE were performed as mentioned in Supplementary Data S1.

### 2.10. Detection of Genetic Selective Sweep in Mae Hong Son Chicken, Red Junglefowl, and Other Thai Domestic Chicken Breeds

To detect selection signals, phasing was performed using genotype data for each population. Molecular genetic diversity was estimated using the expected heterozygosity (*H*_e_), inbreeding coefficient (*F*_IS_), and observed heterozygosity (*H*_o_) of each individual/population. These statistics were plotted for each microsatellite locus (total of 28 loci) to predict the signature of a selective sweep. A pattern of higher *F*_IS_ coupled with low *H*_e_ was considered a signature of a selective sweep or purifying selection, whereas a low *F*_IS_ coupled with high *H*_e_ indicated the tendency of neutral or balancing selection [42].

## 3. Results

### 3.1. Land Suitability of Mae Hong Son Chicken

The Mae Hong Son Province is spread over an area of 12,621.68 km^2^, and the land that is potentially unsuitable (*p* < 0.2) for the inhabitation of chickens was estimated as 12,227.53 km^2^ (96.88% of the total area). An area of 0.03 km^2^ (0.0003% of the total area) was predicted to have a very high suitability (*p* > 0.8), with high suitability (0.6 < *p* ≤ 0.8) at 24.23 km^2^ (0.19% of the total area), moderate suitability (0.4 < *p* ≤ 0.6) at 138.45 km^2^ (1.10% of the total area), and least suitability (0.2 ≤ *p* ≤ 0.4) at 231.44 km^2^ (1.83% of total area) [43] (Figure S3). The marginal response curves illustrate the influence of elevation, forest canopy height, distance to main river, and NDVI in the variables on the occurrence probability in the Mae Hong Son chicken study. The optimal environmental conditions for the occurrence probability of the Mae Hong Son chicken (MaxEnt model response curves) in the study area are presented in Figure S4. The model predicted that the Mae Hong Son chicken is likely to occur in areas with elevation ranging between 200 and 300 m, a forest canopy height ranging from 2 to 6 m (shrub), a distance of 100 m from the main river, and NDVI between 0.16 and 0.17 (barren areas). The AUC value of the Maxent model was 0.974, indicating its effectiveness in predicting the potential distribution of the chicken population. The Jackknife method was applied to identify the weight of different environmental factors affecting the land suitability for the Mae Hong Son chicken (Figure S5). The results revealed that tree canopy cover, elevation, forest canopy height, distance to water, and NDVI contributed 49.2%, 37.5%, 9.8%, and 3.5%, respectively, to the potential distribution of the chicken population. Elevation was identified as the most important factor affecting the potential distribution of the Mae Hong Son chicken, due to its high contribution rate.

### 3.2. Genetic Variability Amongst Mae Hong Son Populations Based on Mitochondrial Haplotype Analysis

**Figure 4:**
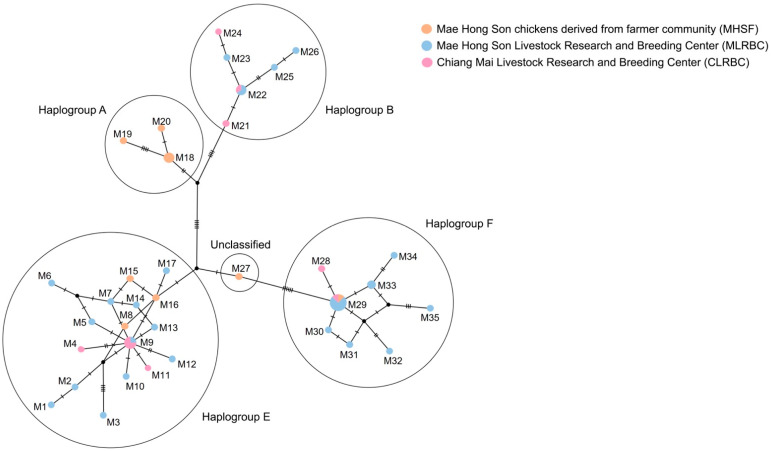
A haplotype network was constructed using sequence data from the mitochondrial D-loop region of Mae Hong Son chickens (*Gallus gallus*, [2]), derived from the Mae Hong Son farmer community, Mae Hong Son, Thailand (MHSF), Mae Hong Son Livestock Research and Breeding Center, Mae Hong Son, Thailand (MLRBC), and Chiang Mai Livestock Research and Breeding Center, Chiang Mai, Thailand (CLRBC).

The amplicon and alignment lengths of the mt D-loop sequences of 35 haplotypes from the 3 Mae Hong Son populations were 1200 bp and 1000–1200 bp, respectively. The overall haplotype and nucleotide diversities in mt D-loop sequences were 0.967 ± 0.016 and 0.014 ± 0.007, respectively (Table 1). A complex haplotype network was constructed using the large number of polymorphic sites and haplotypes detected. The haplotype M29, which is a part of haplogroup F, was found to be the most frequent among all populations. Different haplogroups, such as A, B, and E were detected from the characterization of haplotypes of Mae Hong Son chickens (Figure 4). In order to assess the level of genetic differentiation among the 3 populations, we used Wright’s *F*-statistics to calculate the genetic differentiation coefficient for subpopulations within each population (*F*_ST_ and *G*_ST_), the *Φ*_ST_ values from the sequence data and haplotype data, the average number of nucleotide substitutions per site between populations (*D*_xy_), and net nucleotide substitutions per site between populations (*D*_a_), the values of which ranged from 0.017 to 0.127, 0.014 to 0.029, 0.000 to 0.001, 0.009 to 0.010, and 0.000 to 0.002, respectively (Table S3). There was no statistical significance of Tajima’s D values ranging from −0.121 (*p* = 0.487) to 0.189 (*p* = 0.609) in the 3 Mae Hong Son chicken populations. The Fu and Li’s F* and D* values ranged from −0.767 (*p* = 1.000) to 0.511 (*p* = 1.000) and from −0.965 (*p* = 1.000) to 0.577 (*p* = 1.000), respectively, and were not significant. The Ramos-Onsins and Rozas’s R_2_ values ranged from 0.103 to 0.163 (Table S4). A multimodal distribution was indicated by the mismatch distribution analysis (Figure S6). The raggedness index values, which ranged from 0.009 to 0.079, were not statistically significant. The Extended Bayesian Skyline Plots (EBSPs) based on the mt D-loop sequences showed a tendency of constant population size (Figure S7).

### 3.3. Genetic Variability Amongst Mae Hong Son Populations Based on Microsatellite Data

A total of 188 alleles were identified at loci in the captive chickens (*n* = 50), and the mean number of alleles per locus was 6.714 ± 0.619 (Table 2). All allelic frequencies displayed significant deviations from the Hardy–Weinberg equilibrium of the population, and there were multiple indications of linkage disequilibrium (Tables S5–S7). Null alleles were frequently found for six loci (MCW0111, MCW0037, MCW0034, LEI0094, MCW0216, and MCW0104), and all markers listed were treated similarly. All populations exhibited a negative *F* Statistic (*F*-values). The polymorphic information content (*PIC*) of all the populations ranged from 0.000 to 0.825, and Shannon’s information index (*I*) ranged from 0.000 to 2.176 (Table S8). *H*_o_ and *H*_e_ values ranged from 0.000 to 1.000 (mean ± standard error [SE]: 0.641 ± 0.047) and from 0.000 to 0.842 (mean ± SE: 0.641 ± 0.029), respectively (Table 2; Table S8). In the MHSF population, the Welch’s t-test revealed a significant difference between *H*_o_ and *H*_e_ (*H*_o_ = 0.663 ± 0.059, *H*_e_ = 0.553 ± 0.034, *t* = 5.108, df = 0.110, *p* < 0.01), MLRBC (*H*_o_ = 0.643 ± 0.060, *H*_e_ = 0.598 ± 0.035, t = 3.548, df = 0.045, *p* < 0.01), and CLRBC (*H*_o_ = 0.621 ± 0.060, *H*_e_ = 0.537 ± 0.032, *t* = 3.906, df = 0.084, *p* < 0.01) populations. All pairwise *H*_o_ values between populations were not statistically different, whereas the pairwise *H*_e_ values between populations were statistically different except for 1 pair, MHSF–CLRBC (Table S9). The *AR* value of the population was 3.909 ± 0.969. The standard genetic diversity indices are summarized in Table 2 and Table S8.

To investigate the level of relatedness (*r*) between individual chickens in the population, a pairwise test was performed. The mean pairwise *r* values of 1225 combination pairs amongst a total of 50 Mae Hong Son chickens were −0.012 ± 0.002 (MHSF population, −0.048 ± 0.066; MLRBC population, −0.018 ± 0.040; and CLRBC population, −0.052 ± 0.150). None of the pairs exhibited *r* < −0.25 in any of the populations, whereas 1215 pairs displayed −0.25 < *r* < 0.25, 10 pairs displayed 0.25 < *r*, 45 pairs had *r* < 0.25 in MHSF, 435 pairs had *r* < 0.25 in MLRBC, 42 pairs had *r* < 0.25, and 3 pairs had 0.25 < *r* in CLRBC (Tables S10–S13). Distributions of *r* values for the Mae Hong Son chickens were not statistically different for any of the populations (Figure S8 and Table S14). The mean *F*_IS_ value was −0.088 ± 0.036 for all populations (Table S10). The individual values ranged from −0.167 to 0.007 (Tables S15–S17). However, distributions of *F*_IS_ in the MHSF, MLRBC, and CLRBC populations were significantly different from each other (Figure S9; Table S18). The *N*_e_ for individual chickens that contributed genetically to the MHSF, MLRBC, and CLRBC populations were 15.6 (95% CI: 9.6), 186.8 (95% CI: 90.0–110.6), and 1.9 (95% CI: 1.5), respectively (Table S10). The estimation of *F*_ST_ showed significant differences (*p* < 0.05) between the populations (Table S19) after 110 permutations. The analysis of molecular variance (AMOVA) showed that genetic variation was 78% amongst individual chickens within a population and 22% between populations (Table S20). Nei’s genetic distance between populations ranged from 0.193 to 0.502 (Table S21). The Wilcoxon signed-rank tests for recent population bottlenecks generated the stepwise mutation model (SMM) and a two-phased mutation model (TPM) of 0.089 and 0.212 for the MHSF population (shifted mode), 0.023 and 0.000 for the MLRBC population (normal L-shaped distribution), and 0.000 and 0.000 for the CLRBC population (shifted mode) (Table S22). The *M* ratios of the MHSF and MLRBC populations were below 0.68, reflecting no historical population decline [44] (Table 2).

The results of the model-based Bayesian clustering algorithms applied in this study using STRUCTURE showed that Mae Hong Son chickens exhibited different population structure patterns for various *K*-values (2–25) (Figure S10). The optimized population structure patterns were assigned to 3 clusters (*K* = 3) on the basis of Evanno’s Δ*K*; based on the mean ln P(*K*), the STRUCTURE analysis additionally identified a single peak at *K* = 3 (Figure S11). Multiple clusters of gene pools with higher *K*-values were observed in the MHSF and MLRBC populations. The results of the PCoA analysis and DAPC were in agreement with this finding, as they both indicated that all individuals tended to cluster into three primary groups (Figure S12). The gene pool of the MHSF population was closely related to that of the MLRBC population. Values of *F*_IS_ decreased with increasing *H*_e_ in a plot of *F*_IS_ versus *H*_e_, for the three Mae Hong Son chicken populations. This pattern was observed in all the microsatellite loci analyzed (Figure S13).

### 3.4. Genetic Differences between Mae Hong Son Chicken Breeds, Red Junglefowl, and Other Thai Domestic Chicken Breeds

**Figure 5:**
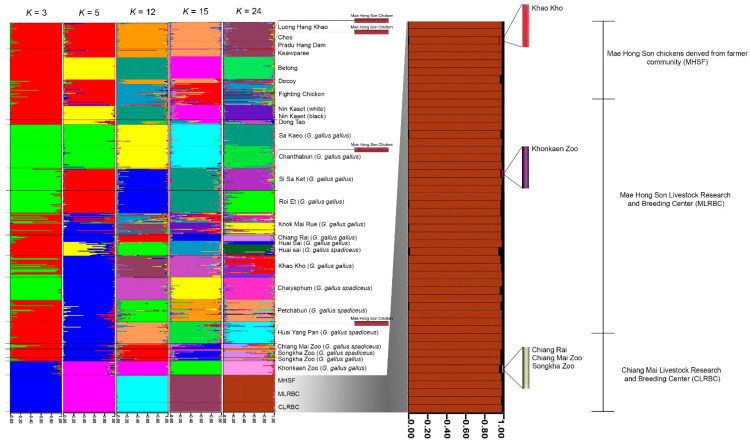
Population structure of Mae Hong Son chickens (*Gallus gallus*, [2]), red junglefowl, and domestic breeds. The proportion of membership (posterior probability) in each genetic cluster is represented by each vertical bar on the *x*-axis, while the *y*-axis shows this proportion. The plot additionally includes the Mae Hong Son chicken breed, red junglefowl, and domestic chicken breeds, which are depicted by black vertical lines to indicate their boundaries. A genetic introgression of some parts of the gene pool of red junglefowl and other indigenous breeds was identified in the Mae Hong Son chicken using a posterior probability of >0.05, as a criterion for assignment to a genetic introgression. Detailed information on each domestic chicken is presented in Table S1.

The results of the PCoA and DAPC analyses indicated multiple clusters (Figures S14 and S15). The major cluster of the gene pool contained several red junglefowl and domestic breeds; however, the Mae Hong Son chickens were grouped in different clusters of majorities. In order to evaluate the gene pool pattern of the Mae Hong Son chicken population and compare it to other domestic chicken breeds and red junglefowl, we utilized reference baseline data from previous studies [15,16]. The population structure of these chicken populations was analyzed using model-based Bayesian clustering algorithms in STRUCTURE, with *K*-values ranging from 2 to 25. The analysis revealed varying population structure patterns for the Mae Hong Son chicken population, as well as populations of domestic chickens and red junglefowl studied by Hata et al. [15] and Singchat et al. [16] (Figure 5). STRUCTURE analysis on the basis of Evanno’s Δ*K* revealed the highest posterior probability with 1 peak (*K* = 24) and that result, based on the mean ln P(*K*), additionally revealed 1 peak (*K* = 24) (Figure S16). Similar gene pool patterns were observed among most Thai indigenous chicken breeds, such as Lueng Hang Khao, Chee, or Keaw Paree, except for fighting chickens, which are considered to be Thai indigenous village chickens. Diverse gene pool patterns were observed for red junglefowl, in contrast to a unique genetic pattern (gene pool) for the Mae Hong Son chickens. However, a part of the gene pool of red junglefowl, derived from Chiang Rai, Khonkean Zoo, Chaingmai Zoo, and Songkha Zoo, was identified in the gene pool of the Mae Hong Son chickens. Moreover, the gene pool of the Mae Hong Son chickens was observed in that of indigenous chickens (Lueng Hang Khao and Chee) and red junglefowl derived from Huai Yan Pan and Chanthaburi, with no discernable genetic selective sweep observed for the Mae Hong Son or other Thai domestic chickens (Figure S17).

## 4. Discussion

### 4.1. The Mae Hong Son Chicken Has a Unique Genetic Fingerprint

The three Mae Hong Son chicken populations have retained high genetic variability, as observed from high *H*_o_ and *H*_e_ values, which were similar to those of indigenous chicken populations in Rwanda and local Swedish chickens [45,46]. Interestingly, several global indigenous chicken breeds show positive *F*_IS_ values, indicating non-random mating or the existence of substructures within populations [47,48,49]. This was not observed in the Mae Hong Son chicken populations in this study. The high genetic diversity and low inbreeding value of the Mae Hong Son chicken populations were probably due to the high number of alleles, owing to the free-range management system, which allowed a mixing of chickens across neighboring households [50,51]. This corresponds with the storyboard of free-range chickens in the local civilization and community, as described in Ismail [52]. The free-range management systems for locally raised chickens are cost-effective and promoted by the Thai government through the Department of Livestock Development to ensure food security in rural areas. The industrialization of poultry farming currently poses a threat to the local poultry system; however, it is not a concern in rural areas due to high production and maintenance costs and the local communities’ preference for free-range, locally raised chicken. The *H*_o_ values of the three populations were significantly higher than the *H*_e_ values, suggesting a history of non-selective mating amongst the Mae Hong Son chickens in all the populations studied. Additionally, the proportion of *N*_e_ and N was likely to be higher than 1 (1.0) in both MHSF and MLRBC populations, which corresponds with the negative *F* values without subdivision in each population. This indicates the high quality of the chicken population as a genetic resource. Only the CLRBC population showed results to suggest that they experienced recent and historic bottlenecks, based on microsatellites and mt D-loop sequences. The Mae Hong Son chickens of the CLRBC population were derived from the MLRBC, which was probably a small founder population, 15 years ago (Surachai Piangporntip, personal communication). This might have resulted in the proportions of *N*_e_ and N, which were less than one in the CLRBC population. However, the CLRBC chicken population has now expanded and become nearly stable, as observed from the haplotype complex network, mismatch distribution, raggedness index, and EBSPs.

The genetic differentiation (*F*_ST_) identified between the Mae Hong Son chicken populations was similar to the clustering analyses of PCoA and DAPC. The Mae Hong Son chickens of CLRBC were derived from MLRBC, and yet population differentiation could be observed between these. This suggests that the CLRBC population has already become genetically differentiated after separating from the MLRBC population. The MHSF and MLRBC populations, which were derived from the same area in Thailand, shared the same group of gene pools at *K* = 2 and 3. The allelic gene pool patterns of MLRBC contained the highest genetic diversity, possibly due to the application of sustainable management methods, such as mating between several different parental stocks by the Department of Livestock Development, Ministry of Agriculture and Cooperatives, to maintain the Mae Hong Son chicken stock. A clustering analysis of the gene pools of the three Mae Hong Son chicken populations suggested that these might be representatives of the Mae Hong Son chicken, free from the influence of genetic drift that causes the loss of genetic diversity. We, thus, analyzed diverse populations of the Mae Hong Son chickens, hypothesizing that true signals generated by selection would overlap across populations/breeds. This information on the Mae Hong Son chicken gene pools was compared with those of red junglefowl and other Thai domestic chickens, considering all the Mae Hong Son chicken populations as the same breed. Subsequently, a unique gene pool pattern of the Mae Hong Son chicken was obtained (Figure 5). The Mae Hong Son chicken might have undergone selective pressure, due to niche environmental factors under the domestication process [11,53,54,55]. The Mae Hong Son chicken breeds now exist and thrive after a long period of artificial as well as natural selection, indicating that they can be raised in varied geographical environments. The forest canopy height is the key environmental factor of habitat suitability for the Mae Hong Son chicken, similar to that of red junglefowl [16]; however, the Mae Hong Son chicken inhabits areas with elevations of 200–300 m, which differs from that of other Thai domestic chickens.

### 4.2. Genetic Footprints of the Red Junglefowl and Thai Domestic Chicken Were Observed in Mae Hong Son Chicken despite Large Differences between Their Gene Pool Patterns

Recent research on the gene pools of red junglefowl and the Thai domestic chicken revealed a high genetic diversity in the red junglefowl, which is widely distributed across different geographical areas in Thailand [15,16]. To compare the gene pool of the Mae Hong Son chickens with the original genetic source of red junglefowl and Thai domestic chickens, the microsatellite and mt D-loop sequence data obtained in this study were compared to the reference gene pool library established during the “Siam Chicken Bioresource Project” by Hata et al. [15] and Singchat et al. [16]. The haplogroups of the Mae Hong Son chickens observed in this study were classified into haplogroups A, B, E, and F. Haplogroups A–E and J were the common haplogroups of red junglefowl in Thailand, in contrast to other haplogroups that have been found in other regions of Asia (G, China; I, India; K, Indonesia; and W–Z, China) [15,16,56]. However, most dominant haplotypes of the Mae Hong Son chicken were assigned to haplogroup F, which was mainly identified in Southwest China and Myanmar [57]. This finding fits with the significant socio–cultural role of the Mae Hong Son chicken as a traditional offering to spirits in the local communities, across the boundary between Northern Thailand and Myanmar [52]. These results suggest that the Mae Hong Son chicken’s origins are associated with local communities in Northwest Thailand, following the genetic origin of the North ecotype of red junglefowl based on microsatellite genotyping data. The Mae Hong Son chicken populations formed an independent cluster with a unique gene pool pattern (Figures S14 and S15). This was additionally evident from the specific phenotypic characteristics of this indigenous chicken, which are different from those in other indigenous chickens in Thailand. The Mae Hong Son chickens are distinguished by the dark yellow color of their neck and back hairs [52]. However, based on microsatellite data, a few components of the gene pool of the Mae Hong Son chicken were shared with the gene pool of red junglefowl derived from Chiang Rai, Khonkaen Zoo, Chaingmai Zoo, and Songkha Zoo, whereas the allelic gene pattern of the Mae Hong Son chickens was found in indigenous chickens (Luang Hang Khao and Chee) and red junglefowl derived from Huai Yan Pan and Chanthaburi. This matches with some haplotypes of the Mae Hong Son chickens, belonging to haplogroup E, which were identified with the haplogroup of red junglefowl from Huai Yang Pan (Chiang Mai, Northern Thailand). This suggests that the Mae Hong Son chicken was originally established from the north ecotype of red junglefowl which inhabits North Thailand. However, a few ecotypes found in Mae Hong Son chickens, which were different from those of Northeast, East, and Southern Thailand may be a consequence of the large gene pools of red junglefowl across Thailand [16]. The sharing of gene pools between red junglefowl and the Mae Hong Son chickens is probably the genetic footprint of natural selection. This concurs with our hypothesis that the Mae Hong Son chicken possesses some components of the gene pool (or genetic footprint) of the Northern ecotype of red junglefowl. Notwithstanding, several Thai indigenous chickens, including Lueng Hang Khao, Chee, Keawparee, and Fighting Chicken displayed the same clustering pattern along different *K* values, except for the Mae Hong Son chickens based on the result of the STRUCTURE plot, PCoA and DAPC. This might result from the long history of selective breeding in these indigenous chickens, with an introgression of red junglefowl. Luang Hang Khao or Chee, Pradu Hang Dam and Keawparee are indigenous breeds distributed across the country and likely underwent genetic exchange with large populations of the domestic chicken breed or red junglefowl. In contrast, the Mae Hong Son or Betong chickens are located in specific regions (Mae Hong Son chickens in the northern regions and Betong chickens in the southern). This might result in the independent cluster of the reference gene pool library. However, a small part of the gene pool of the Mae Hong Son chicken was shared with the Luang Hang Khao and Chee breeds which are believed to have originated from Thai indigenous village chickens [57], suggesting an intermediate process of domestication prior to intense human-mediated selection for the Luang Hang Khao or Chee breeds as ornamental indigenous chickens [17].

Genetic selection is the main force that drives domestication and the genetic improvement of agricultural animals [58]. However, no genetic selective sweep was found in the Mae Hong Son chickens, possibly resulting from the insufficient duration of the domestication process and selection of Mae Hong Son chickens to produce high genetic homogeneity by genetic selective sweep, compared with other domestic animals [59]. Surprisingly, based on the results of microsatellite genotyping in this study, none of the Thai domestic chickens underwent a genetic selective sweep, even though selective breeding was carried out for some breeds. This may have resulted from the insufficient number of microsatellite markers used in this study, as they are positioned at fixed intervals and may not cover the entire genome adequately. The sample size was small, and the genetic hitchhiking effects of the loci under selection were considered to be minimal in the case of the Mae Hong Son chickens. In addition, the 28 microsatellite markers used in this study may have introduced bias due to factors such as the timing of selection, limited population history, phasing errors, and false LD resolution [60]. Genome-wide microsatellites in a pair of the subspecies *Mus musculus domesticus* and *M. musculus musculus*, *Perisoreus infaustus*, and laying chickens were used to investigate the occurrence of genetic selective sweep [61,62,63,64]. Therefore, to thoroughly examine the evidence of selective sweep in Thai indigenous chicken breeds, it is necessary to increase the sample sizes and use a larger number of microsatellite loci. Genotyping by microsatellite loci could be more informative than the use of biallelic single nucleotide polymorphisms (SNPs) to resolve the possible selective sweep and balance selection in the population, because microsatellite loci (mutational hot spots) display high polymorphism with a larger number of alleles (multi-allele) in diverse populations [65].

The Mae Hong Son chickens inhabit high mountain complexes and rich, natural forests that experience three distinct seasons: the hot season (mid-February to mid-May), rainy season (mid-May to mid-October), and cool season (mid-October to mid-February). The maximum and minimum temperatures in the region are 44.6 °C (112.3 °F) and 3.9 °C (39.0 °F), respectively, with highest and lowest averages of 35.6 °C (96.1 °F) and 17.98 °C (64.4 °F), respectively. The highest relative humidity is 83%, and the lowest relative humidity is 55%. The annual rainfall in the region is 1064.9 mm, with the highest value of 130.4 mm in 24 h, the number of rainy days being 130 per annum [66,67]. The Mae Hong Son chickens have been bred in low-input production systems, and unique genetic variants could have accumulated in the Mae Hong Son chickens as the result of adaptation to the climate and management conditions in their habitat. Therefore, these chickens are a highly valuable resource with useful genetic variations. The conservation of the Mae Hong Son chickens enables a sustainable utilization of a highly useful poultry resource and provides an ideal model for genetic improvement to establish breeds/lines that are adapted to specific environments and production systems in Thailand. Additional research is required to better comprehend the nutritional characteristics of the Mae Hong Son chicken in comparison to other breeds, as well as their relationship to diverse phenotypic variations and gene pools. Such information is crucial for developing genetic improvement programs with commercial chickens, or other domestic chicken breeds. This will additionally confirm their genetic wealth, which is conserved within domestic chicken populations and ensure that their genetic diversity is maintained.

## 5. Conclusions

In conclusion, we investigated the genetic diversity of the Thai domestic chicken breed, known as Mae Hong Son, to address the hypothesis of its origin as a crossbreed of red junglefowl and Thai indigenous village chickens that adapted to the environmental, social, and cultural conditions in its habitat. Our findings suggest that due to continuous selective breeding, the Mae Hong Son chickens have become genetically and geographically isolated. Our data do not reveal any evident patterns of recent admixture, which suggests that the Mae Hong Son chickens may have remained relatively selectively bred over time. However, this does not necessarily rule out the traditional view that the Mae Hong Son chickens originated from the northern ecotypes of red junglefowl in Thailand. Our predicted models re-emphasize that the availability of food and water determined the habitat of the Mae Hong Son chickens. The high genetic variability of the Mae Hong Son chickens, along with different ecological factors, drove the adaptation of the Mae Hong Son chickens under selective pressure. These findings enrich our understanding of the genetic blueprint, origin, and evolutionary process of the Mae Hong Son chickens and lay the foundation for future studies to improve domestic chickens using this indigenous chicken breed. To further support this hypothesis about the genetic ancestry of the Mae Hong Son chickens, we recommend additional analysis and the use of statistical models based on genome-wide SNPs. These methods will provide a more comprehensive understanding of the genetic makeup and origins of the Mae Hong Son chickens.

## Figures and Tables

**Table 1:** Mitochondrial D-loop sequence diversity in Mae Hong Son chickens (*Gallus gallus*, [2]).

Population	N	Number of Haplotypes (*H*)	Theta (Per Site) from *S*	Average Number of Nucleotide Differences (*k*)	Overall Haplotype (*h*)	Nucleotide Diversities (π)
MHSF ^1^	10	10	0.008	7.822	1.000 ± 0.045	0.011 ± 0.006
MLRBC ^2^	30	23	0.010	9.844	0.961 ± 0.027	0.014 ± 0.007
CLRBC ^3^	10	9	0.010	10.356	0.978 ± 0.054	0.013 ± 0.007
All populations	50	35	0.010	9.598	0.967 ± 0.016	0.014 ± 0.007

^1^ MHSF = Mae Hong Son chickens derived from farmer community, Mae Hong Son. ^2^ MLRBC = Mae Hong Son Livestock Research and Breeding Center, Mae Hong Son. ^3^ CLRBC = Chiang Mai Livestock Research and Breeding Center, Chiang Mai.

**Table 2:** Genetic diversity amongst individual Mae Hong Son chickens (*n* = 50) (*Gallus gallus*, [2]), based on 28 microsatellite loci.

Population		*N*_a_ ^1^	*AR* ^2^	*N*_ea_ ^3^	*I* ^4^	*H*_o_ ^5^	*H*_e_ ^6^	*M* Ratio ^7^	*PIC* ^8^	*F* ^9^
MHSF ^10^	Mean	3.679	3.619	2.562	1.002	0.663	0.553	0.400	0.497	−0.181
	S.E.	0.225	1.167	0.179	0.070	0.059	0.034	0.277	0.173	0.064
MLRBC ^11^	Mean	5.464	5.414	2.955	1.189	0.643	0.598	0.390	0.552	−0.061
	S.E.	0.500	2.624	0.238	0.085	0.060	0.035	0.246	0.180	0.068
CLRBC ^12^	Mean	3.071	2.693	2.411	0.913	0.621	0.537	0.744	0.468	−0.120
	S.E.	0.212	0.788	0.155	0.066	0.060	0.032	0.834	0.165	0.098
Total	Mean	6.714	3.909	3.293	1.334	0.641	0.641	0.281	0.599	−0.006
	S.E.	0.619	0.969	0.261	0.084	0.047	0.029	0.132	0.160	0.057

^1^ Number of alleles (*N*_a_); ^2^ Allelic richness (*AR*); ^3^ Number of effective alleles (*N*_ea_); ^4^ Shannon’s information index (*I*); ^5^ Observed heterozygosity (*H*_o_); ^6^ Expected heterozygosity (*H*_e_); ^7^ *M* ratio test (*M* ratio); ^8^ Polymorphic information content (*PIC*); and ^9^ Fixation index (*F*). ^10^ MHSF = Mae Hong Son chickens derived from farmer community, Mae Hong Son; ^11^ MLRBC = Mae Hong Son Livestock Research and Breeding Center, Mae Hong Son; and ^12^ CLRBC = Chiang Mai Livestock Research and Breeding Center, Chiang Mai.

## Data Availability

The datasets generated in this study can be found in the DNA Data Bank of Japan (DDBJ) (https://www.ddbj.nig.ac.jp/, accessed on 10 October 2022) (accession number: LC731861–LC731880 and LC731886–LC731915). Microsatellite genotypic data can be accessed from the Dryad Digital Repository Dataset (https://datadryad.org/stash/share/x2qlPmboMgCROXO8kQjFc5wF6INadoYmciW1y3WRJ88, accessed on 17 October 2022). The genotypic data of red junglefowl and Thai domestic chicken breeds/ecotypes can be accessed from the Dryad Digital Repository Dataset (https://datadryad.org/stash/share/x2qlPmboMgCROXO8kQjFc5wF6INadoYmciW1y3WRJ88, accessed on 17 October 2022).

## Supplementary Materials

The following supporting information can be downloaded at https://www.mdpi.com/article/10.3390/ani13121949/s1. Figure S1: environmental variables used to assess the species distribution model of Mae Hong Son chicken: (A) elevation, (B) distance to water, (C) normalized difference vegetation index (NDVI) and (D) forest canopy height; Figure S2: phenotypic characteristics of male (A–B) and female (C–D) Mae Hong Son chicken; Figure S3: potential land suitability modeling of Mae Hong Son chickens in Thailand; Figure S4: MaxEnt model response area curves of the predictor variables influencing wild chicken distribution. (A) elevation, (B) forest canopy height, (C) distance to main river and (D) normalized difference vegetation index (NDVI); Figure S5: the Jackknife method used in maximum entropy modeling and environmental factors affecting the potential distribution of Mae Hong Son chickens; Figure S6: Mismatch distributions analysis of Mae Hong Son chickens derived from (A) the Mae Hong Son farmer community, Mae Hong Son, Thailand (MHSF), (B) Mae Hong Son Livestock Research and Breeding Center, Mae Hong Son, Thailand (MLRBC), or (C) Chiang Mai Livestock Research and Breeding Center, Chiang Mai, Thailand (CLRBC). The x- and y-axes represent the number of pairwise differences (mismatches) and the frequency of these differences, respectively. The observed mismatch distribution (red line) is compared to the expected distribution (green line) for a stable population; Figure S7: Output of the Coalescent Bayesian Skyline analysis. The black line represents the median estimated effective population size. Blue areas represent the upper and lower bounds. The x- and y-axes represent the time in years and a log scale, respectively. Mae Hong Son chickens derived from (A) the Mae Hong Son farmer community, Mae Hong Son, Thailand (MHSF), (B) Mae Hong Son Livestock Research and Breeding Center, Mae Hong Son, Thailand (MLRBC), and (C) Chiang Mai Livestock Research and Breeding Center, Chiang Mai, Thailand (CLRBC); Figure S8: observed distribution of relatedness (*r*) in Mae Hong Son chickens (*n* = 50) (*Gallus gallus*, [2]), plotted against expected distributions; Figure S9: observed distribution of inbreeding coefficients (*F*_IS_) in Mae Hong Son chickens (*n* = 50) (*Gallus gallus* [2]), plotted against expected distributions; Figure S10: population structure of Mae Hong Son chickens (*n* = 50) (*Gallus gallus*, [2]). Each vertical bar on the x-axis represents an individual chicken; the y-axis represents the proportion of membership (posterior probability) in each genetic cluster. Red junglefowl are superimposed on the plot, with black vertical lines indicating the boundaries. Detailed information on each domestic chicken is presented in Table S1; Figure S11: different population structure patterns of Mae Hong Son chickens (*n* = 50) (*Gallus gallus*, [2]), generated by model-based Bayesian clustering algorithms implemented in STRUCTURE. (A) plot of Evanno’s Δ*K* and (B) plot of ln P(*K*); Figure S12: (A) principal component analysis of Mae Hong Son chickens (*Gallus gallus*, [2]) and (B) the results of the discriminant analysis of principal components (DAPC) of three indigenous chicken populations, derived from the Mae Hong Son farmer community, Mae Hong Son, Thailand (MHSF), Mae Hong Son Livestock Research and Breeding Center, Mae Hong Son, Thailand (MLRBC), and Chiang Mai Livestock Research and Breeding Center, Chiang Mai, Thailand (CLRBC). Scatter plots based on the DAPC output for three assigned genetic clusters are indicated by different colors. Dots represent different individuals. Detailed information on each domestic chicken is presented in Table S1; Figure S13: mapping of expected heterozygosity (*H*_e_) against inbreeding coefficients (*F*_IS_), along the length of the physical map. (A) Mae Hong Son chicken populations and (B) microsatellite loci; Figure S14: principal component analysis of Mae Hong Son chickens (*Gallus gallus*, [2]), derived from the Mae Hong Son farmer community, Mae Hong Son, Thailand (MHSF), Mae Hong Son Livestock Research and Breeding Center, Mae Hong Son, Thailand (MLRBC), and Chiang Mai Livestock Research and Breeding Center, Chiang Mai, Thailand (CLRBC) with red junglefowl and domestic chicken breeds; Figure S15: Discriminant analysis of principal components (DAPC) of indigenous chicken populations derived from the Mae Hong Son farmer community, Mae Hong Son, Thailand (MHSF), Mae Hong Son Livestock Research and Breeding Center, Mae Hong Son, Thailand (MLRBC), and Chiang Mai Livestock Research and Breeding Center, Chiang Mai, Thailand (CLRBC) with red junglefowl and domestic breeds. Scatter plots based on the DAPC output for three assigned genetic clusters are indicated by different colors. Dots represent different individuals; Figure S16: different population structure patterns of Mae Hong Son chickens (*n* = 50) (*Gallus gallus*, [2]), red junglefowl, and domestic chicken breeds, generated by the model-based Bayesian clustering algorithms implemented in STRUCTURE. (A) plot of Evanno’s Δ*K* and (B) plot of ln P(*K*); Figure S17: mapping of expected heterozygosity (*H*_e_) against inbreeding coefficients (*F*_IS_), along the length of the physical map. (A) Mae Hong Son chickens breed, red junglefowl and domestic chicken breeds and (B) microsatellite loci; Table S1: detailed information on the specimens from three populations of Mae Hong Son chickens (*Gallus gallus*, [2]) in Thailand. All sequences were deposited in the DNA Data Bank of Japan (DDBJ); Table S2: microsatellite primers and sequences of Mae Hong Son chicken specimens; Table S3: genetic differentiation between the three populations of Mae Hong Son chickens (*Gallus gallus*, [2]), based on the mitochondrial D-loop sequence. *G*_ST_: Genetic differentiation coefficient, *F*_ST_: Wright’s *F*-statistics for subpopulations within the total population, *Φ*_ST_: from sequence and haplotype data, *D*_xy_: the average number of nucleotide substitutions per site between populations, and *D*_a_: net nucleotide substitutions per site between populations; Table S4: neutrality tests of a mitochondrial D-loop sequence of Mae Hong Son chickens (*Gallus gallus*, [2]); Table S5: pairwise differentiation of linkage disequilibrium of Mae Hong Son chickens (*Gallus gallus*, [2]), derived from the Mae Hong Son farmer community, Mae Hong Son, Thailand (MHSF), based on 28 microsatellite loci. Numbers indicate *p*-values with 110 permutations; Table S6: pairwise differentiation of linkage disequilibrium of Mae Hong Son chickens (*Gallus gallus*, [2]) from Mae Hong Son Livestock Research and Breeding Center, Mae Hong Son, Thailand (MLRBC), based on 28 microsatellite loci. Numbers indicate *p*-values with 110 permutations; Table S7: pairwise differentiation of linkage disequilibrium of Mae Hong Son chickens (*Gallus gallus*, [2]) from Chiang Mai Livestock Research and Breeding Center, Chiang Mai, Thailand (CLRBC), based on 28 microsatellite loci. Numbers indicate *p*-values with 110 permutations; Table S8: genetic diversity of Mae Hong Son chickens (*n* = 50) (*Gallus gallus*, [2]), based on 28 microsatellite loci. Detailed information on each individual chicken is presented in Table S1; Table S9: comparison of genetic diversity parameters amongst Mae Hong Son chicken (*Gallus gallus*, [2]) populations, based on 28 microsatellite loci; Table S10: inbreeding coefficients (*F*_IS_), relatedness (*r*), effective population size (*N*_e_), and ratio of effective population size to census population (*N*_e_/N) of Mae Hong Son chickens (*Gallus gallus*, [2]) derived from the Mae Hong Son farmer community, Mae Hong Son, Thailand (MHSF), Mae Hong Son Livestock Research and Breeding Center, Mae Hong Son, Thailand (MLRBC), and Chiang Mai Livestock Research and Breeding Center, Chiang Mai, Thailand (CLRBC). Estimates were calculated using NeEstimator version 2.1 [68], COANCESTRY version 1.0.1.9 [69], and GenAlEx version 6.5 [70]. Detailed information on each Mae Hong Son chicken is presented in Table S1; Table S11: pairwise genetic relatedness (*r*) amongst Mae Hong Son chicken (*Gallus gallus*, [2]) populations, derived from the Mae Hong Son farmer community, Mae Hong Son, Thailand (MHSF). Detailed information on each individual chicken is presented in Table S1; Table S12: pairwise genetic relatedness (*r*) amongst Mae Hong Son chickens (*Gallus gallus*, [2]) populations from Mae Hong Son Livestock Research and Breeding Center, Mae Hong Son, Thailand (MLRBC). Detailed information on each individual chicken is presented in Table S1; Table S13: Pairwise genetic relatedness (*r*) amongst Mae Hong Son chickens (*Gallus gallus*, [2]) populations from Chiang Mai Livestock Research and Breeding Center, Chiang Mai, Thailand (CLRBC). Detailed information on each individual chicken is presented in Table S1; Table S14: relatedness (*r*) distributions of Mae Hong Son chickens (*Gallus gallus*, [2]); Table S15: Inbreeding coefficients (*F*_IS_) of Mae Hong Son chickens (*n* = 10) (*Gallus gallus*, [2]), derived from the Mae Hong Son farmer community, Mae Hong Son, Thailand (MHSF). Detailed information on each individual chicken is presented in Table S1; Table S16: Inbreeding coefficients (*F*_IS_) of Mae Hong Son chickens (*n* = 30) (*Gallus gallus*, [2]) from Mae Hong Son Livestock Research and Breeding Center, Mae Hong Son, Thailand (MLRBC). Detailed information on each individual chicken is presented in Table S1; Table S17: inbreeding coefficients (*F*_IS_) of Mae Hong Son chickens (*n* = 10) (*Gallus gallus*, [2]) from Chiang Mai Livestock Research and Breeding Center, Chiang Mai, Thailand (CLRBC). Detailed information on each individual chicken is presented in Table S1; Table S18: *F*_IS_ distributions for Mae Hong Son chickens (*Gallus gallus*, [2]); Table S19: pairwise genetic differentiation (*F*_ST_), pairwise *F*_ST_^ENA^ values with ENA correction for null alleles, and *R*_ST_ values using FSTAT version 2.9.3 [71] for Mae Hong Son chickens (*Gallus gallus*, [2]) subjected to captive breeding, based on 28 microsatellite loci. Numbers indicate *p*-values with 110 permutations. Detailed information on each individual chicken is presented in Table S1; Table S20: Analysis of molecular variance (AMOVA) for Mae Hong Son chickens (*Gallus gallus*, [2]), based on 28 microsatellite loci, using Arlequin version 3.5.2.2 [72]. Detailed information on each individual chicken is presented in Table S1; Table S21: results of pairwise population Nei’s genetic distance (*D*) in Mae Hong Son chickens (*n* = 50) (*Gallus gallus*, [2]), based on 28 microsatellite loci, using GenAlEx version 6.5 [70]; and Table S22: observed and expected heterozygosity in Mae Hong Son chickens (*Gallus gallus*, [2]), based on 28 microsatellite loci and genetic bottlenecks of each individual chicken. Data were calculated using Bottleneck version 1.2.02 [73]. Detailed information on each Mae Hong Son chicken is presented in Table S1. References [2,3,15,16,29,32,35,44,68,69,70,71,72,73,74,75,76,77,78,79,80,81,82,83,84,85,86,87,88,89,90,91,92,93,94,95,96,97,98,99,100,101,102,103] are cited in the supplementary materials (supplementary Data, figures caption and tables caption).

## References

[1] Mammo M. (2013). Biophysical and the socio-economics of chicken production. Afr. J. Agric. Res..

[2] Linnaeus C., Tomus I. (1798). Systema Naturæ per Regna tria Naturæ, Secundum Classes, Ordines, Genera, Species, cum Characteribus, Differentiis, Synonymis, Locis.

[3] Sawai H., Kim H.L., Kuno K., Suzuki S., Gotoh H., Takada M., Takahata N., Satta Y., Akishinonomiya F. (2010). The origin and genetic variation of domestic chickens with special reference to junglefowls *Gallus g. gallus* and *G. varius*. PLoS ONE.

[4] Sauer C.O. (1952). Agricultural Origins and Dispersals.

[5] Zeuner E.F. (1963). A History of Domesticated Animals.

[6] Issac E. (1970). Geography of Domestication.

[7] Crawford R.D. (1990). Poultry Genetic Resources: Evolution, Diversity, and Conservation.

[8] Smith P., Daniel C. (2000). The Chicken Book.

[9] Ekarius C. (2007). Storey’s Illustrated Guide to Poultry Breeds.

[10] Aini I.T. (1990). Indigenous chicken production in South-east Asia. Poult. Sci. J..

[11] Bettridge J.M., Psifidi A., Terfa Z.G., Desta T.T., Lozano-Jaramillo M., Dessie T., Kaiser P., Wigley P., Hanotte O., Christley R.M. (2018). The role of local adaptation in sustainable production of village chickens. Nat. Sustain..

[12] Schütz K.E., Forkman B., Jensen P. (2001). Domestication effects on foraging strategy, social behaviour and different fear responses: A comparison between the red junglefowl (*Gallus gallus*) and a modern layer strain. Appl. Anim. Behav. Sci..

[13] Keeling L., Andersson L., Schütz K.E., Kerje S., Fredriksson R., Carlborg Ö., Cornwallis C.K., Pizzari T., Jensen P. (2004). Feather-pecking and victim pigmentation. Nature.

[14] Tixier-Boichard M., Bed’hom B., Rognon X. (2011). Chicken domestication: From archeology to genomics. C. R. Biol..

[15] Hata A., Nunome M., Suwanasopee T., Duengkae P., Chaiwatana S., Chamchumroon W., Suzuki T., Koonawootrittriron S., Matsuda Y., Srikulnath K. (2021). Origin and evolutionary history of domestic chickens inferred from a large population study of Thai red junglefowl and indigenous chickens. Sci. Rep..

[16] Singchat W., Chaiyes A., Wongloet W., Ariyaraphong N., Jaisamut K., Panthum T., Ahmad S.F., Chaleekarn W., Suksavate W., Inpota M. (2022). Red junglefowl resource management guide bioresource reintroduction for sustainable food security in Thailand. Sustainability.

[17] Dessie T., Taye T., Dana N., Ayalew W., Hanotte O. (2011). Current state of knowledge on phenotypic characteristics of indigenous chickens in the tropics. Poult. Sci. J..

[18] Mengesha M. (2012). Indigenous chicken production and the innate characteristics. Asian J. Poult. Sci..

[19] Kroeksakul P., Ngamniyom A., Suthisaksophon P., Srichaiwong P., Pheangthai D. (2020). Stability of Native Chicken Raising Systems: The Case of Lawa Ethnic in Mae Hong Son Province, Thailand. J. Community Mobilization Sustain. Dev..

[20] Rakbankerd (2012). Mae Hong Son. https://www.rakbankerd.com/agriculture/print.php?id=2850&s=tblanimal.

[21] Leotaragul A., Sophonchit S., Veerasmith P., Saithong S. (2005). Selection and Improvement Regional Native Chickens (Maehongson chicken) for Raising in the Northern Highland of Thailand.

[22] Charoensook R., Tartrakoon W., Incharoen T., Numthuam S., Pechrkong T., Nishibori M. (2021). Production system characterization of local indigenous chickens in lower Northern Thailand. Khon Kaen Agr. J..

[23] Phillips S.J., Anderson R.P., Schapire R.E. (2006). Maximum entropy modeling of species geographic distributions. Ecol. Modell..

[24] Northern Thailand Cultural Encyclopedia (1999). Bangkok: Thai Cultural Encyclopedia Foundation.

[25] Phansuk A. (2009). Chicken and Lanna Folklife.

[26] Prapattong P. (2016). Fighting Cocks as Intangible Cultural Heritage of Upper-Northern Thailand.

[27] Hansen M.C., Potapov P.V., Moore R., Hancher M., Turubanova S.A., Tyukavina A., Thau D., Stehman S.V., Goetz S.J., Loveland T.R. (2013). High-resolution global maps of 21st-century forest cover change. Science.

[28] Potapov P., Hansen M.C., Kommareddy I., Kommareddy A., Turubanova S., Pickens A., Adusei B., Tyukavina A., Ying Q. (2020). Landsat analysis ready data for global land cover and land cover change mapping. Remote Sens..

[29] Phillips S.J., Dudík M. (2008). Modeling of species distributions with Maxent: New extensions and a comprehensive evaluation. Ecography.

[30] Elith J., Phillips S.J., Hastie T., Dudík M., Chee Y.E., Yates C.J. (2011). A statistical explanation of MaxEnt for ecologists. Divers. Distrib..

[31] Elith J., Graham C.H., Anderson R.P., Dudík M., Ferrier S., Guisan A., Hijmans R.J., Huettmann F., Leathwick J.R., Lehmann A. (2006). Novel methods improve prediction of species’ distributions from occurrence data. Ecography.

[32] Pearson R.G., Raxworthy C.J., Nakamura M., Townsend Peterson A. (2007). Predicting species distributions from small numbers of occurrence records: A test case using cryptic geckos in Madagascar. J. Biogeogr..

[33] Wisz M.S., Hijmans R.J., Li J., Peterson A.T., Graham C.H., Guisan A. (2008). Effects of sample size on the performance of species distribution models. Divers. Distrib..

[34] Supikamolseni A., Ngaoburanawit N., Sumontha M., Chanhome L., Suntrarachun S., Peyachoknagul S., Srikulnath K. (2015). Molecular barcoding of venomous snakes and species-specific multiplex PCR assay to identify snake groups for which antivenom is available in Thailand. Genet. Mol. Res..

[35] Fielding A.H., Bell J.F. (1997). A review of methods for the assessment of prediction errors in conservation presence/absence models. Environ. Conserv..

[36] Nishibori M., Hayashi T., Tsudzuki M., Yamamoto Y., Yasue H. (2001). Complete sequence of the Japanese quail (*Coturnix japonica*) mitochondrial genome and its genetic relationship with related species. Anim. Genet..

[37] Ariyaraphong N., Ho My Nguyen D., Singchat W., Suksavate W., Panthum T., Langkaphin W., Chansitthiwet S., Angkawanish T., Promking A., Kaewtip K. (2022). Standard identification certificate for legal legislation of a unique gene pool of Thai domestic elephants originating from a male elephant contribution to breeding. Sustainability.

[38] Budi T., Singchat W., Tanglertpaibul N., Wongloet W., Chaiyes A., Ariyaraphong N., Thienpreecha W., Wannakan W., Mungmee A., Thong T. (2023). Thai Local Chicken Breeds, Chee Fah and Fah Luang, Originated from Chinese Black-Boned Chicken with Introgression of Red Junglefowl and Domestic Chicken Breeds. Sustainability.

[39] Huelsenbeck J.P., Ronquist F. (2021). MRBAYES: Bayesian inference of phylogenetic trees. J. Bioinform..

[40] Tanabe A.S. (2011). Kakusan4 and Aminosan: Two programs for comparing nonpartitioned, proportional and separate models for combined molecular phylogenetic analyses of multilocus sequence data. Mol. Ecol. Resour..

[41] Food and Agriculture Organization (2011). Molecular Genetic Characterization of Animal Genetic Resources.

[42] Reddy U.K., Abburi L., Abburi V.L., Saminathan T., Cantrell R., Vajja V.G., Reddy R., Tomason Y.R., Levi A., Wehner T.C. (2015). A genome-wide scan of selective sweeps and association mapping of fruit traits using microsatellite markers in watermelon. J. Hered..

[43] Royal Forest Department (2019). Information, Forest Statistics Year. https://www.forest.go.th/home/.

[44] Garza J.C., Williamson E.G. (2001). Detection of reduction in population size using data from microsatellite loci. Mol. Ecol..

[45] Abebe A.S., Mikko S., Johansson A.M. (2015). Genetic diversity of five local Swedish chicken breeds detected by microsatellite markers. PLoS ONE.

[46] Habimana R., Okeno T.O., Ngeno K., Mboumba S., Assami P., Gbotto A.A., Keambou C.T., Nishimwe K., Mahoro J., Yao N. (2020). Genetic diversity and population structure of indigenous chicken in Rwanda using microsatellite markers. PLoS ONE.

[47] van Marle-Köster E., Hefer C.A., Nel L.H., Groenen M.A.M. (2008). Genetic diversity and population structure of locally adapted South African chicken lines: Implications for conservation. S. Afr. J. Anim. Sci..

[48] Hedrick P.W. (2013). Adaptive introgression in animals: Examples and comparison to new mutation and standing variation as sources of adaptive variation. Mol. Ecol..

[49] Ige A.O., Debabani R.C., Thangaraj K., Salako A.E., Utpal B. (2017). Genetic diversity among fulani and yoruba ecotype of Nigeria indigenous chicken in the derived savannah zone using microsatellite markers. J. Eng. Technol..

[50] Silva V.P., van der Werf H.M., Soares S.R., Corson M.S. (2014). Environmental impacts of French and Brazilian broiler chicken production scenarios: An LCA approach. Environ. Manag..

[51] Ghayas A., Hussain J., Mahmud A., Jaspal M.H. (2020). Evaluation of three fast-and slow-growing chicken strains reared in two production environments. S. Afr. J. Anim. Sci..

[52] Ismail J. (2008). Ethnic Tourism and the Kayan Long-Neck Tribe in Mae Hong Son, Thailand. Ph.D. Thesis.

[53] Birhanu M.Y., Alemayehu T., Bruno J.E., Kebede F.G., Sonaiya E.B., Goromela E.H., Bamidele O., Dessie T. (2021). Technical efficiency of traditional village chicken production in Africa: Entry points for sustainable transformation and improved livelihood. Sustainability.

[54] Gheyas A.A., Vallejo-Trujillo A., Kebede A., Lozano-Jaramillo M., Dessie T., Smith J., Hanotte O. (2021). Integrated environmental and genomic analysis reveals the drivers of local adaptation in African indigenous chickens. Mol. Biol. Evol..

[55] Kebede F.G., Komen H., Dessie T., Alemu S.W., Hanotte O., Bastiaansen J.W. (2021). Species and phenotypic distribution models reveal population differentiation in Ethiopian indigenous chickens. Front. Genet..

[56] Miao Y.W., Peng M.S., Wu G.S., Ouyang Y.N., Yang Z.Y., Yu N., Liang J.P., Pianchou G., Beja-Pereira A., Mitra B. (2013). Chicken domestication: An updated perspective based on mitochondrial genomes. Heredity.

[57] Buranawit K., Chailungka C., Wongsunsri C., Laenoi W. (2016). Phenotypic characterization of Thai native black-bone chickens indigenous to northern Thailand. Thai J. Vet. Med..

[58] Dekkers J., Hospital F. (2002). The use of molecular genetics in the improvement of agricultural populations. Nat. Rev. Genet..

[59] Elferink M.G., Megens H.J., Vereijken A., Hu X., Crooijmans R.P., Groenen M.A. (2012). Signatures of selection in the genomes of commercial and non-commercial chicken breeds. PLoS ONE.

[60] Rubin C.J., Zody M.C., Eriksson J., Meadows J.R., Sherwood E., Webster M.T., Jiang L., Ingman M., Sharpe T., Ka S. (2010). Whole-genome resequencing reveals loci under selection during chicken domestication. Nature.

[61] Qanbari S., Strom T.M., Haberer G., Weigend S., Gheyas A.A., Turner F., Burt D.W., Preisinger R., Gianola D., Simianer H. (2012). A high resolution genome-wide scan for significant selective sweeps: An application to pooled sequence data in laying chickens. PLoS ONE.

[62] Vigouroux Y., McMullen M., Hittinger C.T., Houchins K., Schulz L., Kresovich S., Matsuoka Y., Doebley J. (2002). Identifying genes of agronomic importance in maize by screening microsatellites for evidence of selection during domestication. Proc. Natl. Acad. Sci. USA.

[63] Teschke M., Mukabayire O., Wiehe T., Tautz D. (2008). Identification of selective sweeps in closely related populations of the house mouse based on microsatellite scans. Genetics.

[64] Li M.H., Merilä J. (2010). Sex-specific population structure, natural selection, and linkage disequilibrium in a wild bird population as revealed by genome-wide microsatellite analyses. BMC Evol. Biol..

[65] Qu L., Li X., Xu G., Chen K., Yang H., Zhang L., Wu G., Hou Z., Xu G., Yang N. (2006). Evaluation of genetic diversity in Chinese indigenous chicken breeds using microsatellite markers. Sci. China Life Sci..

[66] Thai Metearological Department (2022). Weather Information of Mae Hong Son. https://www.tmd.go.th/en.

[67] Weather Atlas (2022). Climate and monthly weather forecast Mae Hong Son, Thailand. https://www.weather-atlas.com/en/thailand/mae-hong-son-climate.

[68] Do C., Waples R.S., Peel D., Macbeth G.M., Tillett B.J., Ovenden J.R. (2014). NeEstimator v2: Reimplementation of software for the estimation of contemporary effective population size (*N*_e_) from genetic data. Mol. Ecol. Resour..

[69] Wang J. (2011). COANCESTRY: A program for simulating, estimating and analysing relatedness and inbreeding coefficients. Mol. Ecol. Resour..

[70] Peakall R., Smouse P.E. (2012). GenAlEx 6.5: Genetic Analysis in Excel. Population Genetic Software for Teaching and Research—An Update. Bioinformatics.

[71] Goudet J.F. (1995). FSTAT (version 1.2): A computer program to calculate F-statistics. J. Hered..

[72] Excoffier L., Lischer H.E. (2010). Arlequin suite ver 3.5: A new series of programs to perform population genetics analyses under Linux and Windows. Mol. Ecol. Resour..

[73] Piry S., Luikart G., Cornuet J.M. (1999). BOTTLENECK: A computer program for detecting recent reductions in the effective population size using allele frequency data. J. Hered..

[74] Baldwin R.A. (2009). Use of maximum entropy modeling in wildlife research. Entropy.

[75] Araújo M.B., New M. (2007). Ensemble forecasting of species distributions. Trends Ecol. Evol..

[76] Marmion M., Parviainen M., Luoto M., Heikkinen R.K., Thuiller W. (2009). Evaluation of consensus methods in predictive species distribution modelling. Divers. Distrib..

[77] Li Y., Li M., Li C., Liu Z. (2020). Optimized maxent model predictions of climate change impacts on the suitable distribution of *Cunninghamia lanceolata* in China. Forests.

[78] Tamura K., Stecher G., Kumar S. (2021). MEGA11: Molecular evolutionary genetics analysis version 11. Mol. Biol. Evol..

[79] Rozas J., Ferrer-Mata A., Sánchez-DelBarrio J.C., Guirao-Rico S., Librado P., Ramos-Onsins S.E., Sánchez-Gracia A. (2017). DnaSP 6: DNA sequence polymorphism analysis of large data sets. Mol. Biol. Evol..

[80] Clement M., Snell Q., Walker P., Posada D., Crandall K. TCS: Estimating gene genealogies. Proceedings of the 16th International Parallel and Distributed Processing Symposium.

[81] Weir B.S., Cockerhamm C.C. (1984). Estimating *F*-statistics for the analysis of population structure. Evolution.

[82] Excoffier L., Smouse P.E., Quattro J.M. (1992). Analysis of molecular variance inferred from metric distances among DNA haplotypes: Application to human mitochondrial DNA restriction data. Genetics.

[83] Tajima F. (1989). Statistical method for testing the neutral mutation hypothesis by DNA polymorphism. J. Genet..

[84] Fu Y.X., Li W.H. (1993). Statistical tests of neutrality of mutations. J. Genet..

[85] Fu Y.X. (1997). Statistical tests of neutrality of mutations against population growth, hitchhiking and background selection. J. Genet..

[86] Ramos-Onsins S., Rozas J. (2002). Statistical properties of new neutrality tests against population growth. Mol. Biol. Evol..

[87] Rogers A., Harpending H. (1992). Population growth makes waves in the distribution of pairwise genetic differences. Mol. Biol. Evol..

[88] Wakeley J., Hey J. (1997). Estimating ancestral population parameters. J. Genet..

[89] Heled J., Drummond A.J. (2008). Bayesian inference of population size history from multiple loci. BMC Evol. Biol..

[90] Bouckaert R., Vaughan T.G., Barido-Sottani J., Duchne S., Fourment M., Gavryushkina A., Heled J., Jones G., Kühnert D., De Maio N. (2019). BEAST 2.5: An advanced software platform for Bayesian evolutionary analysis. PLoS Comput. Biol..

[91] Guo S.W., Thompson E.A. (1992). Performing the exact test of Hardy-Weinberg proportion for multiple alleles. Biometrics.

[92] Raymond M., Rousset F. (1995). An exact test for population differentiation. Evolution.

[93] R Core Team (2022). R: A Language and Environment for Statistical Computing.

[94] Welch B.L. (1947). The generalization of student’s’ problem when several different population variances are involved. Biometrika.

[95] Van O.C., Hutchinson W.F., Wills D.P., Shipley P. (2004). MICRO-CHECKER: Software for identifying and correcting genotyping errors in microsatellite data. Mol. Ecol. Notes.

[96] Park S.D.E. (2001). The Excel Microsatellite Toolkit (Version 3.1).

[97] Lynch M., Ritland K. (1999). Estimation of pairwise relatedness with molecular markers. J. Genet..

[98] Præstgaard J.T. (1995). Permutation and bootstrap Kolmogorov-Smirnov tests for the equality of two distributions. Scand. J. Stat..

[99] Chapuis M.P., Estoup A. (2007). Microsatellite null alleles and estimation of population differentiation. Mol. Biol. Evol..

[100] Nei M. (1972). Genetic distance between populations. Am. Nat..

[101] Jombart T. (2008). adegenet: A R package for the multivariate analysis of genetic markers. J. Bioinform..

[102] Pritchard J.K., Stephens M., Donnelly P. (2000). Inference of population structure using multilocus genotype data. J. Genet..

[103] Earl D.A. (2012). Structure harvester: A website and program for visualizing STRUCTURE output and implementing the Evanno method. Conserv. Genet. Resour..

