# Supplementary material for: Environmental and Socio–Cultural Factors Impacting the Unique Gene Pool Pattern of Mae Hong-Son Chicken
Source: Animals (Basel). 2023 Jun 10;13(12):1949. doi: 10.3390/ani13121949 (PMC10295432; doi:10.3390/ani13121949)
Supplement: Supplementary file 1 [file animals-13-01949-s001.zip › animals-2290853-supplementary_proof.pdf]

Supplementary Material

1 Supplementary Data

1.1 Supplementary Data S1

Social and cultural history

Mae Hong Son chickens, commonly known as Kai Tai or Kai Jae, are Thai indigenous chickens that are thought to have developed from red junglefowl. These chickens were specifically bred and trained as Kai Tor Kai Tang (decoy) to lure red junglefowl from the wild. Mae Hong Son chickens have played a significant socio-cultural role over the last 200 to 300 years, as evidenced from traditional offerings to spirits (such as weir ghosts, field ghosts or rice paddy ghosts) by the Pgakenyor, Hmong or Tai Yai communities. Mae Hong Son chickens remain closely intertwined with the lives of the local people through Tai proverbs as well as well-known local dishes, Yum Jin Kai (spicy chicken salad) and Oop Kai Tai (chicken curry). According to the *Alphabet Legend* of the Pgakenyor community in Mae Hong Son over the last 300 years, the chicken consumed more alphabets than any other animal (alphabet legends are those which recount the origin, or cause of lack therein, of alphabets in particular groups of people), and therefore, Mae Hong Son chicken bones were historically used by the Pgakenyor community for divination. A wooden stick with a pointed end was inserted into the hole in a chicken leg bone and the direction of the stick was then interpreted. This belief and its corresponding ritual are recorded in an ancient Lanna literary work, *Chao Suwat Nang Buakham*. The *Handbook on the Characteristics of Chicken* also details the good and bad characteristics of *Kai Tor Kai Tang* (native chicken breed name) as believed by the Tai Yai community in Mae Hong Son. Mae Hong Son chickens have been inextricably linked with the lives of Mae Hong Son residents for more than 300 years, as evidenced by Tai proverbs and well-known local dishes.

1.2 Materials and Methods

Species distribution modeling

We selected the logistic output with suitability data ranging from 0 to 1, representing the occurrence probability of target species, with default settings for convergence threshold and maximum number of iterations (500) [1]. Regularization data were selected automatically by MaxEnt software to

reduce model over-fitting [2]. Selection of the optimal MaxEnt model was made using the 10th percentile presence probability and the 10-fold cross-validation method to generate a binary map. Response curves of the predictor variables were developed and Jackknife importance was tested in the final optimal model [3]. To decrease predictive uncertainty, we used the ensemble forecasting approach following Araújo and New [4] and applied the basic mathematical function of mean ensembles to calculate the final logistic outputs [5]. Model validation and predictive performance were used to evaluate the area under the curve (AUC) of receiver operating characteristics [6]. AUC data were threshold-independent measures of model accuracy that illustrated the model's discrimination ability [6], where an AUC value of 0 indicated a model with no better discrimination than chance, an AUC value of 1 indicated perfect discrimination, and an AUC value greater than 0.8 indicated excellent discrimination. The averaged prediction maps depict species presence probability with continuous values ranging from 0–1. This presence probability was classified into five equal-sized categorical classes following Li et al. [7]: SC-5 = very high suitability ($p > 0.8$), SC-4 = high ($0.6 > p \leqslant 0.8$), SC-3 = moderate ($0.4 > p \leqslant 0.6$), SC-2 = least suitability ($0.2 \geqslant p \leqslant 0.4$) and SC-1 = no suitability ($p < 0.2$). Finally, the area under each of the classified categories was calculated using map algebra in ArcGIS.

Mitochondrial D-loop sequence analysis

Multiple sequence alignments were performed for 50 sequences in the mt D-loop dataset. The sequences were aligned using the default parameters of the Molecular Evolutionary Genetics Analysis 11 (MEGA11) software [8]. All unalignable and any sites containing gaps were carefully removed and trimmed from the datasets. Ambiguous sites behind the primer sequences were trimmed from the fragments. Estimates of the haplotype (h) and nucleotide (π) diversity, number of haplotypes (H), the estimator theta (S), overall haplotype and average number of nucleotide differences (k) were calculated using the mt D-loop sequences using DnaSP version 6.12.03 [9]. A statistical parsimony network of the consensus sequences was constructed using the Templeton, Crandall and Sing (TCS) algorithm in PopART version 1.7 to examine the haplotype grouping and population dynamics [10]. The genetic differentiation coefficient (G_{ST}), Wright's F-statistic for subpopulations within the total population (F_{ST}), and the Φ_{ST} values from the sequence data and haplotype data were estimated using Arlequin version 3.5.2.2 [11]. The F_{ST} and Φ_{ST} values were calculated by analyzing 1,000 permutations of haplotypes between the populations [12]. The F_{ST} statistic relies on the differences in haplotype frequencies, whereas Φ_{ST} considers the relationship between the haplotypes based on molecular genetic

distance [13]. The average number of nucleotide substitutions for each site between populations (D_{xy}) and the net nucleotide substitutions of each site between populations (D_a) were analyzed using DnaSP version 6.12.03. A statistical parsimony network of the consensus sequences was constructed using the Templeton, Crandall and Sing (TCS) algorithm in PopART version 1.7 to examine haplotype grouping and population dynamics [10]. The demographic history of the mt D-loop sequence dataset was then determined using the statistical test of neutrality. Tajima's D [14], Fu and Li's D^* and F^* tests [15], Fu's Fs [16], the Ewens-Watterson test and Chakraborty's test were performed using Arlequin version 3.5.2.2 [13]. Ramos-Onsins and Rozas's R_2, which has greater statistical power for small sample sizes, was calculated using DnaSP version 6.12.03 [17]. The significance of the differences among these statistical test values was determined using 10,000 coalescent simulations in accordance with the recommended software parameters. The mismatch distribution approach was used to test for genetic signatures of historical population expansion within Mae Hong Son chickens populations. In this approach, an observed frequency distribution of pairwise nucleotide differences among individuals with expected distributions from an expanding population (small raggedness index) or a stationary population (large raggedness index) was obtained [18,19]. These models were applied to estimate the parameters of population expansion with a generalized least-squares approach and to compute their confidence intervals by bootstrapping (10,000 replicates) using DnaSP version 6.12.03. Bayesian coalescent-based methods were then performed to evaluate historical demographic fluctuations using the Extended Bayesian Skyline Plot (EBSP) in BEAUTi version 2.0.2 (part of the BEAST version 2.0.2 package) [20,21] by applying the Hasegawa–Kishono–Yano model, strict clock model and coalescent Bayesian skyline model with a gamma distribution prior. For the mean substitution rate, the prior was set as a log-normal distribution with a mean of 3.6%/million years and a standard deviation of 0.6%/million years to match the rate estimated from fossil data [22]. The EBSP fitted different demographic scenarios by allowing changes in population size over time. The software package Tracer was applied to assess burn-in and the effective sample sizes (ESS) of the parameters.

Analysis of genetic diversity and population structure among three populations of Mae Hong Son chicken using microsatellite DNA markers

The microsatellite DNA analysis followed the same procedure outlined in our previous animal research projects [23,24]. The allelic frequency, number of alleles (N_a), number of effective alleles (N_{ea}), observed heterozygosity (H_o), expected heterozygosity (H_e), linkage equilibrium and Wright's F-statistic for subpopulations within the total population (F_{ST}) were calculated using Arlequin version

3.5.2.2 [11]. Due to limited population sizes, deviations from the Hardy-Weinberg equilibrium were evaluated at each locus by the Markov chain Monte Carlo (MCMC) approximation of Fisher's exact test using the "genepop" function in the package "stats" of R version 4.1.2 [25–27]. Welch's t-test (unequal variance between samples) identified any significant differences between H_o and H_e using the "t.test" function in "stats" of R version 4.1.2 [27,28]. To test for equal variances between the H_o and H_e, Bartlett's homogeneity of variance test was performed using the "bartlett.test" function in "stats" of R version 4.1.2 [27]. Allelic richness (*AR*) was calculated by FSTAT version 2.9.4 [29]. MicroChecker version 2.2.3 identified the null allelic markers [30]. The polymorphic information content (*PIC*) was estimated using the Excel Microsatellite Toolkit [31] and calculated for each locus. Shannon's information index (*I*) and the fixation index (*F*) were calculated for each locus of the population using GenAlEx version 6.5 [32].

To consider the possibility of sibling-sibling or parent-offspring pairs in the captive population, we determined whether the Mae Hong Son chickens were more related than random unrelated individuals. Relatedness values (*r*) were calculated for all female-female, male-male, and male-female pairs. Mean pairwise *r* values based on population allelic frequencies in captivity were calculated using GenAlEx version 6.5 [32]. Individual and overall F_{IS} with 95% confidence intervals were calculated using the LynchRt estimator [33], as implemented in the program COANCESTRY [34]. Examinations of *r* values and F_{IS} were conducted assuming that the averages did not differ significantly from random assortments of unrelated individuals. Distributions of pairwise *r* values and F_{IS} between all pairs from the sampled captivities were compared by a bootstrap version of the Kolmogorov-Smirnov test to provide relationships [35], using the "ks.test" function in the package stats of R version 4.1.2 [27]. Pairwise genetic distances among populations were calculated based on the infinite allele model (IAM) using F_{ST} with corrected p-values in Arlequin version 3.5.2.2. and the stepwise mutation model (SMM) using R_{ST} in FSTAT version 2.9.3 [29]. Considering the possible influences of null alleles on genetic differentiation estimates, the data were subjected to ENA (excluding null alleles) correction using the Free^{NA} program [36] to obtain pairwise F_{ST} ^{ENA} values. To elucidate the group structure, an analysis of molecular variance (AMOVA) was performed using Arlequin 3.5.2.2. Unlike F_{ST}, this algorithm identifies subgroup hierarchical structure and does not require a priori assumption of the Hardy-Weinberg equilibrium. Nei's genetic distances between groups were then examined using GenAlEx version 6.5 [37]. The heterozygosity excess and shift in allelic frequency distributions in genetically bottlenecked populations were tested using Bottleneck version 1.2.02 [38]. The Wilcoxon signed-rank test was performed using a two-phased mutation model (TPM) and SMM to obtain probabilities for

excess heterozygosity due to the small sample sizes of loci. The TPM was performed with 95% single-step and 5% multi-step mutations, with variance among multiple steps set at 12. However, the Wilcoxon signed-rank test only detects relatively short-term bottleneck events. To test for relatively long-term bottleneck events, the *M* ratio test [39] was performed using Arlequin version 3.5.2.2. The *M* ratio is the mean number of alleles in a population divided by the allelic size range and indicates reductions in both recent and historical population sizes.

Principal coordinate analysis (PCoA) was performed using GenAlEx version 6.5 [32] to assess the overall relationship among individuals in the population. Analysis of principal components (DAPC) was performed using the package ADEGENET 2.0 [40] in R 4.1.2 [27]. DAPC makes no assumptions about population models [40]. It defines synthetic variables where genetic variation is maximized between clusters of individuals (*K*) and minimized within clusters. The model-based clustering method implemented in STRUCTURE version 2.3.4 was used to determine population structure [41]. The run length was set to 100,000 MCMC replicates after a burn-in period of 100,000 generations using correlated allelic frequencies under a straight admixture model. The number of clusters (*K*) varied from 1 to 25, with 25 replicates for each value of *K*. The most probable number of bunches was dictated by plotting the log-likelihood of the information (ln Pr ($X|K$)) [41] over the scope of tested *K* esteems before choosing the *K* esteem value at which ln Pr ($X|K$) settled. The ΔK strategy was also applied using Structure Harvester [42].

2 Supplementary Figures and Tables

2.1 Supplementary Figures

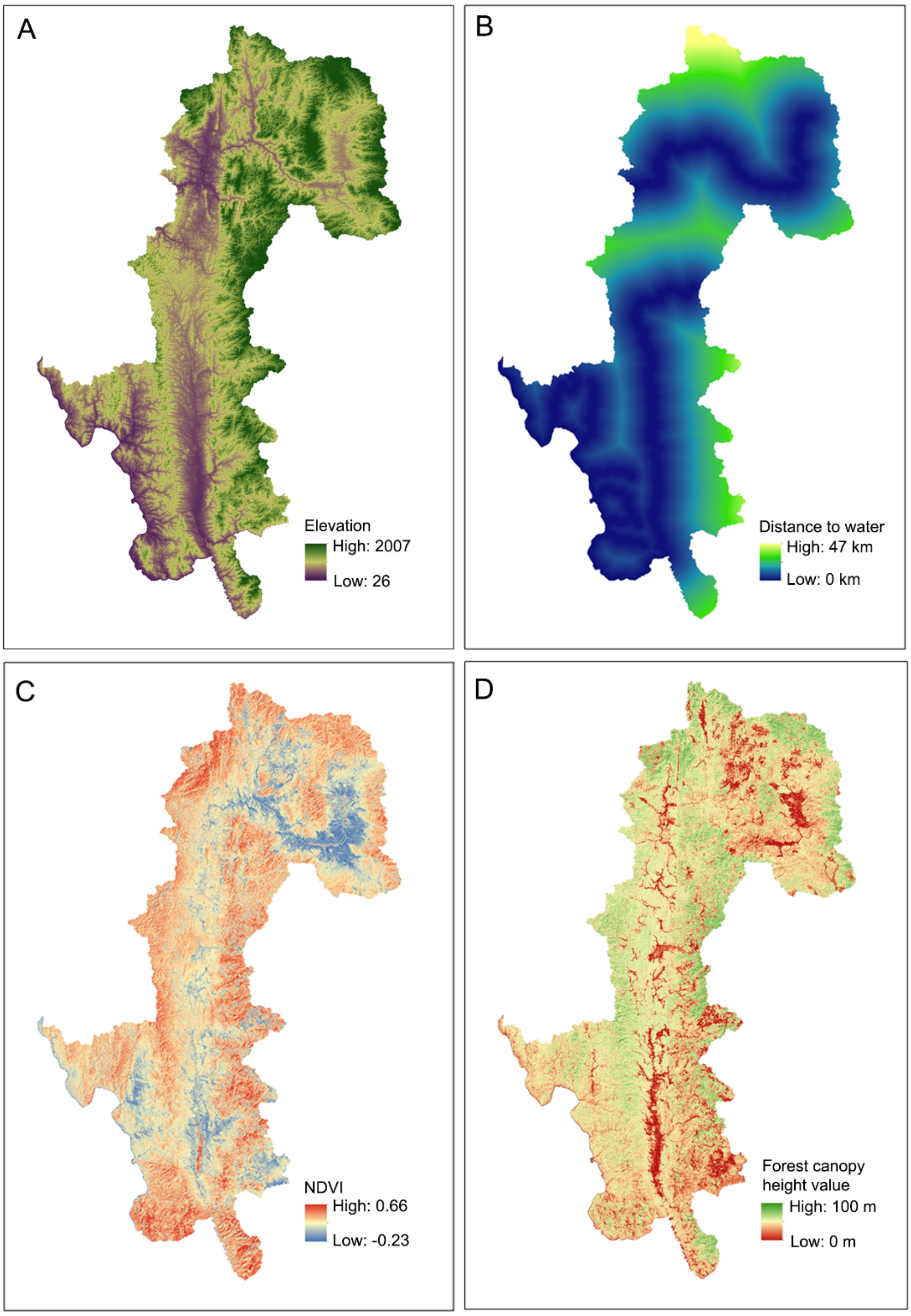

Figure S1 Environmental variables used to assess the species distribution model of Mae Hong Son chicken: (A) Elevation, (B) Distance to water, (C) Normalized difference vegetation index (NDVI) and (D) Forest canopy height.

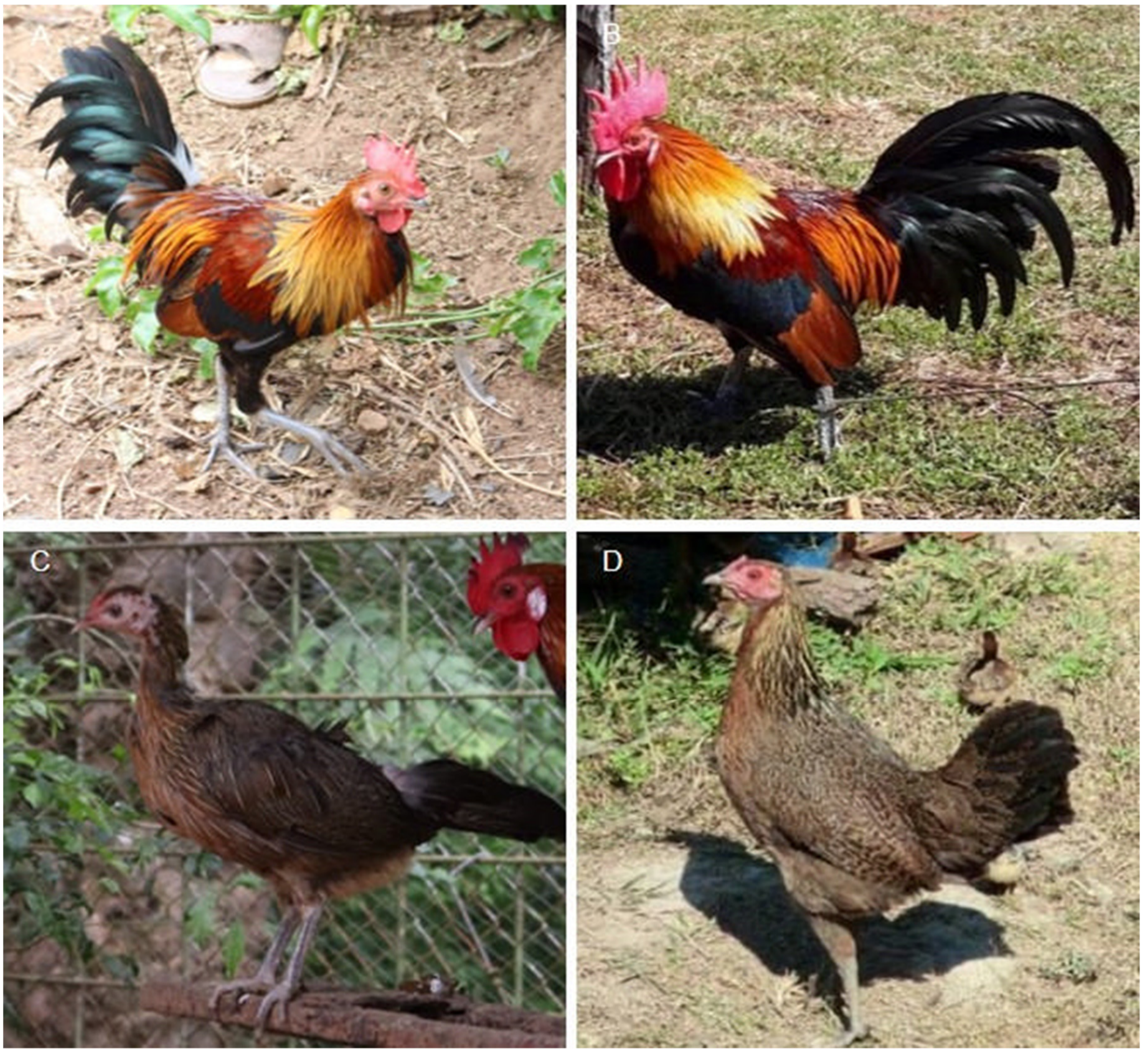

Figure S2 Phenotypic characteristics of male (A–B) and female (C–D) of Mae Hong Son chicken**.**

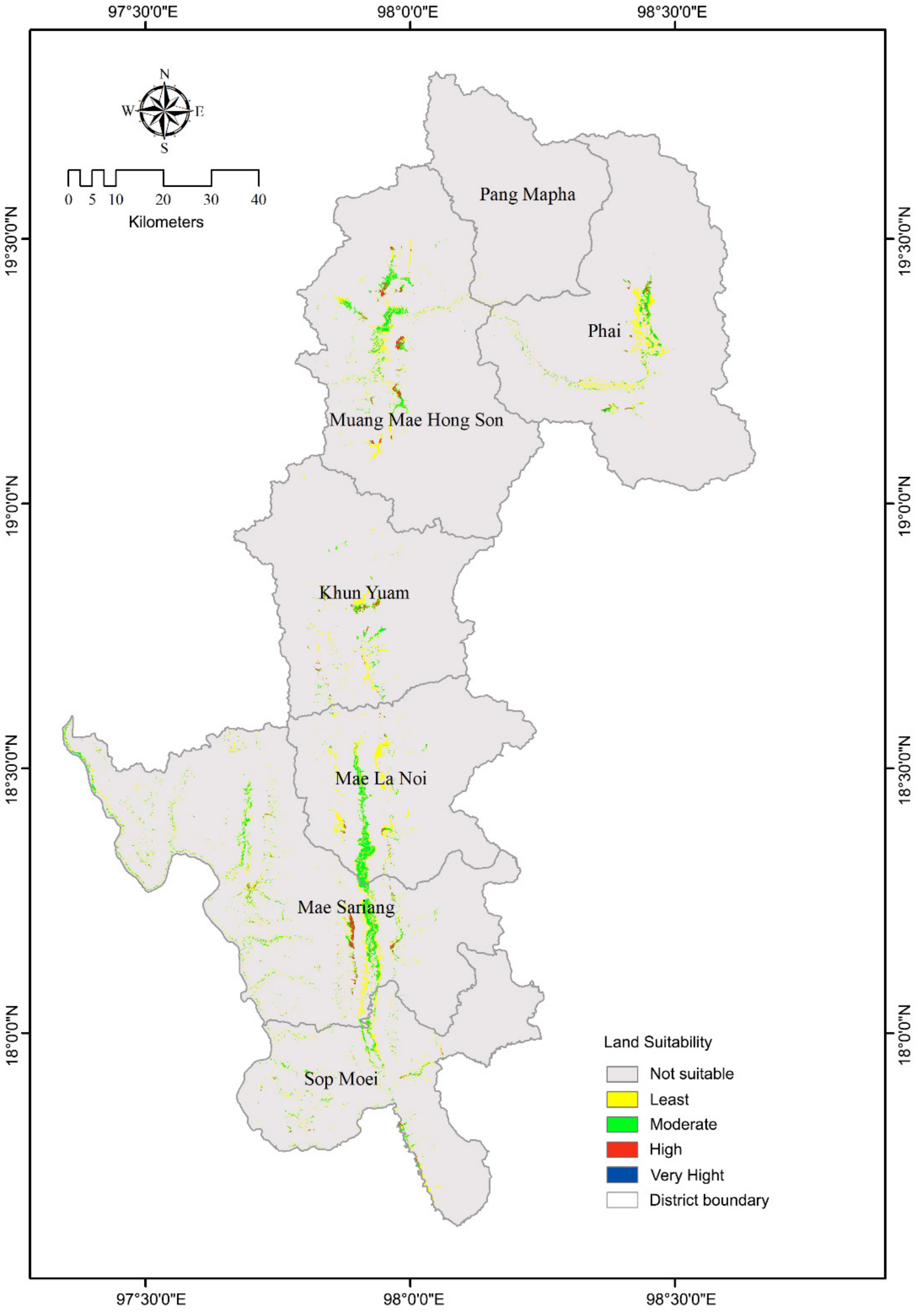

Figure S3. Potential land suitability modeling of Mae Hong Son chickens in Thailand.

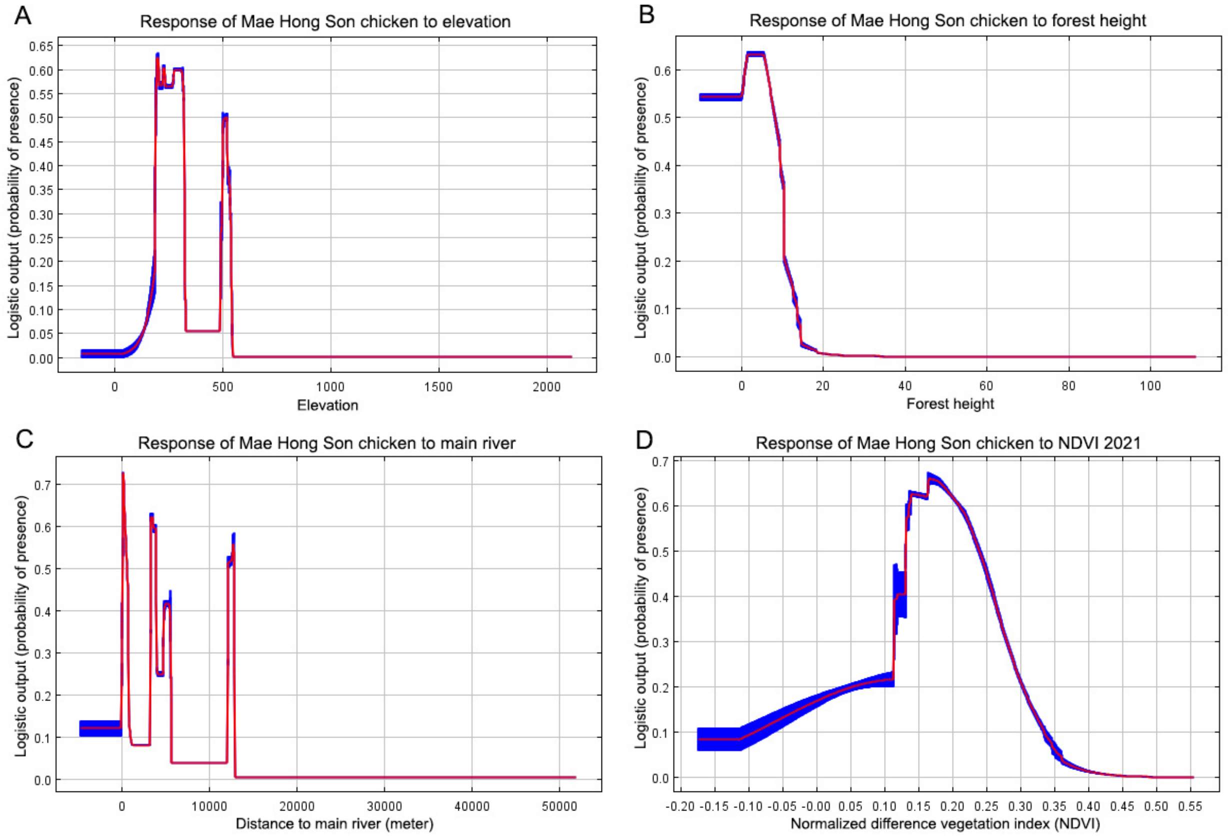

Figure S4. MaxEnt model response area curves of the predictor variables influencing wild chicken distribution. (A) Elevation, (B) Forest canopy height, (C) Distance to main river and (D) Normalized difference vegetation index (NDVI).

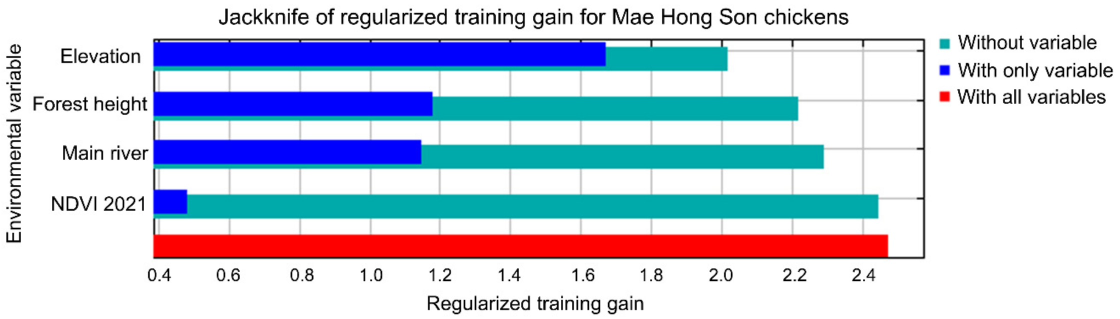

Figure S5. The Jackknife method used in the maximum entropy modeling and environmental factors affecting the potential distribution of Mae Hong Son chickens.

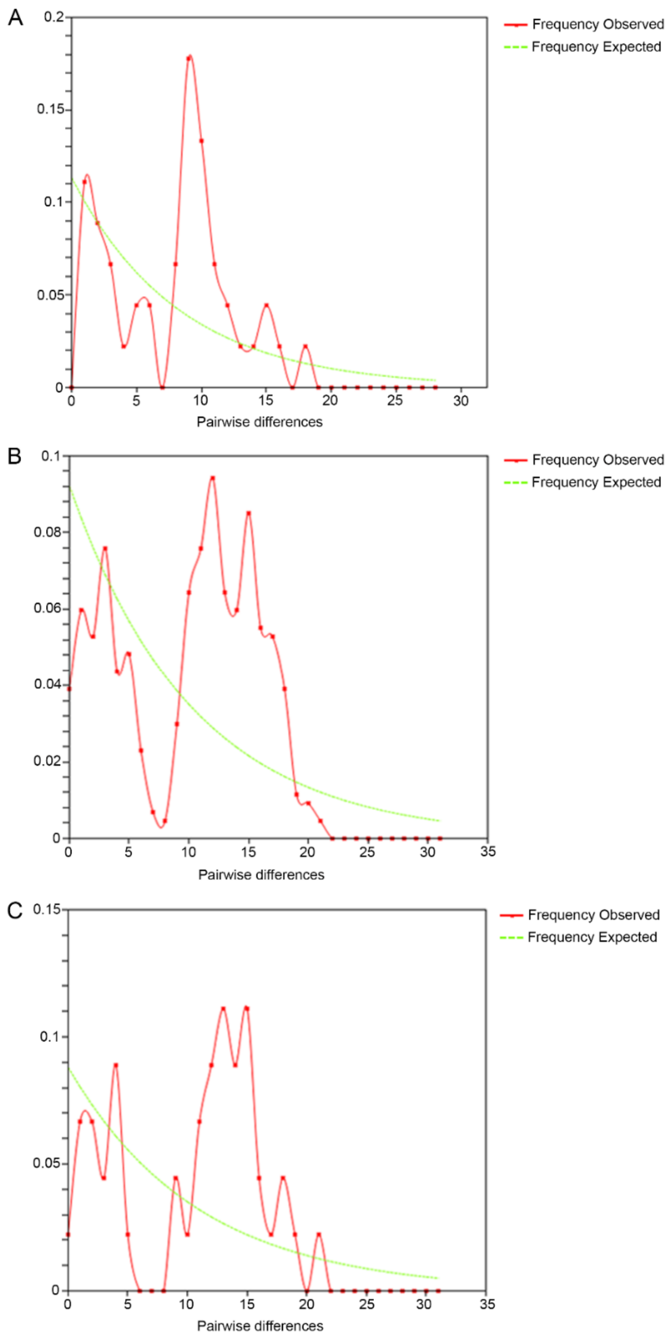

Figure S6. Mismatch Distributions Analysis of Mae Hong Son chickens derived from (A) the Mae Hong Son farmer community, Mae Hong Son, Thailand (MHSF), (B) Mae Hong Son Livestock Research and Breeding Center, Mae Hong Son, Thailand (MLRBC), or (C) Chiang Mai Livestock Research and Breeding Center, Chiang Mai, Thailand (CLRBC). The x- and y-axes represent the number of pairwise differences (mismatches) and the frequency of these differences, respectively. The observed mismatch distribution (red line) is compared to the expected distribution (green line) for a stable population.

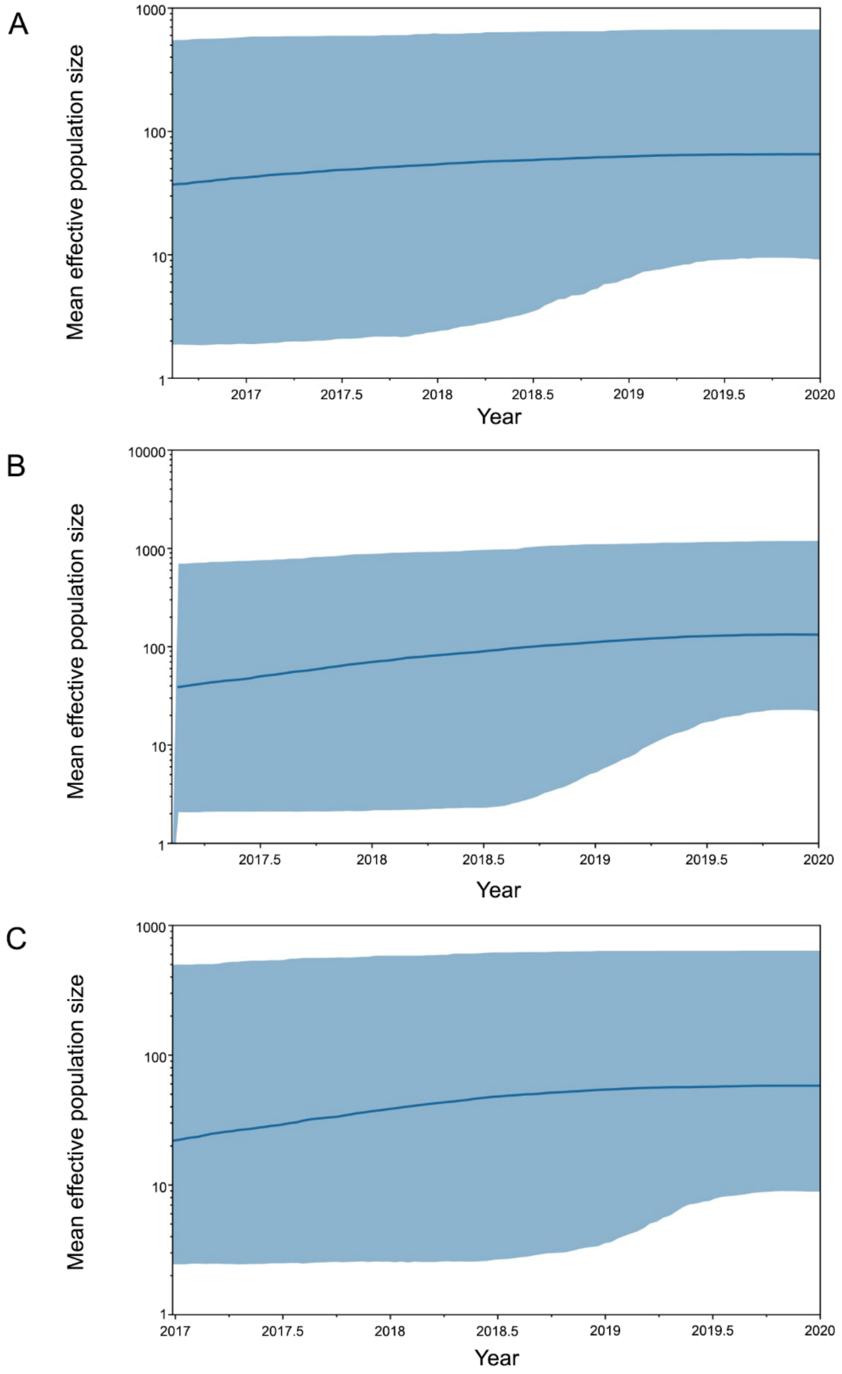

Figure S7. Output of the Coalescent Bayesian Skyline analysis. The black line represents the median estimated effective population size. Blue areas represent the upper and lower bounds. The x-and y-axes represent the time in years and a log scale, respectively. Mae Hong Son chickens derived from (A) the Mae Hong Son farmer community, Mae Hong Son, Thailand (MHSF), (B) Mae Hong Son Livestock Research and Breeding Center, Mae Hong Son, Thailand (MLRBC), and (C) Chiang Mai Livestock Research and Breeding Center, Chiang Mai, Thailand (CLRBC).

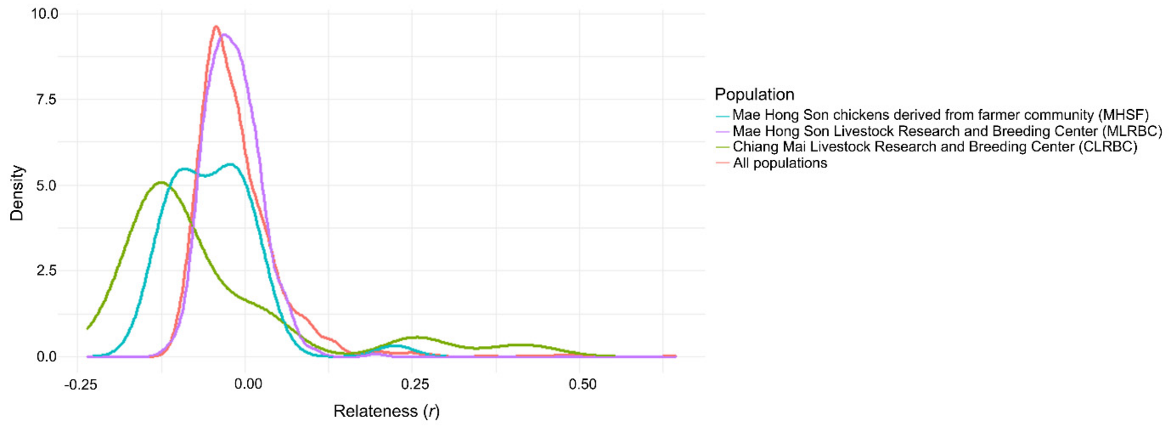

Figure S8. Observed distribution of relatedness (r) in Mae Hong Son chickens (n = 50) (*Gallus gallus*, Linnaeus, 1758) [43], plotted against expected distributions.

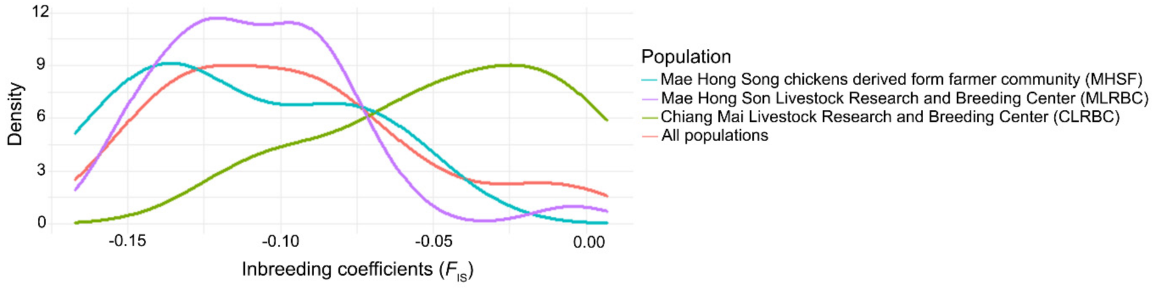

Figure S9. Observed distribution of inbreeding coefficients (F_{IS}) in Mae Hong Son chickens (n = 50) (*Gallus gallus*, Linnaeus, 1758) [43], plotted against expected distributions.

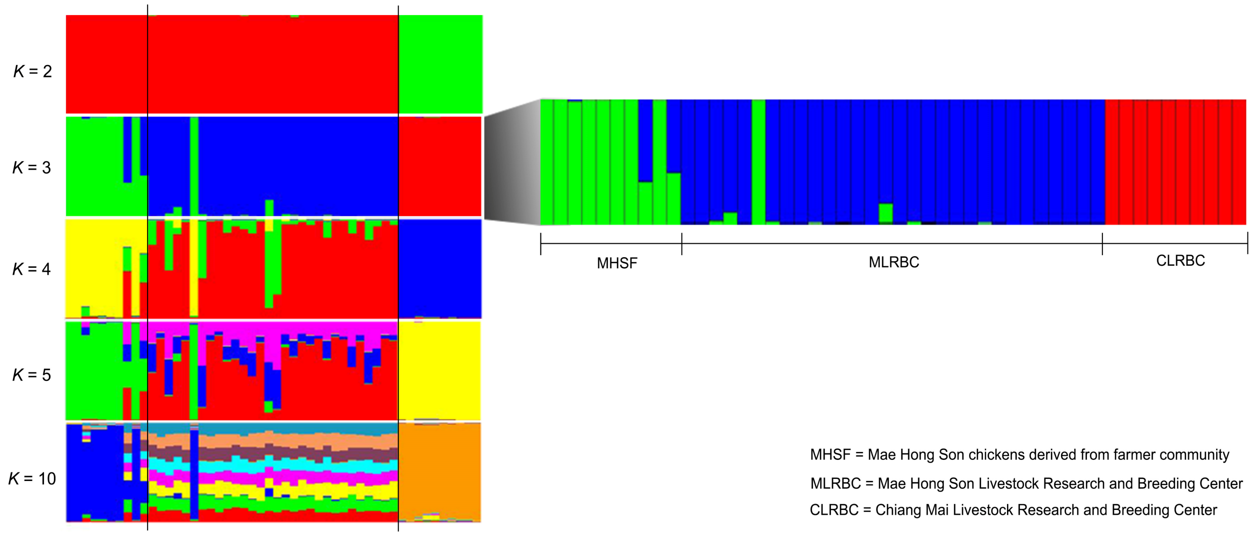

Figure S10. Population structure of Mae Hong Son chickens (n = 50) (*Gallus gallus*, Linnaeus, 1758) [43]). Each vertical bar on the x-axis represents an individual chicken; the y-axis represents the proportion of membership (posterior probability) in each genetic cluster. Red junglefowl are superimposed on the plot with black vertical lines indicating the boundaries. Detailed information on each domestic chicken is presented in Table S1.

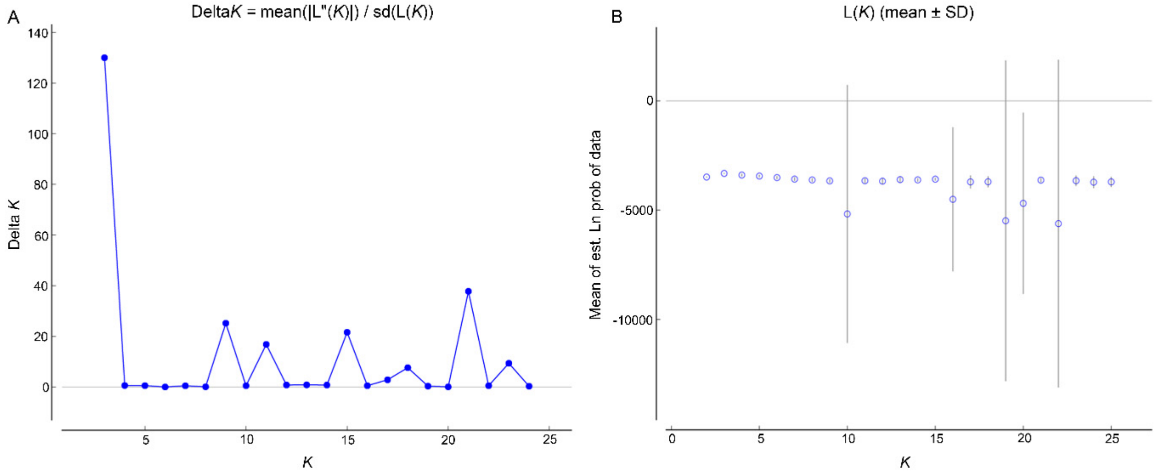

Figure S11. Different population structure patterns of Mae Hong Son chickens (n = 50) (*Gallus gallus*, Linnaeus, 1758) [43] generated by the model-based Bayesian clustering algorithms implemented in STRUCTURE. (A) Plot of Evanno's Δ*K* and (B) Plot of ln P(*K*).

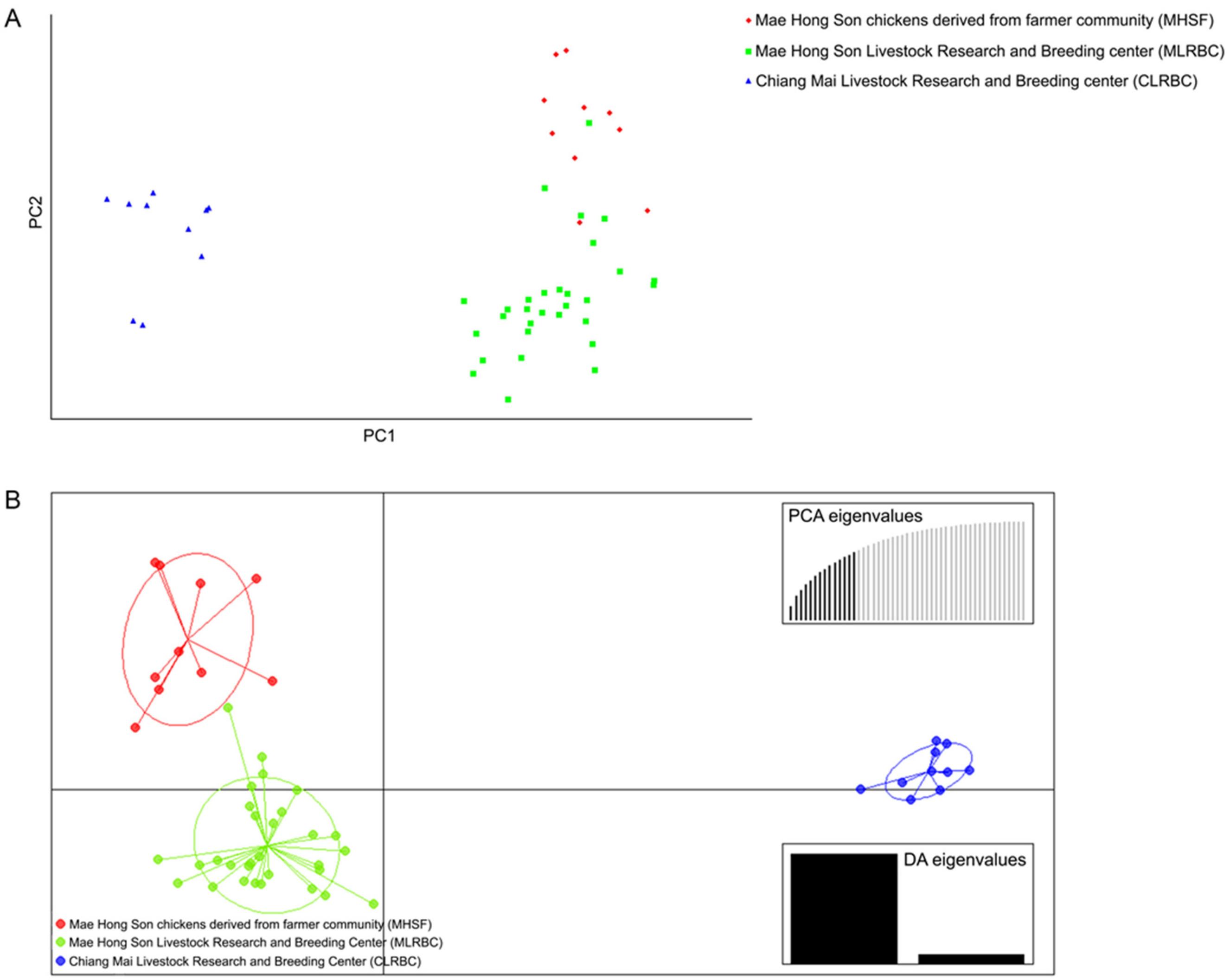

Figure S12. (A) Principal component analysis of Mae Hong Son chickens (*Gallus gallus*, Linnaeus, 1758) [43] and (B) Results of Discriminant Analysis of Principal Components (DAPC) of three indigenous chicken populations derived from the Mae Hong Son farmer community, Mae Hong Son, Thailand (MHSF), Mae Hong Son Livestock Research and Breeding Center, Mae Hong Son, Thailand (MLRBC), and Chiang Mai Livestock Research and Breeding Center, Chiang Mai, Thailand (CLRBC). Scatter plots based on the DAPC output for three assigned genetic clusters are indicated by different colors. Dots represent different individuals. Detailed information on each domestic chicken is presented in Table S1.

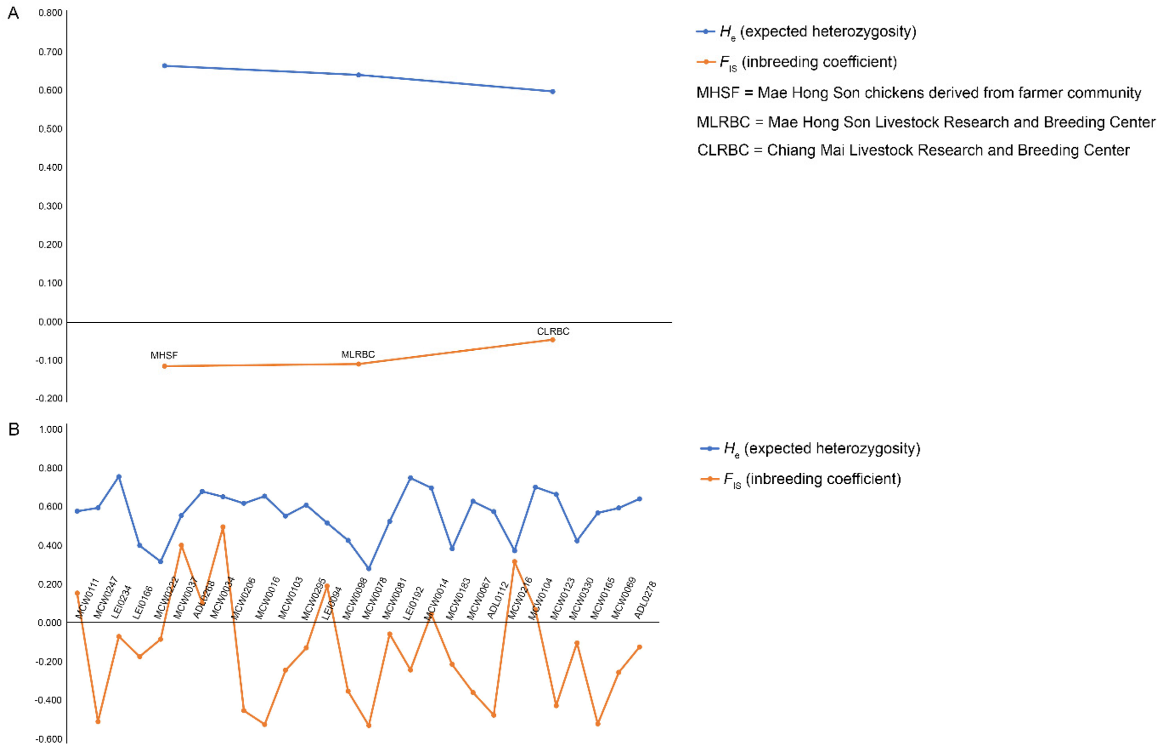

Figure S13. Mapping of expected heterozygosity (H_e) against inbreeding coefficients (F_{IS}) along the length of the physical map. (A) Mae Hong Son chickens populations and (B) microsatellite loci.

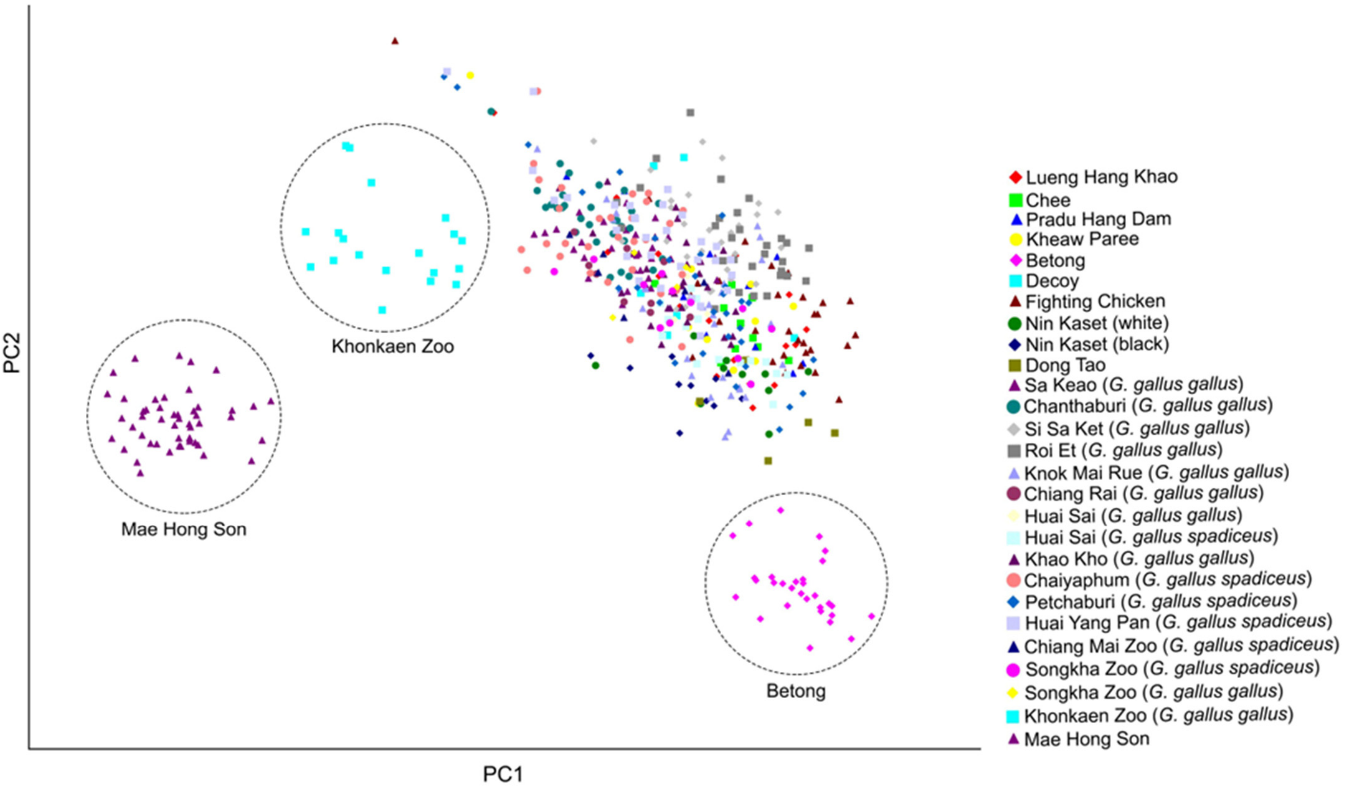

Figure S14. Principal component analysis of Mae Hong Son chickens (*Gallus gallus*, Linnaeus, 1758) [43] derived from the Mae Hong Son farmer community, Mae Hong Son, Thailand (MHSF), Mae Hong Son Livestock Research and Breeding Center, Mae Hong Son, Thailand (MLRBC), and Chiang Mai Livestock Research and Breeding Center, Chiang Mai, Thailand (CLRBC) with red junglefowl and domestic chicken breeds.

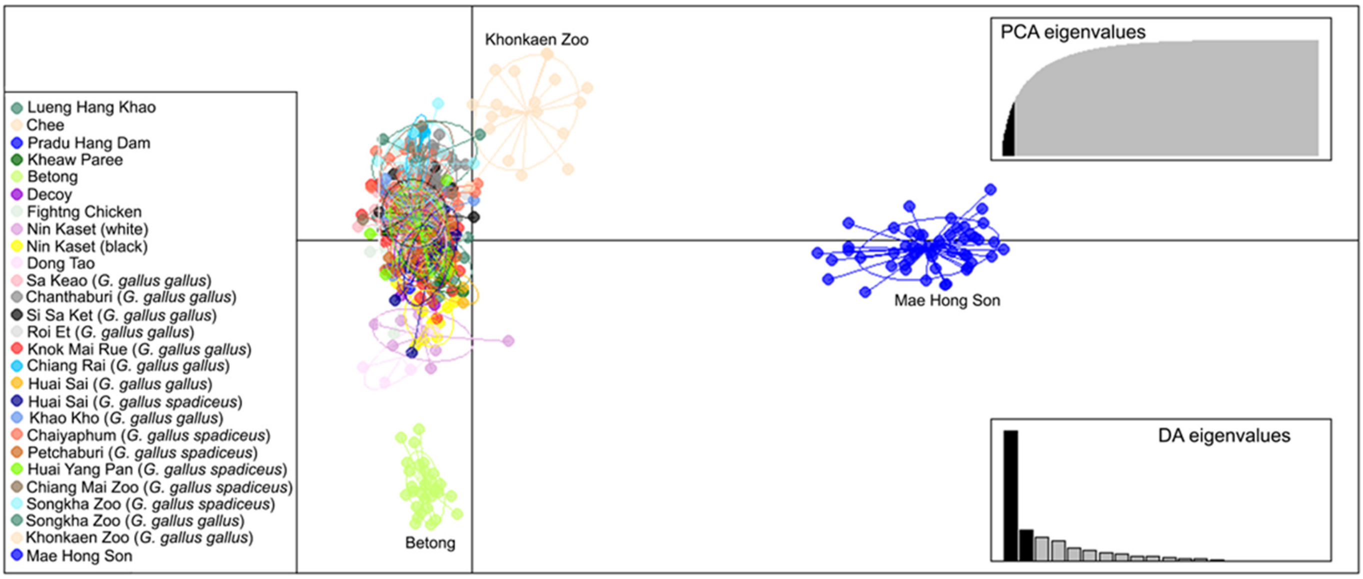

Figure S15. Discriminant Analysis of Principal Components (DAPC) of indigenous chicken populations derived from the Mae Hong Son farmer community, Mae Hong Son, Thailand (MHSF), Mae Hong Son Livestock Research and Breeding Center, Mae Hong Son, Thailand (MLRBC), and Chiang Mai Livestock Research and Breeding Center, Chiang Mai, Thailand (CLRBC) with red junglefowl and domestic breeds. Scatter plots based on the DAPC output for three assigned genetic clusters are indicated by different colors. Dots represent different individuals.

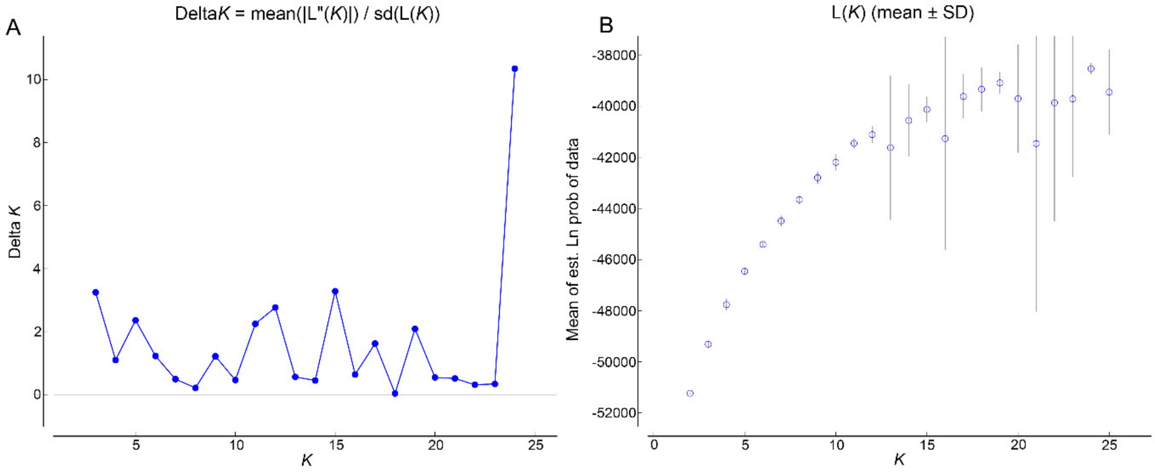

Figure S16. Different population structure patterns of Mae Hong Son chickens (*n* = 50) (*Gallus gallus*, Linnaeus, 1758) [43], red junglefowl and domestic chicken breeds, generated by the model-based Bayesian clustering algorithms implemented in STRUCTURE. (A) Plot of Evanno's Δ*K* and (B) Plot of ln P(*K*).

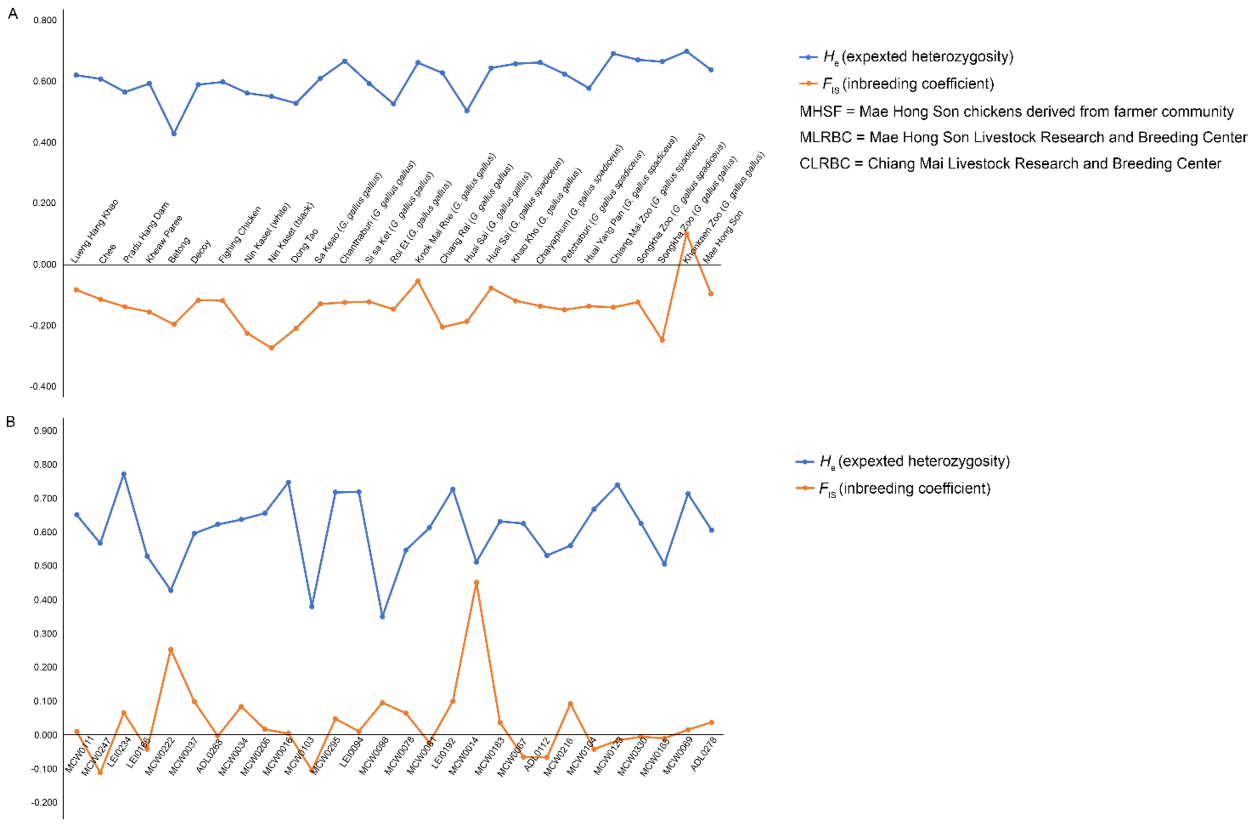

Figure S17. Mapping of expected heterozygosity (H_e) against inbreeding coefficients (F_{IS}) along the length of the physical map. (A) Mae Hong Son chickens breed, red junglefowl and domestic chicken breeds and (B) microsatellite loci.

2.2 Supplementary Tables

Table S1. Detailed information on the specimens from three populations of Mae Hong Son chickens (*Gallus gallus*, Linnaeus, 1758) [43] in Thailand. All sequences were deposited in the DNA Data Bank of Japan (DDBJ).

No.	Abbreviation/Code	Sex	Locality	Mitochondrial D-loop DDBJ accession number	Haplogroup
1	MHS M1F	Male	Mae Hong Son chickens derived from farmer community, MHSF	LC731861	A
2	MHS M2F	Male	Mae Hong Son chickens derived from farmer community, MHSF	LC731862	A
3	MHS FM3F	Female	Mae Hong Son chickens derived from farmer community, MHSF	LC731863	A
4	MHS M4F	Male	Mae Hong Son chickens derived from farmer community, MHSF	LC731864	E
5	MHS FM5F	Female	Mae Hong Son chickens derived from farmer community, MHSF	LC731865	A
6	MHS M6F	Male	Mae Hong Son chickens derived from farmer community, MHSF	LC731866	Unclassified
7	MHS M7F	Male	Mae Hong Son chickens derived from farmer community, MHSF	LC731867	E
8	MHS FM8F	Female	Mae Hong Son chickens derived from farmer community, MHSF	LC731868	A
9	MHS FM9F	Female	Mae Hong Son chickens derived from farmer community, MHSF	LC731869	E
10	MHS FM10F	Female	Mae Hong Son chickens derived from farmer community, MHSF	LC731870	F
11	MHS M1	Male	Mae Hong Son Livestock Research and Breeding Center, MLRBC	LC731886	F
12	MHS M2	Male	Mae Hong Son Livestock Research and Breeding Center, MLRBC	LC731887	E
13	MHS M3	Male	Mae Hong Son Livestock Research and Breeding Center, MLRBC	LC731888	E
14	MHS M4	Male	Mae Hong Son Livestock Research and Breeding Center, MLRBC	LC731889	F
15	MHS M5	Male	Mae Hong Son Livestock Research and Breeding Center, MLRBC	LC731890	F
16	MHS M6	Male	Mae Hong Son Livestock Research and Breeding Center, MLRBC	LC731891	E
17	MHS M7	Male	Mae Hong Son Livestock Research and Breeding Center, MLRBC	LC731892	E
18	MHS M8	Male	Mae Hong Son Livestock Research and Breeding Center, MLRBC	LC731893	F
19	MHS M9	Male	Mae Hong Son Livestock Research and Breeding Center, MLRBC	LC731894	F
20	MHS M10	Male	Mae Hong Son Livestock Research and Breeding Center, MLRBC	LC731895	B
21	MHS M11	Male	Mae Hong Son Livestock Research and Breeding Center, MLRBC	LC731896	F
22	MHS M12	Male	Mae Hong Son Livestock Research and Breeding Center, MLRBC	LC731897	F
23	MHS M13	Male	Mae Hong Son Livestock Research and Breeding Center, MLRBC	LC731898	F
24	MHS M14	Male	Mae Hong Son Livestock Research and Breeding Center, MLRBC	LC731899	F
25	MHS M15	Male	Mae Hong Son Livestock Research and Breeding Center, MLRBC	LC731900	E
26	MHS FM1	Female	Mae Hong Son Livestock Research and Breeding Center, MLRBC	LC731901	E
27	MHS FM2	Female	Mae Hong Son Livestock Research and Breeding Center, MLRBC	LC731902	E
28	MHS FM3	Female	Mae Hong Son Livestock Research and Breeding Center, MLRBC	LC731903	B
29	MHS FM4	Female	Mae Hong Son Livestock Research and Breeding Center, MLRBC	LC731904	F
30	MHS FM5	Female	Mae Hong Son Livestock Research and Breeding Center, MLRBC	LC731905	F

31	MHS FM6	Female	Mae Hong Son Livestock Research and Breeding Center, MLRBC	LC731906	E
32	MHS FM7	Female	Mae Hong Son Livestock Research and Breeding Center, MLRBC	LC731907	B
33	MHS FM8	Female	Mae Hong Son Livestock Research and Breeding Center, MLRBC	LC731908	E
34	MHS FM9	Female	Mae Hong Son Livestock Research and Breeding Center, MLRBC	LC731909	E
35	MHS FM10	Female	Mae Hong Son Livestock Research and Breeding Center, MLRBC	LC731910	B
36	MHS FM11	Female	Mae Hong Son Livestock Research and Breeding Center, MLRBC	LC731911	B
37	MHS FM12	Female	Mae Hong Son Livestock Research and Breeding Center, MLRBC	LC731912	E
38	MHS FM13	Female	Mae Hong Son Livestock Research and Breeding Center, MLRBC	LC731913	F
39	MHS FM14	Female	Mae Hong Son Livestock Research and Breeding Center, MLRBC	LC731914	E
40	MHS FM15	Female	Mae Hong Son Livestock Research and Breeding Center, MLRBC	LC731915	F
41	MHS1	Female	Chiang Mai Livestock Research and Breeding Center, CLRBC	LC731871	B
42	MHS2	Female	Chiang Mai Livestock Research and Breeding Center, CLRBC	LC731872	B
43	MHS3	Female	Chiang Mai Livestock Research and Breeding Center, CLRBC	LC731873	E
44	MHS4	Female	Chiang Mai Livestock Research and Breeding Center, CLRBC	LC731874	F
45	MHS5	Female	Chiang Mai Livestock Research and Breeding Center, CLRBC	LC731875	F
46	MHS6	Female	Chiang Mai Livestock Research and Breeding Center, CLRBC	LC731876	E
47	MHS7	Female	Chiang Mai Livestock Research and Breeding Center, CLRBC	LC731877	E
48	MHS8	Female	Chiang Mai Livestock Research and Breeding Center, CLRBC	LC731878	E
49	MHS9	Female	Chiang Mai Livestock Research and Breeding Center, CLRBC	LC731879	E
50	MHS10	Female	Chiang Mai Livestock Research and Breeding Center, CLRBC	LC731880	B

Table S2. Microsatellite primers and sequences of Mae Hong Son chickens specimens.

Primer	Chromosome	Annealing temperate (°C)	Fluorescence	Primer sequence 5′ to 3′		Size (bp)
				Forward	Reverse	
ADL0268	1	58	Hex	CTCCACCCCTCTCAGAACTA	CAACTTCCCATCTACCTACT	106–127
MCW0111	1	58	Hex	GCTCCATGTGAAGTGGTTTA	GCTCCATGTGAAGTGGTTTA	94–120
MCW0247	1	58	Fam	GTTGTTCAAAAGAAGATGCATG	TTGCATTAACTGGGCACTTTC	208–222
LEI0234	2	58	Hex	ATGCATCAGATTGGTATTCAA	CGTGGCTGTGAACAAATATG	218–373
MCW0034	2	58	Hex	ATCTTGAAACCTCACAAAGC	TCTTCCAACCTATTTTTAGT	211–241
MCW0206	2	58	Fam	CTTGACAGTGATGCATTAAATG	ACATCTAGAATTGACTGTTCAC	217–247
LEI0166	3	58	Hex	CTCCTGCCCTTAGCTACGCA	TATCCCCTGGCTGGGAGTTT	338–365
MCW0222	3	58	Hex	GCAGTTACATTGAAATGATTCC	TTCTCAAAACACCTAGAAGAC	218–230
MCW0016	3	58	Fam	ATGGCGCAGAAGGCAAAGCGATAT	TGGCTTCTGAAGCAGTTGCTATGG	126–177
MCW0037	3	58	Fam	ACCGGTGCCATCAATTACCTATTA	GAAAGCTCACATGACACTGCGAAA	149–154
MCW0103	3	58	Fam	AACTGCGTTGAGAGTGAATGC	TTTCCTAACTGGATGCTTCTG	263–270
MCW0295	4	58	Fam	ATCACTACAGAACACCCTCTC	TATGTATGCACGCAGATATCC	82–111
LEI0094	4	58	Hex	GATCTCACCAGTATGAGCTGC	TCTCACACTGTAACACAGTGC	243–281
MCW0078	5	58	Hex	CCACACGGAGAGGAGAAGGTCT	TAGCATATGAGTGTACTGAGCTTC	144–154
MCW0098	4	58	Hex	GGCTGCTTTGTGCTCTTCTCG	CGATGGTCGTAATTCTCACGT	247–257
MCW0081	5	58	Fam	GTTGCTGAGAGCCTGGTGCAG	CCTGTATGTGGAATTACTTCTC	107–146
LEI0192	6	58	Hex	TGCCAGAGCTTCAGTCTGT	GTCATTACTGTTATGTTTATTGC	251–490
MCW0014	6	58	Hex	TATTGGCTCTAGGAACTGTC	GAAATGAAGGTAAGACTAGC	169–202
MCW0183	7	58	Hex	ATCCCAGTGTCGAGTATCCGA	TGAGATTTACTGGAGCCTGCC	293–361
ADL0278	8	58	Hex	CCAGCAGTCTACCTTCCTAT	TGTCATCCAAGAACAGTGTG	118–136
MCW0067	10	58	Fam	GCACTACTGTGTGCTGCAGTTT	GAGATGTAGTTGCCACATTCCGAC	170–182
ADL0112	10	58	Hex	GGCTTAAGCTGACCCATTAT	ATCTCAAATGTAATGCGTGC	132–142
MCW0216	13	58	Hex	GGGTTTTACAGGATGGGACG	AGTTTCACTCCCAGGGCTCG	135–170
MCW0104	13	58	Hex	TAGCACAACTCAAGCTGTGAG	AGACTTGCACAGCTGTGACC	194–249
MCW0123	14	58	Hex	CCACTAGAAAAGAACATCCTC	GGCTGATGTAAGAAGGGATGA	84–106
MCW0330	17	58	Hex	TGGACCTCATCAGTCTGACAG	AATGTTCTCATAGAGTTCCTGC	259–291
MCW0165	23	58	Hex	CAGACATGCATGCCCAGATGA	GATCCAGTCCTGCAGGCTGC	111–115
MCW0069	26	58	Hex	GCACTCGAGAAAACTTCCTGCG	ATTGCTTCAGCAAGCATGGGAGGA	155–178

HEX = HEX™ Dye Phosphoramidite, FAM = 6-FAM (6-Carboxyfluorescein).

Table S3. Genetic differentiation between the three populations of Mae Hong Son chickens (*Gallus gallus*, Linnaeus, 1758) [43], based on the mitochondrial D-loop sequence. G_{ST}: Genetic differentiation coefficient, F_{ST}: Wright's *F*-statistics for subpopulations within the total population, Φ_{ST}: from sequence and haplotype data, D_{xy}: the average number of nucleotide substitutions per site between populations, and D_a: net nucleotide substitutions per site between populations.

**Population 1**	**Population 2**	F_{ST}	G_{ST}	Φ_{ST}	D_{xy}	D_a
MHSF[1]	MLRBC[2]	0.093*	0.021	0.001	0.010	0.002
MHSF[1]	CLRBC[3]	0.017^{ns}	0.029	0.001	0.009	0.001
MLRBC[2]	CLRBC[3]	0.127**	0.014	0.000	0.009	0.000

* $p < 0.05$, ** $p < 0.01$, ns = not significant.

[1]MHSF = Mae Hong Son chickens derived from farmer community, Mae Hong Son.
[2]MLRBC = Mae Hong Son Livestock Research and Breeding Center, Mae Hong Son.
[3]CLRBC = Chiang Mai Livestock Research and Breeding Center, Chiang Mai.

Table S4. Neutrality tests of mitochondrial D-loop sequence of Mae Hong Son chickens (*Gallus gallus*, Linnaeus, 1758) [43].

Population	Tajima	Fu *D**	Fu *F**	Fu's F_s	Ewens-Watterson test	Chakraborty's test	Ramos-Onsins and Rozas's R_2	Raggedness index
MHSF[1]	-0.121^{ns}	-0.516^{ns}	-0.487^{ns}	-3.126*	N.A.	0.000^{ns}	0.142	0.079^{ns}
MLRBC[2]	-0.105^{ns}	-0.780^{ns}	-0.655^{ns}	-4.789^{ns}	0.997^{ns}	0.039^{ns}	0.113	0.009^{ns}
CLRBC[3]	0.189^{ns}	0.577^{ns}	0.511^{ns}	-0.894^{ns}	1.000^{ns}	0.022^{ns}	0.163	0.067^{ns}
All populations	0.030^{ns}	-0.965^{ns}	-0.767^{ns}	-1.425^{ns}	0.997^{ns}	0.057^{ns}	0.103	0.062^{ns}

*$p<0.05$, ns = not significant.

[1]MHSF = Mae Hong Son chickens derived from farmer community, Mae Hong Son.
[2]MLRBC = Mae Hong Son Livestock Research and Breeding Center, Mae Hong Son.
[3]CLRBC = Chiang Mai Livestock Research and Breeding Center, Chiang Mai.

Table S5. Pairwise differentiation of linkage disequilibrium of Mae Hong Son chickens (*Gallus gallus*, Linnaeus, 1758) [43] derived from Mae Hong Son farmer community, Mae Hong Son, Thailand (MHSF), based on 28 microsatellite loci. Numbers indicate *p*-values with 110 permutations.

Locus	MCW0111	MCW0247	LEI0234	LEI0166	MCW0222	MCW0037	ADL0268	MCW0034	MCW0206	MCW0016	MCW0103	MCW0295	LEI0094	MCW0098	MCW0078	MCW0081	LEI0192	MCW0014	MCW0183	MCW0067	ADL0112	MCW0216	MCW0104	MCW0123	MCW0330	MCW0165	MCW0069	ADL0278
MCW0111	0.000																											
MCW0247	0.247	0.000																										
LEI0234	0.921	0.044	0.000																									
LEI0166	0.067	0.064	0.132	0.000																								
MCW0222	0.029	0.471	0.329	0.379	0.000																							
MCW0037	0.911	0.589	0.616	0.364	0.552	0.000																						
ADL0268	0.118	0.481	0.469	0.132	0.182	0.671	0.000																					
MCW0034	0.054	0.490	0.769	0.801	0.126	0.971	0.383	0.000																				
MCW0206	1.000	1.000	0.471	0.666	0.137	0.765	0.801	0.820	0.000																			
MCW0016	0.055	0.004	0.153	0.128	0.747	0.767	0.246	0.209	1.000	0.000																		
MCW0103	0.163	1.000	0.309	0.660	0.072	1.000	0.104	0.663	0.522	0.811	0.000																	
MCW0295	0.082	0.094	0.594	0.023	0.539	0.048	0.195	0.255	0.759	0.139	1.000	0.000																
LEI0094	0.058	0.244	0.045	0.220	0.465	0.741	0.941	0.493	1.000	0.423	0.465	0.178	0.000															
MCW0098	0.553	0.717	0.156	0.781	0.301	0.289	0.689	0.716	0.546	0.926	0.570	0.300	0.032	0.000														
MCW0078	0.081	1.000	0.607	0.412	0.723	0.100	0.334	0.748	0.720	0.082	0.166	0.269	0.419	0.587	0.000													
MCW0081	0.276	0.900	0.912	1.000	0.452	0.456	0.119	0.876	1.000	1.000	0.597	0.914	0.906	0.041	0.664	0.000												
LEI0192	0.411	0.765	0.267	0.914	0.511	0.502	0.133	0.292	1.000	0.638	0.719	0.510	0.767	0.098	0.892	0.045	0.000											
MCW0014	0.480	1.000	0.185	0.537	0.474	1.000	0.458	0.480	0.418	1.000	1.000	0.470	0.240	0.224	1.000	0.477	0.202	0.000										
MCW0183	0.098	0.299	0.409	0.196	1.000	1.000	0.403	1.000	1.000	0.402	0.402	0.203	0.102	1.000	0.224	1.000	1.000	0.295	0.000									
MCW0067	0.634	0.471	0.349	1.000	0.234	0.208	0.476	0.811	0.103	0.584	0.102	0.866	0.475	0.212	1.000	0.642	0.376	0.776	1.000	0.000								
ADL0112	0.208	0.532	0.129	0.371	0.382	1.000	0.729	0.397	0.669	0.656	0.668	0.387	0.069	0.454	0.418	1.000	0.908	0.069	0.197	1.000	0.000							
MCW0216	0.234	0.886	0.895	1.000	0.901	0.362	0.619	0.476	0.494	0.957	0.472	0.894	0.885	0.524	0.778	0.163	0.471	0.797	1.000	0.750	1.000	0.000						
MCW0104	1.000	0.871	0.826	0.719	0.624	0.174	1.000	1.000	0.874	1.000	1.000	0.633	1.000	1.000	1.000	0.238	1.000	1.000	1.000	1.000	1.000	0.165	0.000					
MCW0123	0.337	1.000	0.163	0.776	0.829	0.297	0.231	0.712	0.551	1.000	0.118	0.830	0.393	0.0.93	0.280	0.114	0.540	1.000	1.000	0.504	1.000	0.026	0.170	0.000				
MCW0330	0.830	0.033	0.076	0.135	1.000	1.000	0.850	1.000	1.000	0.062	0.577	0.219	0.664	1.000	1.000	0.830	0.712	1.000	0.399	1.000	0.670	0.487	1.000	0.572	0.000			
MCW0165	0.125	0.378	0.378	1.000	0.726	0.391	0.656	0.672	0.666	0.382	0.067	0.438	0.411	1.000	0.905	0.073	0.200	1.000	0.020	1.000	1.000	1.000	0.668	0.137	0.735	0.000		
MCW0069	0.137	0.735	0.830	0.421	0.229	0.221	0.459	0.308	0.577	0.867	0.277	0.741	1.000	0.165	0.554	0.057	0.029	0.731	1.000	0.915	0.860	0.071	0.117	0.037	0.275	0.857	0.000	
ADL0278	0.691	0.967	1.000	0.805	0.794	0.243	0.229	0.523	0.166	1.000	1.000	0.789	0.965	0.308	1.000	0.159	1.000	0.961	1.000	1.000	0.784	0.691	0.580	0.100	0.562	0.806	0.110	0.000

Table S6. Pairwise differentiation of linkage disequilibrium of Mae Hong Son chickens (*Gallus gallus*, Linnaeus, 1758) [43] from Mae Hong Son Livestock Research and Breeding Center, Mae Hong Son, Thailand (MLRBC), based on 28 microsatellite loci. Numbers indicate *p*-values with 110 permutations.

Locus	MCW 0111	MCW 0247	LEI02 34	LEI01 66	MCW 0222	MCW 0037	ADL0 268	MCW 0034	MCW 0206	MCW 0016	MCW 0103	MCW 0295	LEI00 94	MCW 0098	MCW 0078	MCW 0081	LEI01 92	MCW 0014	MCW 0183	MCW 0067	ADL0 112	MCW 0216	MCW 0104	MCW 0123	MCW 0330	MCW 0165	MCW 0069	ADL0 278
MCW0111	0.000																											
MCW0247	0.831	0.000																										
LEI0234	0.676	0.765	0.000																									
LEI0166	0.254	0.617	0.527	0.000																								
MCW0222	.0.188	0.658	0.127	0.285	0.000																							
MCW0037	0.918	0.616	0.639	0.718	0.643	0.000																						
ADL0268	0.933	0.210	0.121	0.354	0.185	0.490	0.000																					
MCW0034	0.781	0.296	0.274	0.607	0.289	0.488	0.325	0.000																				
MCW0206	0.952	0.149	0.370	0.523	0.470	0.404	0.351	0.243	0.000																			
MCW0016	0.868	0.249	0.766	0.001	0.205	0.003	0.721	0.726	0.701	0.000																		
MCW0103	0.461	0.611	0.707	0.870	0.380	0.458	0.204	0.851	0.594	0.396	0.000																	
MCW0295	0.103	0.426	0.0313	0.055	0.094	0.635	0.578	0.878	0.703	0.249	0.846	0.000																
LEI0094	0.217	0.973	0.341	0.824	0.545	0.643	0.876	0.446	0.630	0.087	0.442	0.303	0.000															
MCW0098	0.331	0.849	0.241	0.209	0.022	0.105	0.120	0.058	0.045	0.431	0.851	0.099	0.227	0.000														
MCW0078	0.374	0.488	0.083	0.551	0.028	0.285	0.142	0.222	0.814	0.755	1.000	0.885	0.717	1.000	0.000													
MCW0081	0.775	0.010	0.145	0.929	0.252	0.526	0.099	0.055	0.514	0.602	0.485	0.849	0.817	0.515	0.020	0.000												
LEI0192	0.140	0.960	0.179	0.786	0.432	0.665	0.336	0.597	0.835	0.227	0.229	0.241	0.077	0.899	0.434	0.815	0.000											
MCW0014	0.473	0.680	0.085	0.212	0.614	0.658	0.247	0.590	0.916	0.363	0.998	0.216	0.281	0.911	0.090	0.010	0.538	0.000										
MCW0183	0.243	0.919	0.323	0.542	0.892	0.615	0.998	0.740	0.254	0.250	0.483	0.921	0.445	0.645	1.000	0.067	0.577	0.627	0.000									
MCW0067	0.941	0.415	0.882	0.962	0.190	0.254	0.045	0.272	0.621	0.931	0.649	0.933	0.381	0.461	0.356	0.672	0.230	0.593	0.993	0.000								
ADL0112	0.942	0.810	0.022	0.901	0.290	0.167	0.348	0.714	0.751	0.427	0.340	0.689	0.243	0.823	0.226	0.047	0.176	0.490	0.304	0.514	0.000							
MCW0216	0.784	0.303	0.551	0.740	0.293	0.585	0.741	0.194	0.475	0.743	0.048	0.774	0.551	0.511	0.080	0.225	0.597	0.160	0.055	0.787	0.831	0.000						
MCW0104	0.684	0.445	1.000	0.983	0.865	0.837	1.000	0.475	0.994	0.738	0.859	0.483	0.055	0.044	0.969	0.901	0.450	0.333	0.066	0.891	0.773	0.480	0.000					
MCW0123	0.772	0.169	0.093	0.588	0.744	0.362	0.141	0.945	1.000	0.431	1.000	0.620	0.115	0.674	1.000	0.385	0.402	0.403	0.438	0.499	0.763	0.700	0.033	0.000				
MCW0330	0.550	1.000	0.491	0.160	0.270	0.405	0.701	0.914	0.628	0.905	0.798	0.094	0.482	0.036	1.000	0.973	0.255	0.341	0.299	0.863	0.931	0.706	0.575	0.581	0.000			
MCW0165	0.983	0.951	0.026	0.664	0.470	0.253	0.553	0.577	0.860	0.521	0.516	0.677	0.310	0.827	0.121	0.202	0.396	0.696	0.340	0.614	0.000	0.978	0.195	0.674	0.745	0.000		
MCW0069	0.074	0.690	0.736	0.060	0.203	0.914	0.946	0.777	0.254	0.706	0.201	0.742	0.768	0.867	1.000	0.904	0.834	0.975	0.967	0.581	0.432	0.682	1.000	0.771	0.642	0.397	0.000	
ADL0278	0.943	0.081	0.976	0.191	0.843	0.210	0.157	0.860	0.281	0.863	0.317	0.534	0.651	0.687	0.770	0.594	0.292	0.578	0.974	0.347	0.313	0.701	0.712	0.104	0.328	0.277	0.146	0.000

Table S7. Pairwise differentiation of linkage disequilibrium of Mae Hong Son chickens (*Gallus gallus*, Linnaeus, 1758) [43] from Chiang Mai Livestock Research and Breeding Center, Chiang Mai, Thailand (CLRBC), based on 28 microsatellite loci. Numbers indicate *p*-values with 110 permutations.

Locus	MCW0111	MCW0247	LEI0234	LEI0166	MCW0222	MCW0037	ADL0268	MCW0034	MCW0206	MCW0016	MCW0103	MCW0295	LEI0094	MCW0098	MCW0078	MCW0081	LEI0192	MCW0014	MCW0183	MCW0067	ADL0112	MCW0216	MCW0104	MCW0123	MCW0330	MCW0165	MCW0069	ADL0278
MCW0111	0.000																											
MCW0247	0.157	0.000																										
LEI0234	0.340	0.050	0.000																									
LEI0166	0.085	0.674	0.112	0.000																								
MCW0222	0.534	0.598	0.104	0.611	0.000																							
MCW0037	0.234	0.017	0.002	0.081	0.064	0.000																						
ADL0268	0.063	0.307	0.010	0.028	0.202	0.045	0.000																					
MCW0034	0.580	0.363	0.879	0.44	0.777	1.000	0.177	0.000																				
MCW0206	0.475	0.624	0.333	0.499	1.000	0.598	0.598	0.003	0.000																			
MCW0016	0.133	0.425	0.015	0.055	0.470	0.004	0.036	0.900	0.299	0.000																		
MCW0103	0.065	0.756	0.114	0.027	0.220	0.032	0.034	0.799	0.237	0.044	0.000																	
MCW0295	0.331	0.558	0.362	0.724	0.327	0.766	0.227	1.000	1.000	0.421	0.531	0.000																
LEI0094	0.476	0.765	0.294	0.288	1.000	0.540	0.107	0.508	0.165	0.217	0.163	0.725	0.000															
MCW0098	0.531	0.600	0.112	0.613	1.000	0.065	0.420	1.000	0.535	0.045	0.687	0.552	0.659	0.000														
MCW0078	0.531	0.601	0.110	0.611	1.000	0.065	0.417	1.000	0.532	0.042	0.683	0.558	0.666	0.021	0.000													
MCW0081	0.032	0.206	0.016	0.028	0.463	0.064	0.014	0.523	0.297	0.051	0.042	0.203	0.748	0.4630	0.466	0.000												
LEI0192	0.228	0.520	0.003	0.553	0.062	0.014	0.133	0.961	0.032	0.135	0.266	0.658	0.541	0.064	0.063	0.118	0.000											
MCW0014	0.130	0.008	0.009	0.026	0.288	0.001	0.155	0.664	0.731	0.028	0.022	1.000	0.615	0.292	0.288	0.030	0.049	0.000										
MCW0183	0.096	0.015	0.001	0.057	0.460	0.011	0.042	0.344	0.231	0.064	0.068	0.768	0.396	0.455	0.462	0.004	0.020	0.001	0.000									
MCW0067	0.800	0.004	0.016	0.666	0.469	0.061	0.255	0.333	0.128	0.422	0.668	0.433	0.738	0.471	0.468	0.055	0.128	0.028	0.003	0.000								
ADL0112	0.441	0.216	0.007	0.504	0.279	0.046	0.350	1.000	0.141	0.025	1.000	0.134	1.000	0.113	0.114	0.024	0.084	0.198	0.045	0.030	0.000							
MCW0216	N/A	N/A	N/A	N/A	N/A	N/A	N/A	N/A	N/A	N/A	N/A	N/A	N/A	N/A	N/A	N/A	N/A	N/A	N/A	N/A	N/A	0.000						
MCW0104	1.000	1.000	0.331	N/A	N/A	N/A	0.331	N/A	N/A	N/A	N/A	N/A	N/A	N/A	N/A	0.331	0.332	N/A	0.333	0.336	0.331	N/A	0.000					
MCW0123	0.333	0.158	0.001	0.168	0.113	0.004	0.027	0.784	0.332	0.018	0.203	0.272	0.219	0.111	0.109	0.019	0.014	0.010	0.004	0.019	0.020	N/A	0.330	0.000				
MCW0330	0.795	0.206	0.015	0.666	0.044	0.068	0.015	0.684	0.799	0.432	0.298	0.036	0.905	0.469	0.464	0.050	0.125	0.224	0.070	0.054	0.028	N/A	0.0332	0.014	0.000			
MCW0165	1.000	0.004	0.048	0.500	0.467	0.019	0.595	0.614	0.500	0.221	0.472	0.808	0.808	0.466	0.466	0.217	0.276	0.010	0.020	0.004	0.129	N/A	N/A	0.050	0.222	0.000		
MCW0069	0.533	0.067	0.104	1.000	1.000	1.000	0.137	0.566	1.000	1.000	1.000	0.130	1.000	1.000	1.000	0.048	1.000	0.649	0.064	0.045	0.027	N/A	0.328	0.485	0.046	0.128	0.000	
ADL0278	0.231	0.017	0.004	0.085	0.066	0.0002	0.042	1.000	0.599	0.005	0.029	0.778	0.549	0.068	0.065	0.063	0.012	0.002	0.011	0.066	0.045	N/A	N/A	0.003	0.068	0.019	1.000	0.000

Table S8. Genetic diversity of Mae Hong Son chickens (n = 50) (*Gallus gallus*, Linnaeus, 1758) [43], based on 28 microsatellite loci. Detailed information on each individual chicken is presented in Table S1.

Populations	Locus	N	N_a	*AR*	N_{ea}	*I*	H_o	H_e	*M ratio*	*PIC*	*F*
**MHSF[1]**	MCW0111	10	5.000	4.900	3.448	1.400	0.800	0.710	0.357	0.670	-0.127
	MCW0247	10	4.000	3.895	2.667	1.106	1.000	0.625	0.667	0.551	-0.600
	LEI0234	10	6.000	5.695	3.030	1.373	0.900	0.670	0.042	0.628	-0.343
	LEI0166	10	3.000	2.800	1.227	0.394	0.200	0.185	0.300	0.177	-0.081
	MCW0222	10	3.000	2.900	2.151	0.845	0.600	0.535	0.500	0.436	-0.121
	MCW0037	10	3.000	2.995	2.020	0.857	0.300	0.505	0.750	0.442	0.406
	ADL0268	10	4.000	4.000	3.774	1.354	0.800	0.735	0.667	0.685	-0.088
	MCW0034	10	3.000	3.000	2.597	1.010	0.400	0.615	0.150	0.534	0.350
	MCW0206	10	5.000	4.895	3.509	1.392	1.000	0.715	0.313	0.668	-0.399
	MCW0016	10	4.000	3.989	2.899	1.187	1.000	0.655	0.286	0.592	-0.527
	MCW0103	10	3.000	3.000	2.632	1.030	1.000	0.620	0.750	0.548	-0.613
	MCW0295	10	3.000	3.000	1.869	0.819	0.400	0.465	0.500	0.420	0.140
	LEI0094	10	3.000	2.895	1.361	0.518	0.300	0.265	0.214	0.247	-0.132
	MCW0098	10	2.000	2.000	1.980	0.688	0.500	0.495	1.000	0.372	-0.010
	MCW0078	9	2.000	2.000	1.246	0.349	0.222	0.198	1.000	0.178	-0.125
	MCW0081	10	3.000	3.000	2.469	0.997	0.800	0.595	0.136	0.528	-0.345
	LEI0192	10	6.000	5.995	5.556	1.752	1.000	0.820	0.067	0.795	-0.220
	MCW0014	10	4.000	3.900	2.857	1.161	1.000	0.650	0.071	0.584	-0.538
	MCW0183	10	2.000	1.900	1.105	0.199	0.100	0.095	0.200	0.090	-0.053
	MCW0067	10	3.000	3.000	2.273	0.950	0.700	0.560	0.500	0.499	-0.250
	ADL0112	10	4.000	3.800	2.439	1.018	1.000	0.590	0.667	0.504	-0.695
	MCW0216	10	4.000	3.989	2.778	1.168	0.300	0.640	0.286	0.581	0.531
	MCW0104	9	6.000	6.000	2.793	1.351	0.333	0.642	0.273	0.610	0.481
	MCW0123	10	4.000	3.995	2.899	1.208	1.000	0.655	0.333	0.603	-0.527
	MCW0330	10	2.000	2.000	1.471	0.500	0.400	0.320	0.250	0.269	-0.250
	MCW0165	10	4.000	3.800	2.439	1.018	1.000	0.590	0.667	0.504	-0.695
	MCW0069	10	3.000	2.995	2.410	0.949	0.600	0.585	0.125	0.495	-0.026
	ADL0278	10	5.000	4.989	3.846	1.471	0.900	0.740	0.119	0.701	-0.216
	Mean	10	3.679	3.619	2.562	1.002	0.663	0.553	0.400	0.497	-0.181

Populations	Locus	N	N_a	*AR*	N_{ea}	*I*	H_o	H_e	*M ratio*	*PIC*	*F*
	SE	0	0.225	1.167	0.179	0.070	0.059	0.034	0.277	0.173	0.064
**MLRBC**[2]	MCW0111	30	6.000	5.800	2.528	1.193	0.667	0.604	0.300	0.555	-0.103
	MCW0247	30	4.000	4.000	3.103	1.233	1.000	0.678	0.667	0.618	-0.475
	LEI0234	29	10.000	9.855	5.644	1.938	0.931	0.823	0.064	0.801	-0.132
	LEI0166	30	3.000	3.000	2.182	0.888	0.433	0.542	0.214	0.460	0.200
	MCW0222	30	3.000	2.992	1.312	0.468	0.233	0.238	0.500	0.221	0.019
	MCW0037	30	4.000	3.992	2.203	0.988	0.300	0.546	0.667	0.489	0.451
	ADL0268	30	4.000	3.992	2.871	1.150	0.533	0.652	0.667	0.585	0.182
	MCW0034	27	7.000	7.000	4.326	1.610	0.185	0.769	0.318	0.732	0.759
	MCW0206	29	5.000	5.000	3.198	1.335	1.000	0.687	0.227	0.636	-0.455
	MCW0016	30	5.000	4.900	3.051	1.286	1.000	0.672	0.357	0.624	-0.488
	MCW0103	30	5.000	4.899	2.990	1.242	0.967	0.666	0.500	0.609	-0.452
	MCW0295	30	4.000	3.900	3.087	1.163	0.667	0.676	0.400	0.607	0.014
	LEI0094	30	6.000	5.982	2.439	1.187	0.400	0.590	0.176	0.542	0.322
	MCW0098	30	2.000	2.000	1.260	0.360	0.233	0.206	1.000	0.185	-0.132
	MCW0078	30	2.000	1.992	1.069	0.146	0.067	0.064	1.000	0.062	-0.034
	MCW0081	30	5.000	4.899	2.281	1.058	0.667	0.562	0.227	0.504	-0.187
	LEI0192	28	10.000	9.892	4.296	1.782	1.000	0.767	0.064	0.740	-0.303
	MCW0014	30	8.000	7.800	5.172	1.780	1.000	0.807	0.143	0.779	-0.240
	MCW0183	30	4.000	4.000	1.915	0.929	0.500	0.478	0.333	0.446	-0.047
	MCW0067	30	4.000	4.000	3.209	1.243	0.867	0.688	0.667	0.629	-0.259
	ADL0112	30	7.000	6.891	3.358	1.496	1.000	0.702	0.438	0.670	-0.424
	MCW0216	30	5.000	4.900	1.933	0.986	0.067	0.483	0.250	0.456	0.862
	MCW0104	27	14.000	14.000	6.312	2.176	0.630	0.842	0.241	0.825	0.252
	MCW0123	27	6.000	6.000	2.311	1.065	0.852	0.567	0.429	0.498	-0.502
	MCW0330	27	2.000	2.000	1.839	0.649	0.407	0.456	0.250	0.352	0.107
	MCW0165	30	6.000	5.900	3.309	1.434	1.000	0.698	0.375	0.662	-0.433
	MCW0069	27	6.000	6.000	2.982	1.284	0.741	0.665	0.300	0.612	-0.115
	ADL0278	27	6.000	6.000	2.549	1.230	0.667	0.608	0.143	0.565	-0.097
	Mean	29	5.464	5.414	2.955	1.189	0.643	0.598	0.390	0.552	-0.061
	SE	0	0.500	2.624	0.238	0.085	0.060	0.035	0.246	0.180	0.068
**CLRBC**[3]	MCW0111	10	2.000	1.976	1.724	0.611	0.000	0.420	3.500	0.332	1.000

Populations	Locus	N	N_a	*AR*	N_{ea}	*I*	H_o	H_e	*M ratio*	*PIC*	*F*
	MCW0247	10	3.000	2.376	1.942	0.791	0.700	0.485	0.500	0.406	-0.443
	LEI0234	10	6.000	4.563	4.545	1.643	0.600	0.780	0.140	0.748	0.231
	LEI0166	9	2.000	1.996	1.906	0.668	0.778	0.475	0.500	0.362	-0.636
	MCW0222	10	2.000	1.653	1.220	0.325	0.200	0.180	0.500	0.164	-0.111
	MCW0037	10	3.000	2.874	2.632	1.030	0.400	0.620	2.500	0.548	0.355
	ADL0268	10	3.000	2.935	2.899	1.081	0.500	0.655	1.250	0.580	0.237
	MCW0034	10	4.000	3.105	2.353	1.063	0.400	0.575	0.667	0.526	0.304
	MCW0206	10	2.000	1.990	1.835	0.647	0.700	0.455	0.250	0.351	-0.538
	MCW0016	10	3.000	2.890	2.778	1.055	1.000	0.640	2.000	0.563	-0.563
	MCW0103	10	2.000	1.949	1.600	0.562	0.100	0.375	2.000	0.305	0.733
	MCW0295	10	4.000	3.328	3.226	1.231	1.000	0.690	0.667	0.628	-0.449
	LEI0094	9	5.000	3.826	3.306	1.365	0.556	0.698	0.094	0.651	0.204
	MCW0098	10	3.000	2.648	2.381	0.943	1.000	0.580	0.750	0.492	-0.724
	MCW0078	10	3.000	2.648	2.381	0.943	1.000	0.580	0.750	0.492	-0.724
	MCW0081	10	2.000	1.976	1.724	0.611	0.200	0.420	0.091	0.332	0.524
	LEI0192	10	4.000	3.273	2.985	1.192	0.800	0.665	0.100	0.603	-0.203
	MCW0014	10	3.000	2.890	2.778	1.055	0.000	0.640	0.286	0.563	1.000
	MCW0183	10	4.000	3.203	2.381	1.089	0.800	0.580	0.333	0.535	-0.379
	MCW0067	10	3.000	2.890	2.778	1.055	1.000	0.640	0.600	0.563	-0.563
	ADL0112	9	3.000	2.559	1.780	0.778	0.556	0.438	0.500	0.396	-0.268
	MCW0216	10	1.000	1.000	1.000	0.000	0.400	0.000	#DIV/0!	0.000	#N/A
	MCW0104	4	3.000	3.000	2.667	1.040	1.000	0.625	0.136	0.555	-0.600
	MCW0123	10	5.000	4.283	4.444	1.544	1.000	0.775	0.313	0.739	-0.290
	MCW0330	10	2.000	1.999	2.000	0.693	0.600	0.500	0.250	0.375	-0.200
	MCW0165	10	2.000	1.976	1.724	0.611	0.600	0.420	1.000	0.332	-0.429
	MCW0069	10	3.000	2.396	2.151	0.845	0.900	0.535	0.088	0.436	-0.682
	ADL0278	10	4.000	3.203	2.381	0.611	0.600	0.580	0.333	0.535	-0.034
	Mean	10	3.071	2.693	2.411	0.913	0.621	0.537	0.744	0.468	-0.120
	SE	0	0.212	0.788	0.155	0.066	0.060	0.032	0.834	0.165	0.098

[1]MHSF = Mae Hong Son chickens derived from farmer community, Mae Hong Son.
[2]MLRBC = Mae Hong Son Livestock Research and Breeding Center, Mae Hong Son.
[3]CLRBC = Chiang Mai Livestock Research and Breeding Center, Chiang Mai.

Table S9. Comparison of genetic diversity parameters amongst Mae Hong Son chickens (*Gallus gallus*, Linnaeus, 1758) [43] populations, based on 28 microsatellite loci.

Observed	Population 1	Population 2	df	SE	t-test	*p*-value
	MHSF[1]	MLRBC[2]	0.020	0.022	0.924	0.369
heterozygosity (H_o)	MHSF[1]	CLRBC[3]	0.042	0.027	1.578	0.132
	MLRBC[2]	CLRBC[3]	0.022	0.022	1.004	0.331
	MHSF[1]	MLRBC[2]	-0.045	0.012	-3.598	$p<0.01$
heterozygosity (H_e)	MHSF[1]	CLRBC[3]	0.016	0.015	1.084	0.292
	MLRBC[2]	CLRBC[3]	0.061	0.012	5.096	$p<0.01$

[1]MHSF = Mae Hong Son chickens derived from farmer community, Mae Hong Son.
[2]MLRBC = Mae Hong Son Livestock Research and Breeding Center, Mae Hong Son.
[3]CLRBC = Chiang Mai Livestock Research and Breeding Center, Chiang Mai.

Table S10. Inbreeding coefficients (F_{IS}), relatedness (r), effective population size (N_e), and ratio of effective population size to census population (N_e/N) of Mae Hong Son chickens (*Gallus gallus*, Linnaeus, 1758) [43] derived from the Mae Hong Son farmer community, Mae Hong Son, Thailand (MHSF), Mae Hong Son Livestock Research and Breeding Center, Mae Hong Son, Thailand (MLRBC), and Chiang Mai Livestock Research and Breeding Center, Chiang Mai, Thailand (CLRBC). Estimates were calculated using NeEstimator version 2.1 [44], COANCESTRY version 1.0.1.9 [34], and GenAlEx version 6.5 [32]. Detailed information on each Mae Hong Son chicken is presented in Table S1.

**Population**	**N[1]**	**F_{IS}[2]**	**Relatedness (r)**	**Estimated N_e[3]**	**95% CIs[4] for N_e**	**N_e/N**
MHSF[4]	10	-0.113±0.037	-0.048±0.066	15.6	9.6	1.56
MLRBC[5]	30	-0.107±0.031	-0.018±0.040	186.8	90.0–110.6	6.22
CLRBC[6]	10	-0.045± 0.039	-0.052±0.150	1.9	1.5	0.19

[1]Sample size (N); [2]Inbreeding coefficient (F_{IS}); [3]Effective population size (N_e); [4]Confidence interval (CI).

[4]MHSF = Mae Hong Son chickens derived from farmer community, Mae Hong Son.
[5]MLRBC = Mae Hong Son Livestock Research and Breeding Center, Mae Hong Son.
[6]CLRBC = Chiang Mai Livestock Research and Breeding Center, Chiang Mai.

Table S11. Pairwise genetic relatedness (*r*) amongst Mae Hong Son chickens (*Gallus gallus*, Linnaeus, 1758) [43] populations derived from Mae Hong Son farmer community, Mae Hong Son, Thailand (MHSF). Detailed information on each individual chicken is presented in Table S1.

Sample 1	Sample 2	*r*
MHS M1F	MHS M2F	0.225
MHS M1F	MHS FM3F	-0.122
MHS M2F	MHS FM3F	-0.146
MHS M1F	MHS M4F	-0.047
MHS M2F	MHS M4F	-0.029
MHS FM3F	MHS M4F	-0.029
MHS M1F	MHS FM5F	0.007
MHS M2F	MHS FM5F	0.018
MHS FM3F	MHS FM5F	-0.078
MHS M4F	MHS FM5F	-0.012
MHS M1F	MHS M6F	-0.028
MHS M2F	MHS M6F	-0.025
MHS FM3F	MHS M6F	-0.015
MHS M4F	MHS M6F	-0.007
MHS FM5F	MHS M6F	-0.086
MHS M1F	MHS M7F	-0.063
MHS M2F	MHS M7F	-0.029
MHS FM3F	MHS M7F	-0.083
MHS M4F	MHS M7F	0.016
MHS FM5F	MHS M7F	-0.011
MHS M6F	MHS M7F	0.018
MHS M1F	MHS FM8F	-0.108
MHS M2F	MHS FM8F	-0.137
MHS FM3F	MHS FM8F	-0.065
MHS M4F	MHS FM8F	-0.106
MHS FM5F	MHS FM8F	-0.131
MHS M6F	MHS FM8F	-0.042
MHS M7F	MHS FM8F	-0.109
MHS M1F	MHS FM9F	-0.072
MHS M2F	MHS FM9F	-0.102
MHS FM3F	MHS FM9F	0.015
MHS M4F	MHS FM9F	-0.019
MHS FM5F	MHS FM9F	-0.067
MHS M6F	MHS FM9F	0.047
MHS M7F	MHS FM9F	0.016
MHS FM8F	MHS FM9F	-0.047
MHS M1F	MHS FM10F	-0.127
MHS M2F	MHS FM10F	-0.111
MHS FM3F	MHS FM10F	-0.100
MHS M4F	MHS FM10F	-0.071
MHS FM5F	MHS FM10F	-0.009
MHS M6F	MHS FM10F	-0.107

**Sample 1**	**Sample 2**	***r***
MHS M7F	MHS FM10F	-0.091
MHS FM8F	MHS FM10F	0.002
MHS FM9F	MHS FM10F	-0.110

Table S12. Pairwise genetic relatedness (*r*) amongst Mae Hong Son chickens (*Gallus gallus*, Linnaeus, 1758) [43] populations from Mae Hong Son Livestock Research and Breeding Center, Mae Hong Son, Thailand (MLRBC). Detailed information on each individual chicken is presented in Table S1.

Sample 1	Sample 2	*r*
MHS M1	MHS M2	-0.002
MHS M1	MHS M3	-0.056
MHS M2	MHS M3	0.020
MHS M1	MHS M4	-0.002
MHS M2	MHS M4	0.108
MHS M3	MHS M4	0.031
MHS M1	MHS M5	0.051
MHS M2	MHS M5	-0.037
MHS M3	MHS M5	-0.102
MHS M4	MHS M5	-0.049
MHS M1	MHS M6	-0.075
MHS M2	MHS M6	-0.064
MHS M3	MHS M6	0.023
MHS M4	MHS M6	0.039
MHS M5	MHS M6	-0.032
MHS M1	MHS M7	-0.031
MHS M2	MHS M7	-0.030
MHS M3	MHS M7	0.005
MHS M4	MHS M7	-0.029
MHS M5	MHS M7	-0.078
MHS M6	MHS M7	-0.065
MHS M1	MHS M8	-0.008
MHS M2	MHS M8	0.051
MHS M3	MHS M8	0.022
MHS M4	MHS M8	-0.049
MHS M5	MHS M8	-0.015
MHS M6	MHS M8	-0.039
MHS M7	MHS M8	-0.019
MHS M1	MHS M9	-0.038
MHS M2	MHS M9	0.013
MHS M3	MHS M9	-0.080
MHS M4	MHS M9	-0.004
MHS M5	MHS M9	0.062
MHS M6	MHS M9	-0.063
MHS M7	MHS M9	-0.061
MHS M8	MHS M9	-0.006
MHS M1	MHS M10	-0.084
MHS M2	MHS M10	-0.004
MHS M3	MHS M10	-0.055
MHS M4	MHS M10	-0.067
MHS M5	MHS M10	-0.054
MHS M6	MHS M10	-0.025

Sample 1	Sample 2	*r*
MHS M7	MHS M10	-0.059
MHS M8	MHS M10	0.056
MHS M9	MHS M10	-0.027
MHS M1	MHS M11	0.027
MHS M2	MHS M11	0.002
MHS M3	MHS M11	0.014
MHS M4	MHS M11	0.047
MHS M5	MHS M11	-0.042
MHS M6	MHS M11	-0.033
MHS M7	MHS M11	-0.008
MHS M8	MHS M11	-0.032
MHS M9	MHS M11	0.009
MHS M10	MHS M11	-0.040
MHS M1	MHS M12	-0.063
MHS M2	MHS M12	0.017
MHS M3	MHS M12	-0.006
MHS M4	MHS M12	-0.039
MHS M5	MHS M12	-0.072
MHS M6	MHS M12	-0.047
MHS M7	MHS M12	-0.046
MHS M8	MHS M12	-0.016
MHS M9	MHS M12	0.061
MHS M10	MHS M12	-0.051
MHS M11	MHS M12	0.049
MHS M1	MHS M13	-0.037
MHS M2	MHS M13	-0.062
MHS M3	MHS M13	-0.053
MHS M4	MHS M13	-0.046
MHS M5	MHS M13	-0.026
MHS M6	MHS M13	-0.033
MHS M7	MHS M13	0.022
MHS M8	MHS M13	-0.045
MHS M9	MHS M13	-0.004
MHS M10	MHS M13	0.025
MHS M11	MHS M13	-0.070
MHS M12	MHS M13	-0.028
MHS M1	MHS M14	-0.046
MHS M2	MHS M14	-0.015
MHS M3	MHS M14	-0.036
MHS M4	MHS M14	-0.045
MHS M5	MHS M14	-0.045
MHS M6	MHS M14	-0.052
MHS M7	MHS M14	-0.042
MHS M8	MHS M14	-0.034
MHS M9	MHS M14	-0.016
MHS M10	MHS M14	-0.057
MHS M11	MHS M14	0.001

Sample 1	Sample 2	*r*
MHS M12	MHS M14	0.019
MHS M13	MHS M14	-0.002
MHS M1	MHS M15	0.025
MHS M2	MHS M15	-0.030
MHS M3	MHS M15	-0.060
MHS M4	MHS M15	-0.013
MHS M5	MHS M15	0.015
MHS M6	MHS M15	0.016
MHS M7	MHS M15	-0.034
MHS M8	MHS M15	-0.113
MHS M9	MHS M15	-0.055
MHS M10	MHS M15	-0.045
MHS M11	MHS M15	-0.018
MHS M12	MHS M15	-0.076
MHS M13	MHS M15	-0.006
MHS M14	MHS M15	-0.027
MHS M1	MHS FM1	-0.044
MHS M2	MHS FM1	-0.067
MHS M3	MHS FM1	0.027
MHS M4	MHS FM1	-0.060
MHS M5	MHS FM1	-0.013
MHS M6	MHS FM1	-0.009
MHS M7	MHS FM1	0.065
MHS M8	MHS FM1	-0.079
MHS M9	MHS FM1	-0.039
MHS M10	MHS FM1	0.016
MHS M11	MHS FM1	-0.062
MHS M12	MHS FM1	-0.075
MHS M13	MHS FM1	0.058
MHS M14	MHS FM1	0.022
MHS M15	MHS FM1	0.060
MHS M1	MHS FM2	-0.022
MHS M2	MHS FM2	0.016
MHS M3	MHS FM2	-0.023
MHS M4	MHS FM2	0.008
MHS M5	MHS FM2	-0.011
MHS M6	MHS FM2	-0.002
MHS M7	MHS FM2	0.004
MHS M8	MHS FM2	-0.061
MHS M9	MHS FM2	0.013
MHS M10	MHS FM2	-0.058
MHS M11	MHS FM2	-0.047
MHS M12	MHS FM2	-0.013
MHS M13	MHS FM2	-0.039
MHS M14	MHS FM2	-0.002
MHS M15	MHS FM2	-0.024

Sample 1	Sample 2	*r*
MHS FM1	MHS FM2	-0.084
MHS M1	MHS FM3	-0.040
MHS M2	MHS FM3	0.012
MHS M3	MHS FM3	-0.011
MHS M4	MHS FM3	-0.008
MHS M5	MHS FM3	-0.046
MHS M6	MHS FM3	-0.103
MHS M7	MHS FM3	-0.028
MHS M8	MHS FM3	-0.039
MHS M9	MHS FM3	-0.036
MHS M10	MHS FM3	-0.066
MHS M11	MHS FM3	0.013
MHS M12	MHS FM3	0.004
MHS M13	MHS FM3	-0.016
MHS M14	MHS FM3	0.027
MHS M15	MHS FM3	-0.083
MHS FM1	MHS FM3	-0.002
MHS FM2	MHS FM3	-0.001
MHS M1	MHS FM4	0.052
MHS M2	MHS FM4	-0.021
MHS M3	MHS FM4	-0.010
MHS M4	MHS FM4	0.059
MHS M5	MHS FM4	0.015
MHS M6	MHS FM4	-0.046
MHS M7	MHS FM4	0.036
MHS M8	MHS FM4	0.034
MHS M9	MHS FM4	0.031
MHS M10	MHS FM4	-0.007
MHS M11	MHS FM4	0.093
MHS M12	MHS FM4	-0.070
MHS M13	MHS FM4	-0.052
MHS M14	MHS FM4	-0.043
MHS M15	MHS FM4	-0.058
MHS FM1	MHS FM4	-0.100
MHS FM2	MHS FM4	-0.011
MHS FM3	MHS FM4	-0.064
MHS M1	MHS FM5	-0.012
MHS M2	MHS FM5	-0.008
MHS M3	MHS FM5	-0.040
MHS M4	MHS FM5	-0.084
MHS M5	MHS FM5	0.007
MHS M6	MHS FM5	0.073
MHS M7	MHS FM5	-0.097
MHS M8	MHS FM5	-0.029
MHS M9	MHS FM5	-0.018
MHS M10	MHS FM5	-0.017
MHS M11	MHS FM5	-0.059

**Sample 1**	**Sample 2**	***r***
MHS M12	MHS FM5	-0.053
MHS M13	MHS FM5	-0.042
MHS M14	MHS FM5	0.014
MHS M15	MHS FM5	-0.052
MHS FM1	MHS FM5	-0.057
MHS FM2	MHS FM5	-0.001
MHS FM3	MHS FM5	-0.032
MHS FM4	MHS FM5	-0.006
MHS M1	MHS FM6	-0.067
MHS M2	MHS FM6	-0.118
MHS M3	MHS FM6	-0.013
MHS M4	MHS FM6	-0.087
MHS M5	MHS FM6	-0.003
MHS M6	MHS FM6	-0.045
MHS M7	MHS FM6	-0.040
MHS M8	MHS FM6	-0.005
MHS M9	MHS FM6	-0.031
MHS M10	MHS FM6	0.011
MHS M11	MHS FM6	-0.020
MHS M12	MHS FM6	-0.033
MHS M13	MHS FM6	-0.030
MHS M14	MHS FM6	0.007
MHS M15	MHS FM6	0.003
MHS FM1	MHS FM6	-0.029
MHS FM2	MHS FM6	0.041
MHS FM3	MHS FM6	-0.052
MHS FM4	MHS FM6	-0.055
MHS FM5	MHS FM6	-0.040
MHS M1	MHS FM7	-0.066
MHS M2	MHS FM7	-0.043
MHS M3	MHS FM7	0.046
MHS M4	MHS FM7	0.056
MHS M5	MHS FM7	-0.004
MHS M6	MHS FM7	0.013
MHS M7	MHS FM7	-0.071
MHS M8	MHS FM7	-0.054
MHS M9	MHS FM7	0.009
MHS M10	MHS FM7	-0.018
MHS M11	MHS FM7	-0.079
MHS M12	MHS FM7	-0.040
MHS M13	MHS FM7	-0.065
MHS M14	MHS FM7	-0.047
MHS M15	MHS FM7	0.016
MHS FM1	MHS FM7	-0.058
MHS FM2	MHS FM7	0.100
MHS FM3	MHS FM7	-0.025

Sample 1	Sample 2	*r*
MHS FM4	MHS FM7	0.066
MHS FM5	MHS FM7	-0.022
MHS FM6	MHS FM7	0.033
MHS M1	MHS FM8	-0.015
MHS M2	MHS FM8	0.012
MHS M3	MHS FM8	-0.032
MHS M4	MHS FM8	-0.011
MHS M5	MHS FM8	0.045
MHS M6	MHS FM8	-0.062
MHS M7	MHS FM8	-0.023
MHS M8	MHS FM8	-0.006
MHS M9	MHS FM8	-0.021
MHS M10	MHS FM8	-0.007
MHS M11	MHS FM8	-0.087
MHS M12	MHS FM8	-0.036
MHS M13	MHS FM8	-0.022
MHS M14	MHS FM8	-0.003
MHS M15	MHS FM8	0.028
MHS FM1	MHS FM8	-0.028
MHS FM2	MHS FM8	-0.043
MHS FM3	MHS FM8	0.073
MHS FM4	MHS FM8	-0.018
MHS FM5	MHS FM8	0.015
MHS FM6	MHS FM8	0.027
MHS FM7	MHS FM8	-0.038
MHS M1	MHS FM9	-0.004
MHS M2	MHS FM9	-0.026
MHS M3	MHS FM9	-0.017
MHS M4	MHS FM9	-0.072
MHS M5	MHS FM9	0.022
MHS M6	MHS FM9	-0.070
MHS M7	MHS FM9	0.067
MHS M8	MHS FM9	0.002
MHS M9	MHS FM9	0.020
MHS M10	MHS FM9	0.011
MHS M11	MHS FM9	-0.059
MHS M12	MHS FM9	-0.044
MHS M13	MHS FM9	0.025
MHS M14	MHS FM9	0.020
MHS M15	MHS FM9	-0.068
MHS FM1	MHS FM9	-0.014
MHS FM2	MHS FM9	-0.034
MHS FM3	MHS FM9	0.000
MHS FM4	MHS FM9	-0.019
MHS FM5	MHS FM9	0.010
MHS FM6	MHS FM9	0.039
MHS FM7	MHS FM9	-0.050

Sample 1	Sample 2	*r*
MHS FM8	MHS FM9	0.015
MHS M1	MHS FM10	-0.066
MHS M2	MHS FM10	-0.004
MHS M3	MHS FM10	-0.063
MHS M4	MHS FM10	-0.036
MHS M5	MHS FM10	-0.014
MHS M6	MHS FM10	0.007
MHS M7	MHS FM10	-0.018
MHS M8	MHS FM10	0.021
MHS M9	MHS FM10	0.012
MHS M10	MHS FM10	-0.009
MHS M11	MHS FM10	-0.023
MHS M12	MHS FM10	0.000
MHS M13	MHS FM10	-0.038
MHS M14	MHS FM10	-0.059
MHS M15	MHS FM10	-0.039
MHS FM1	MHS FM10	-0.050
MHS FM2	MHS FM10	-0.044
MHS FM3	MHS FM10	-0.036
MHS FM4	MHS FM10	-0.022
MHS FM5	MHS FM10	0.013
MHS FM6	MHS FM10	-0.025
MHS FM7	MHS FM10	-0.026
MHS FM8	MHS FM10	-0.035
MHS FM9	MHS FM10	-0.006
MHS M1	MHS FM11	-0.062
MHS M2	MHS FM11	-0.004
MHS M3	MHS FM11	-0.065
MHS M4	MHS FM11	0.033
MHS M5	MHS FM11	0.008
MHS M6	MHS FM11	-0.018
MHS M7	MHS FM11	-0.035
MHS M8	MHS FM11	-0.029
MHS M9	MHS FM11	0.017
MHS M10	MHS FM11	-0.014
MHS M11	MHS FM11	-0.015
MHS M12	MHS FM11	0.015
MHS M13	MHS FM11	0.036
MHS M14	MHS FM11	-0.043
MHS M15	MHS FM11	-0.034
MHS FM1	MHS FM11	-0.026
MHS FM2	MHS FM11	-0.022
MHS FM3	MHS FM11	-0.051
MHS FM4	MHS FM11	0.016
MHS FM5	MHS FM11	-0.046
MHS FM6	MHS FM11	-0.008

Sample 1	Sample 2	*r*
MHS FM7	MHS FM11	0.033
MHS FM8	MHS FM11	-0.018
MHS FM9	MHS FM11	-0.026
MHS FM10	MHS FM11	-0.024
MHS M1	MHS FM12	0.010
MHS M2	MHS FM12	-0.029
MHS M3	MHS FM12	-0.071
MHS M4	MHS FM12	-0.028
MHS M5	MHS FM12	0.007
MHS M6	MHS FM12	-0.054
MHS M7	MHS FM12	0.057
MHS M8	MHS FM12	-0.030
MHS M9	MHS FM12	-0.027
MHS M10	MHS FM12	-0.069
MHS M11	MHS FM12	-0.002
MHS M12	MHS FM12	0.005
MHS M13	MHS FM12	-0.026
MHS M14	MHS FM12	-0.032
MHS M15	MHS FM12	-0.005
MHS FM1	MHS FM12	-0.001
MHS FM2	MHS FM12	-0.006
MHS FM3	MHS FM12	0.000
MHS FM4	MHS FM12	-0.022
MHS FM5	MHS FM12	-0.084
MHS FM6	MHS FM12	-0.009
MHS FM7	MHS FM12	-0.079
MHS FM8	MHS FM12	0.018
MHS FM9	MHS FM12	-0.048
MHS FM10	MHS FM12	0.076
MHS FM11	MHS FM12	0.039
MHS M1	MHS FM13	-0.009
MHS M2	MHS FM13	-0.040
MHS M3	MHS FM13	-0.015
MHS M4	MHS FM13	-0.051
MHS M5	MHS FM13	-0.072
MHS M6	MHS FM13	-0.017
MHS M7	MHS FM13	-0.027
MHS M8	MHS FM13	-0.063
MHS M9	MHS FM13	-0.042
MHS M10	MHS FM13	-0.067
MHS M11	MHS FM13	-0.015
MHS M12	MHS FM13	0.045
MHS M13	MHS FM13	0.196
MHS M14	MHS FM13	0.034
MHS M15	MHS FM13	-0.004
MHS FM1	MHS FM13	-0.011
MHS FM2	MHS FM13	0.026

Sample 1	Sample 2	*r*
MHS FM3	MHS FM13	0.036
MHS FM4	MHS FM13	-0.060
MHS FM5	MHS FM13	-0.008
MHS FM6	MHS FM13	-0.035
MHS FM7	MHS FM13	-0.075
MHS FM8	MHS FM13	-0.066
MHS FM9	MHS FM13	0.035
MHS FM10	MHS FM13	-0.027
MHS FM11	MHS FM13	0.016
MHS FM12	MHS FM13	-0.028
MHS M1	MHS FM14	0.030
MHS M2	MHS FM14	0.028
MHS M3	MHS FM14	-0.043
MHS M4	MHS FM14	-0.056
MHS M5	MHS FM14	-0.056
MHS M6	MHS FM14	-0.086
MHS M7	MHS FM14	-0.050
MHS M8	MHS FM14	0.044
MHS M9	MHS FM14	-0.048
MHS M10	MHS FM14	-0.012
MHS M11	MHS FM14	-0.039
MHS M12	MHS FM14	-0.031
MHS M13	MHS FM14	-0.047
MHS M14	MHS FM14	0.003
MHS M15	MHS FM14	0.009
MHS FM1	MHS FM14	-0.057
MHS FM2	MHS FM14	0.026
MHS FM3	MHS FM14	0.058
MHS FM4	MHS FM14	-0.072
MHS FM5	MHS FM14	0.026
MHS FM6	MHS FM14	0.080
MHS FM7	MHS FM14	-0.061
MHS FM8	MHS FM14	0.062
MHS FM9	MHS FM14	-0.020
MHS FM10	MHS FM14	-0.067
MHS FM11	MHS FM14	-0.007
MHS FM12	MHS FM14	-0.032
MHS FM13	MHS FM14	0.009
MHS M1	MHS FM15	0.021
MHS M2	MHS FM15	-0.004
MHS M3	MHS FM15	-0.030
MHS M4	MHS FM15	-0.014
MHS M5	MHS FM15	0.054
MHS M6	MHS FM15	-0.070
MHS M7	MHS FM15	-0.058
MHS M8	MHS FM15	-0.026

**Sample 1**	**Sample 2**	***r***
MHS M9	MHS FM15	0.009
MHS M10	MHS FM15	-0.031
MHS M11	MHS FM15	-0.049
MHS M12	MHS FM15	-0.075
MHS M13	MHS FM15	-0.065
MHS M14	MHS FM15	0.006
MHS M15	MHS FM15	0.015
MHS FM1	MHS FM15	0.039
MHS FM2	MHS FM15	-0.050
MHS FM3	MHS FM15	-0.056
MHS FM4	MHS FM15	0.017
MHS FM5	MHS FM15	0.000
MHS FM6	MHS FM15	0.021
MHS FM7	MHS FM15	0.005
MHS FM8	MHS FM15	-0.004
MHS FM9	MHS FM15	0.013
MHS FM10	MHS FM15	-0.054
MHS FM11	MHS FM15	0.029
MHS FM12	MHS FM15	-0.093
MHS FM13	MHS FM15	-0.002
MHS FM14	MHS FM15	0.077

Table S13. Pairwise genetic relatedness (*r*) amongst Mae Hong Son chickens (*Gallus gallus*, Linnaeus, 1758) [43] populations from Chiang Mai Livestock Research and Breeding Center, Chiang Mai, Thailand (CLRBC). Detailed information on each individual chicken is presented in Table S1.

**Sample 1**	**Sample 2**	***r***
MHS1	MHS2	0.283
MHS1	MHS3	-0.116
MHS2	MHS3	-0.142
MHS1	MHS4	-0.116
MHS2	MHS4	-0.073
MHS3	MHS4	-0.081
MHS1	MHS5	-0.146
MHS2	MHS5	-0.104
MHS3	MHS5	-0.103
MHS4	MHS5	0.441
MHS1	MHS6	-0.140
MHS2	MHS6	-0.148
MHS3	MHS6	0.248
MHS4	MHS6	-0.133
MHS5	MHS6	-0.077
MHS1	MHS7	-0.106
MHS2	MHS7	-0.089
MHS3	MHS7	-0.036
MHS4	MHS7	-0.137
MHS5	MHS7	-0.089
MHS6	MHS7	0.047
MHS1	MHS8	-0.068
MHS2	MHS8	-0.080
MHS3	MHS8	0.012
MHS4	MHS8	-0.135
MHS5	MHS8	-0.150
MHS6	MHS8	0.059
MHS7	MHS8	0.242
MHS1	MHS9	-0.003
MHS2	MHS9	-0.003
MHS3	MHS9	-0.235
MHS4	MHS9	-0.132
MHS5	MHS9	-0.168
MHS6	MHS9	-0.151
MHS7	MHS9	-0.162
MHS8	MHS9	-0.119
MHS1	MHS10	0.033
MHS2	MHS10	0.013
MHS3	MHS10	-0.199
MHS4	MHS10	-0.096
MHS5	MHS10	-0.147
MHS6	MHS10	-0.140

**Sample 1**	**Sample 2**	***r***
MHS7	MHS10	-0.162
MHS8	MHS10	-0.142
MHS9	MHS10	0.389

Table S14. Relatedness (*r*) distributions of Mae Hong Son chickens (*Gallus gallus*, Linnaeus, 1758) [43].

Population 1	Population 2	Relatedness (*r*)	
		**Density**	***p*-value**
MHSF[1]	MLRBC[2]	0.300	0.509
MHSF[1]	CLRBC[3]	0.700	<0.05
MLRBC[2]	CLRBC[3]	0.700	<0.05

[1]MHSF = Mae Hong Son chickens derived from farmer community, Mae Hong Son.
[2]MLRBC = Mae Hong Son Livestock Research and Breeding Center, Mae Hong Son.
[3]CLRBC = Chiang Mai Livestock Research and Breeding Center, Chiang Mai.

Table S15. Inbreeding coefficients (F_{IS}) of Mae Hong Son chickens (n = 10) (*Gallus gallus*, Linnaeus, 1758) [43] derived from Mae Hong Son farmer community, Mae Hong Son, Thailand (MHSF). Detailed information on each individual chicken is presented in Table S1.

**Individual**	F_{IS}
MHSM1F	-0.0703
MHSM2F	-0.0804
MHSFM3F	-0.0539
MHSM4F	-0.1352
MHSFM5F	-0.1672
MHSM6F	-0.1498
MHSM7F	-0.1156
MHSFM8F	-0.1387
MHSFM9F	-0.0891
MHSFM10F	-0.1314

Table S16. Inbreeding coefficients (F_{IS}) of Mae Hong Son chickens (n = 30) (*Gallus gallus*, Linnaeus, 1758) [43] from Mae Hong Son Livestock Research and Breeding Center, Mae Hong Son, Thailand (MLRBC). Detailed information on each individual chicken is presented in Table S1.

**Individual**	F_{IS}
MHSM1	-0.0993
MHSM2	-0.0888
MHSM3	-0.1259
MHSM4	-0.143
MHSM5	-0.0969
MHSM6	-0.1175
MHSM7	-0.1518
MHSM8	-0.0692
MHSM9	-0.0897
MHSM10	-0.0803
MHSM11	-0.0874
MHSM12	-0.1367
MHSM13	-0.1451
MHSM14	-0.0664
MHSM15	-0.1032
MHSFM1	-0.1163
MHSFM2	-0.1221
MHSFM3	-0.1522
MHSFM4	-0.1197
MHSFM5	-0.004
MHSFM6	-0.0838
MHSFM7	-0.0972
MHSFM8	-0.1287
MHSFM9	-0.1275
MHSFM10	-0.0894
MHSFM11	-0.1163
MHSFM12	-0.0881
MHSFM13	-0.1457
MHSFM14	-0.1302
MHSFM15	-0.1122

Table S17. Inbreeding coefficients (F_{IS}) of Mae Hong Son chickens (n = 10) (*Gallus gallus*, Linnaeus, 1758) [43] from Chiang Mai Livestock Research and Breeding Center, Chiang Mai, Thailand (CLRBC). Detailed information on each individual chicken is presented in Table S1.

**Individual**	F_{IS}
MHS1	-0.0564
MHS2	-0.0248
MHS3	-0.008
MHS4	-0.0294
MHS5	-0.0667
MHS6	-0.0097
MHS7	-0.0489
MHS8	0.0068
MHS9	-0.1115
MHS10	-0.0968

Table S18. F_{IS} distributions for Mae Hong Son chickens (*Gallus gallus*, Linnaeus, 1758) [43].

Population 1	Population 2	Inbreeding coefficient (F_{IS})	
		**Density**	***p*-value**
MHSF[1]	MLRBC[2]	0.360	<0.01
MHSF[1]	CLRBC[3]	0.356	<0.01
MLRBC[2]	CLRBC[3]	0.631	<0.01

[1]MHSF = Mae Hong Son chickens derived from farmer community, Mae Hong Son.
[2]MLRBC = Mae Hong Son Livestock Research and Breeding Center, Mae Hong Son.
[3]CLRBC = Chiang Mai Livestock Research and Breeding Center, Chiang Mai.

Table S19. Pairwise genetic differentiation (F_{ST}), pairwise F_{ST}^{ENA} values with ENA correction for null alleles, and R_{ST} values using FSTAT version 2.9.3 [29] for Mae Hong Son chickens (*Gallus gallus*, Linnaeus, 1758) [43], subjected to captive breeding, based on 28 microsatellite loci. Numbers indicate *p*-values with 110 permutations. Detailed information on each individual chicken is presented in Table S1.

Population 1	Population 2	F_{ST}	F_{ST}^{ENA}	R_{ST}
MHSF[1]	MLRBC[2]	0.084*	0.084	0.074
MHSF[1]	CLRBC[3]	0.182*	0.195	0.377
MLRBC[2]	CLRBC[3]	0.145*	0.148	0.379

*p value < 0.05.

[1]MHSF = Mae Hong Son chickens derived from farmer community, Mae Hong Son.
[2]MLRBC = Mae Hong Son Livestock Research and Breeding Center, Mae Hong Son.
[3]CLRBC = Chiang Mai Livestock Research and Breeding Center, Chiang Mai .

Table S20. Analysis of molecular variance (AMOVA) for Mae Hong Son chickens (*Gallus gallus*, Linnaeus, 1758) [43], based on 28 microsatellite loci, using Arlequin version 3.5.2.2 [11]. Detailed information on each individual chicken is presented in Table S1.

Source of variation	df	Sum of squares	Variance components	Percentage of variation
Among populations	2	174.733	5.007	22%
Within populations	47	811.567	17.267	78%
Total	49	986.300	22.274	100%

Table S21. Results of pairwise population Nei's genetic distance (*D*) in Mae Hong Son chickens (n = 50) (*Gallus gallus*, Linnaeus, 1758) [43], based on 28 microsatellite loci, using GenAlEx version 6.5 [32].

Nei D	MHSF[1]	MLRBC[2]	CLRBC[3]
MHSF[1]	0.000		
MLRBC[2]	0.193	0.000	
CLRBC[3]	0.502	0.375	0.000

[1]MHSF = Mae Hong Son chickens derived from farmer community, Mae Hong Son.
[2]MLRBC = Mae Hong Son Livestock Research and Breeding Center, Mae Hong Son.
[3]CLRBC = Chiang Mai Livestock Research and Breeding Center, Chiang Mai.

Table S22. Observed and expected heterozygosity in Mae Hong Son chickens (*Gallus gallus*, Linnaeus, 1758) [43] based on 28 microsatellite loci and genetic bottlenecks of each individual chicken. Data were calculated using Bottleneck version 1.2.02 [38]. Detailed information on each Mae Hong Son chickens is presented in Table S1.

Species	Locality	H_o	H_e	*p-value*	Wilcoxon test		Mode-shift test
					TPM	SMM	
Gallus gallus	MHSF[1]	0.663±0.059	0.553±0.034	$p < 0.05$	0.089	0.212	shifted mode
	MLRBC[2]	0.643±0.060	0.598±0.035	$p < 0.05$	0.023	0.000	normal L-shaped distribution
	CLRBC[3]	0.621±0.060	0.537±0.032	$p < 0.05$	0.000	0.000	shifted mode

H_o = Observed heterozygosity, H_e = Expected heterozygosity, TPM = Two-phased mutation model, SMM = Stepwise mutation model.

[1]MHSF = Mae Hong Son chickens derived from farmer community, Mae Hong Son.
[2]MLRBC = Mae Hong Son Livestock Research and Breeding Center, Mae Hong Son.
[3]CLRBC = Chiang Mai Livestock Research and Breeding Center, Chiang Mai.

References

1 Phillips, S.J.; Dudík, M. Modeling of species distributions with Maxent: new extensions and a comprehensive evaluation. *Ecography* **2008**, *31*, 161–175. doi: 10.1111/j.0906-7590.2008.5203.x

2 Pearson, R.G.; Raxworthy, C.J.; Nakamura, M.; Townsend Peterson, A. Predicting species distributions from small numbers of occurrence records: a test case using cryptic geckos in Madagascar. *J. Biogeogr*. **2007**, *34*, 102–117. doi: 10.1111/j.1365-2699.2006.01594.x

3 Baldwin, R.A. Use of maximum entropy modeling in wildlife research. *Entropy* **2009**, *11*, 854–866. doi: 10.3390/e11040854

4 Araújo, M.B.; New, M. Ensemble forecasting of species distributions. *Trends Ecol. Evol.* **2007**, *22*, 42–47. doi: 10.1016/j.tree.2006.09.010

5 Marmion, M.; Parviainen, M.; Luoto, M.; Heikkinen, R.K.; Thuiller, W. Evaluation of consensus methods in predictive species distribution modelling. *Divers. Distrib.* **2009**, *15*, 59–69. doi: 10.1111/j.1472-4642.2008.00491.x

6 Fielding, A. H.; Bell, J.F. A review of methods for the assessment of prediction errors in conservation presence/absence models. *Environ. Conserv.* **1997**, *24*, 38–49. doi: 10.1017/S0376892997000088

7 Li, Y.; Li, M.; Li, C.; Liu, Z. Optimized maxent model predictions of climate change impacts on the suitable distribution of *Cunninghamia lanceolata* in China. *Forests* **2020**, *11*, 302.

8 Tamura, K.; Stecher, G.; Kumar, S. MEGA11: Molecular evolutionary genetics analysis version 11. *Mol. Biol. Evol.* **2021**, *38*, 3022–3027. doi:10.1093/molbev/msab120.

9 Rozas, J.; Ferrer-Mata, A.; Sánchez-DelBarrio, J.C.; Guirao-Rico, S.; Librado, P.; Ramos-Onsins, S.E.; Sánchez-Gracia, A. DnaSP 6: DNA sequence polymorphism analysis of large data sets. *Mol. Biol. Evol.* **2017**, *34*, 3299–3302. doi:10.1093/molbev/msx248.

10 Clement, M.; Snell, Q.; Walker, P.; Posada, D.; Crandall, K. TCS: Estimating gene genealogies. *Proceedings of the 16th International Parallel and Distributed Processing Symposium* **2002**, *2*, 184.

11 Excoffier, L.; Lischer, H.E. Arlequin suite ver 3.5: a new series of programs to perform population genetics analyses under Linux and Windows. *Mol. Ecol. Resour.* **2010**, *10*, 564–567. doi:10.1111/j.1755-0998.2010.02847.x

12 Weir, B.S.; Cockerhamm, C.C. Estimating *F*-statistics for the analysis of population structure. *Evolution* **1984**, *38*, 1358–1370. doi:10.2307/2408641.

13 Excoffier, L.; Smouse, P.E.; Quattro, J.M. Analysis of molecular variance inferred from metric distances among DNA haplotypes: Application to human mitochondrial DNA restriction data. *Genetics* **1992**, *131*, 479–491. doi:10.1093/genetics/131.2.479.

14 Tajima, F. Statistical method for testing the neutral mutation hypothesis by DNA polymorphism. *J. Genet.* **1989**, *123*, 585–595. doi:10.1093/genetics/123.3.585.

15 Fu, Y.X.; Li, W.H. Statistical tests of neutrality of mutations. *J. Genet.* **1993**, *133*, 693–709. doi:10.1093/genetics/133.3.693.

16 Fu, Y.X. Statistical tests of neutrality of mutations against population growth, hitchhiking and background selection. *J. Genet.* **1997**, *147*, 915–925. doi:10.1093/genetics/147.2.915.

17 Ramos-Onsins, S.; Rozas, J. Statistical properties of new neutrality tests against population growth. *Mol. Biol. Evol.* **2002**, *19*, 2092–2100. doi:10.1093/oxfordjournals.molbev.a004034.

18 Rogers, A.; Harpending, H. Population growth makes waves in the distribution of pairwise genetic differences. *Mol. Biol. Evol.* **1992** *9*, 552–569. doi:10.1093/oxfordjournals.molbev.a040727.

19 Wakeley, J.; Hey, J. Estimating ancestral population parameters. *J. Genet.* **1997**, *145*, 847–855. doi:10.1093/genetics/145.3.847.

20 Heled, J.; Drummond, A.J. Bayesian inference of population size history from multiple loci. *BMC Evol. Biol.* **2008**, *8*, 1–15. doi:10.1186/1471-2148-8-289.

21 Bouckaert, R.; Vaughan, T.G.; Barido-Sottani, J.; Duchne, S.; Fourment, M.; Gavryushkina, A.; Heled, J.; Jones, G.; Kühnert, D.; De Maio, N.; et al. BEAST 2.5: An advanced software platform for Bayesian evolutionary analysis. *PloS Comput. Biol.* **2019**, *15*, e1006650. doi:10.1371/journal.pcbi.1006650.

22 Sawai, H.; Kim, H.L.; Kuno, K.; Suzuki, S.; Gotoh, H.; Takada, M.; Takahata, N.; Satta, Y.; Akishinonomiya, F. The origin and genetic variation of domestic chickens with special reference to junglefowls *Gallus g. gallus* and *G. varius. PloS One* **2010**, *5*, e10639. doi:10.1371/journal.pone.0010639.

23 Hata, A.; Nunome, M; Suwanasopee, T.; Duengkae, P.; Chaiwatana, S.; Chamchumroon, W.; Suzuki, T.; Koonawootrittriron, S.; Matsuda, Y.; Srikulnath, K. Origin and evolutionary history of domestic chickens inferred from a large population study of Thai red junglefowl and indigenous chickens. *Sci. Rep*. **2021**, *11*, 1–15. doi:10.1038/s41598-021-81589-7.

24 Singchat, W.; Chaiyes, A.; Wongloet, W.; Ariyaraphong, N.; Jaisamut, K.; Panthum, T.;

Ahmad, S.F.; Chaleekarn, W.; Suksavate, W.; Inpota, M.; et al. Red junglefowl resource management guide bioresource reintroduction for sustainable food security in Thailand. *Sustainability* **2022**, *14*, 7895. doi:10.3390/su14137895.

25 Guo, S.W.; Thompson, E.A. Performing the exact test of Hardy-Weinberg proportion for multiple alleles. *Biometrics* **1992**, *48*, 361–372. doi:10.2307/2532296.

26 Raymond, M.; Rousset, F. An exact test for population differentiation. *Evolution* **1995**, *49*, 1280–1283. doi:10.1111/j.1558-5646.1995.tb04456.x.

27 R Core Team. *R: A Language and Environment for Statistical Computing; R Foundation for Statistical Computing*. Austria: Vienna, 2022.

28 Welch, B. L. The generalization of student's' problem when several different population variances are involved. *Biometrika* **1947**, *34*, 28–35. doi:10.2307/2332510.

29 Goudet, J.F. FSTAT (version 1.2): a computer program to calculate F-statistics. *J. Hered.* **1995**, *86*, 485–486. doi:10.1093/oxfordjournals.jhered.a111627.

30 Van, O.C.; Hutchinson, W.F.; Wills, D.P.; Shipley, P. MICRO-CHECKER: software for identifying and correcting genotyping errors in microsatellite data. *Mol. Ecol. Notes* **2004**, *4*, 535–538. doi:10.1111/ j.1471-8286.2004.00684.x.

31 Park, S.D.E. *The Excel Microsatellite Toolkit (version 3.1)*. Ireland: Animal Genomics Laboratory, University College, 2001.

32 Peakall, R.; Smouse, P.E. GenAlEx 6.5: genetic analysis in Excel. Population genetic software for teaching and research–an update. *J. Bioinform.* **2012**, *28*, 2537–2539. doi:10.1093/bioinformatics/bts460.

33 Lynch, M.; Ritland, K. Estimation of pairwise relatedness with molecular markers. *J. Genet.* **1999**, *152*, 1753–1766. doi:10.1093/genetics/152.4.

34 Wang, J. COANCESTRY: a program for simulating, estimating and analysing relatedness and inbreeding coefficients. *Mol. Ecol. Resour.* **2011**, *11*, 141–145. doi:10.1111/j.1755-0998.2010.

35 Præstgaard, J.T. Permutation and bootstrap Kolmogorov-Smirnov tests for the equality of two distributions. *Scand. J. Stat.* **1995**, 22, 305–322.

36 Chapuis, M.P.; Estoup, A. Microsatellite null alleles and estimation of population differentiation. *Mol. Biol. Evol.* **2007**, *24*, 621–631. doi:10.1093/molbev/msl191.

37 Nei, M. Genetic distance between populations. *Am. Nat.* **1972**, *106*, 283–292. doi:10.1086/ 282771.

38 Piry, S.; Luikart, G.; Cornuet, J.M. BOTTLENECK: a computer program for detecting recent reductions in the effective population size using allele frequency data. *J. Hered.* **1999**, *90*, 502–503. doi:10.1093/jhered/90.4.502.

39 Garza, J.C.; Williamson, E.G. Detection of reduction in population size using data from microsatellite loci. *Mol. Ecol.* **2001**, *10*, 305–318. doi:10.1046/j.1365-294X.2001.01190.x.

40 Jombart, T. adegenet: a R package for the multivariate analysis of genetic markers. *J. Bioinform.* **2008**, *24*, 1403–1405. doi:10.1093/bioinformatics/btn129.

41 Pritchard, J.K.; Stephens, M.; Donnelly, P. Inference of population structure using multilocus genotype data. *J. Genet.* **2000**, *155*, 945–959. doi:10.1093/genetics/155.2.945.

42 Earl, D.A. Structure harvester: a website and program for visualizing STRUCTURE output and implementing the Evanno method. *Conserv. Genet. Resour.* **2012**, *4*, 359–361. doi:10.1007/s12686-011-9548-7.

43 Linnaeus, C. *Systema Naturæ per Regna tria Naturæ, Secundum Classes, Ordines, Genera, Species, cum Characteribus, Differentiis, Synonymis, Locis*. Austria: Tomus, I., Ed.; Reformata: Holmiæ, Salvius; Reformata: Holmiæ, Salvius; Vindobonae: Vienn, 1758

44 Do, C.; Waples, R.S.; Peel, D.; Macbeth, G.M.; Tillett, B.J.; Ovenden, J.R. NeEstimator v2: Reimplementation of software for the estimation of contemporary effective population size (N_e) from genetic data. *Mol. Ecol. Resour.* **2014**, *14*, 209–214. doi: 10.1111/1755-0998.12157
