# Supplementary figures and images for: Environmental and Socio–Cultural Factors Impacting the Unique Gene Pool Pattern of Mae Hong-Son Chicken
Source: Animals (Basel). 2023 Jun 10;13(12):1949. doi: 10.3390/ani13121949 (PMC10295432; doi:10.3390/ani13121949)

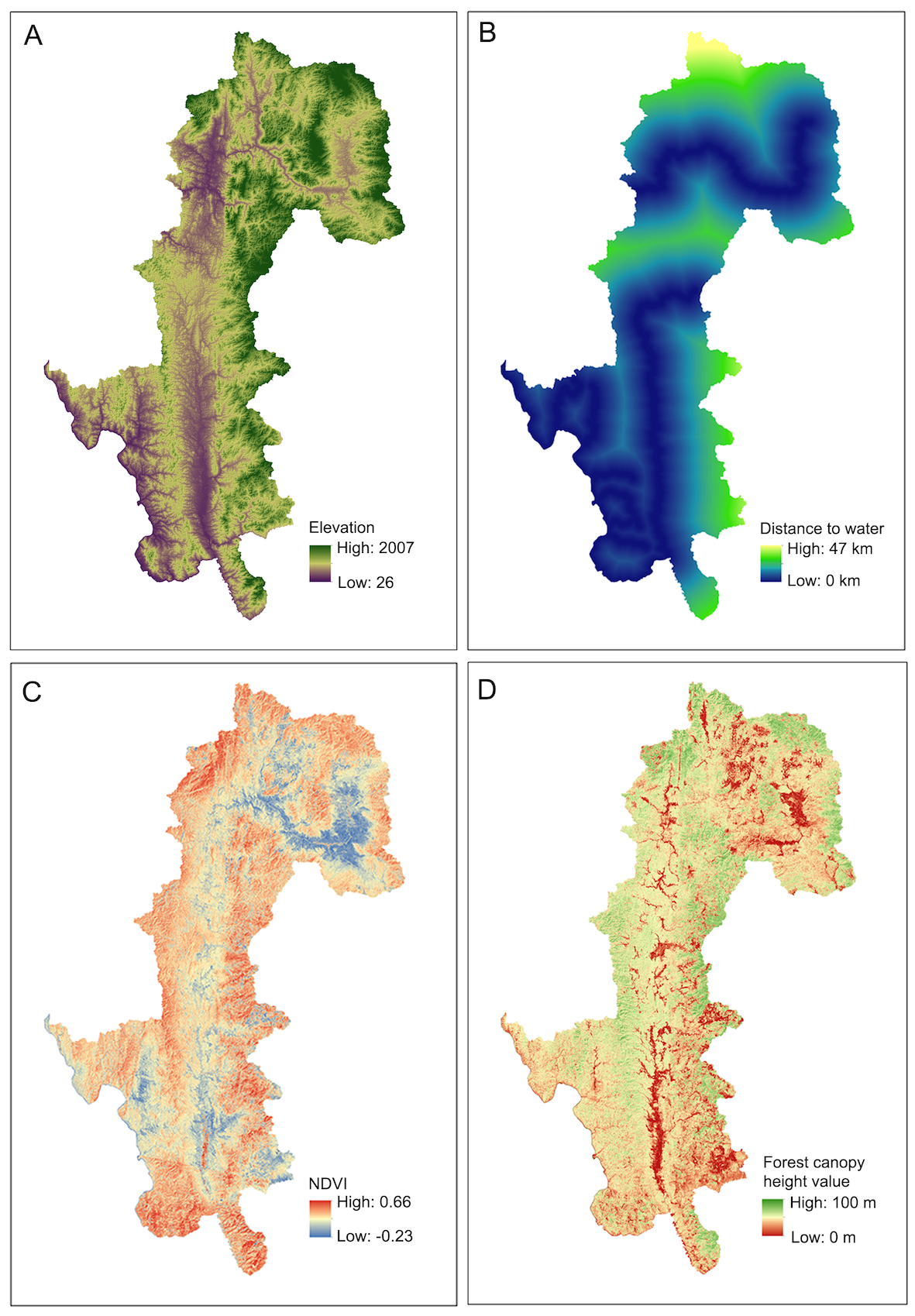

Supplement: Supplementary file 1 [file animals-13-01949-s001.zip › Figure S1.tif]

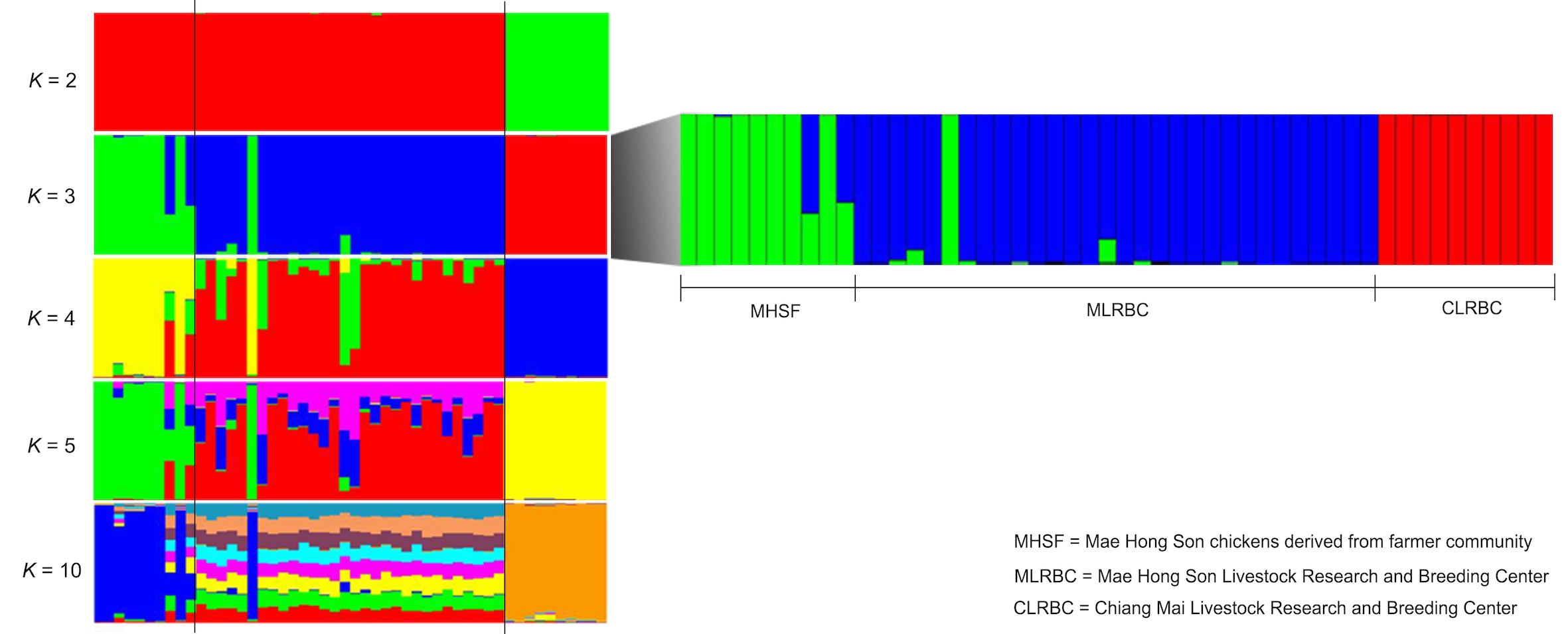

Supplement: Supplementary file 1 [file animals-13-01949-s001.zip › Figure S10.tif]

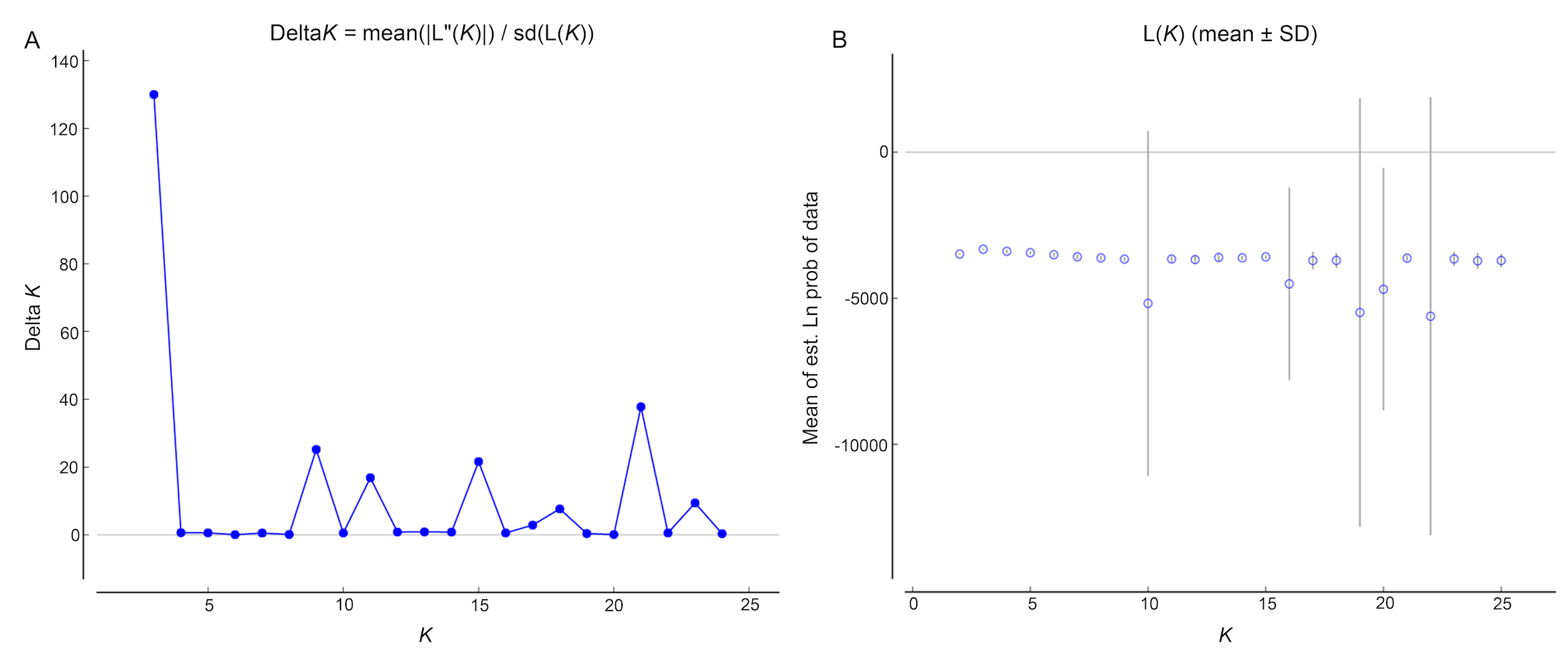

Supplement: Supplementary file 1 [file animals-13-01949-s001.zip › Figure S11.tif]

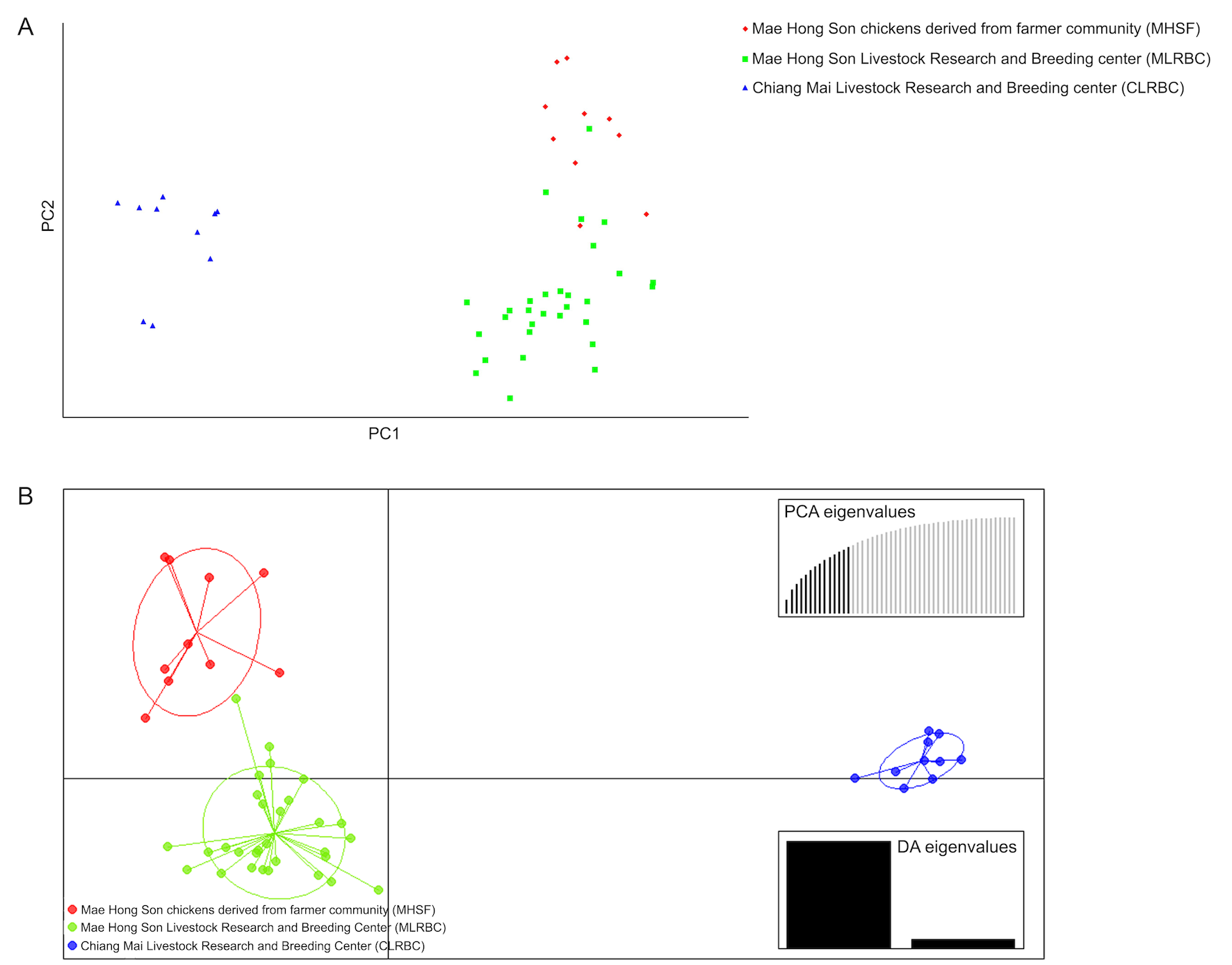

Supplement: Supplementary file 1 [file animals-13-01949-s001.zip › Figure S12.tif]

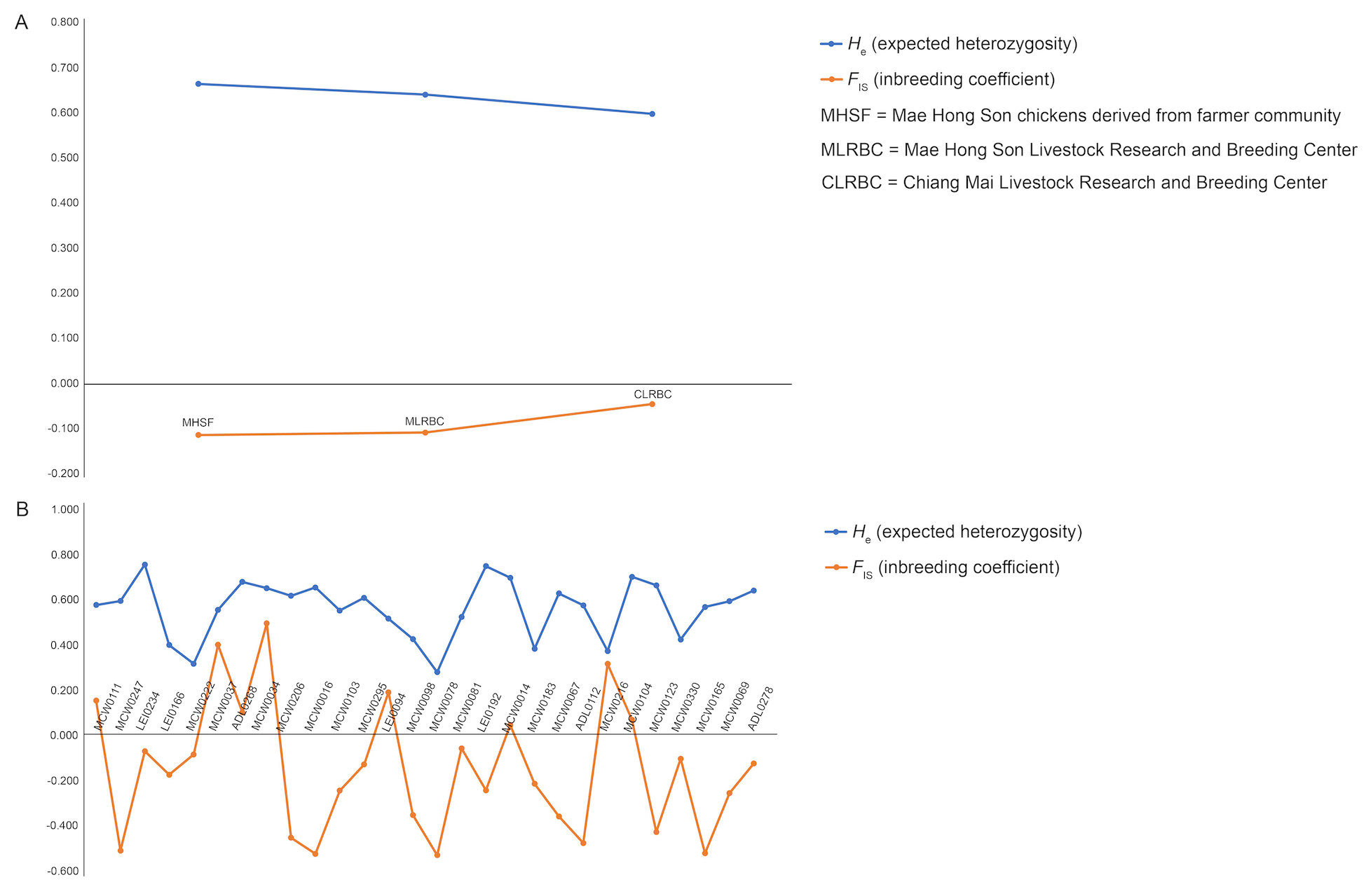

Supplement: Supplementary file 1 [file animals-13-01949-s001.zip › Figure S13.tif]

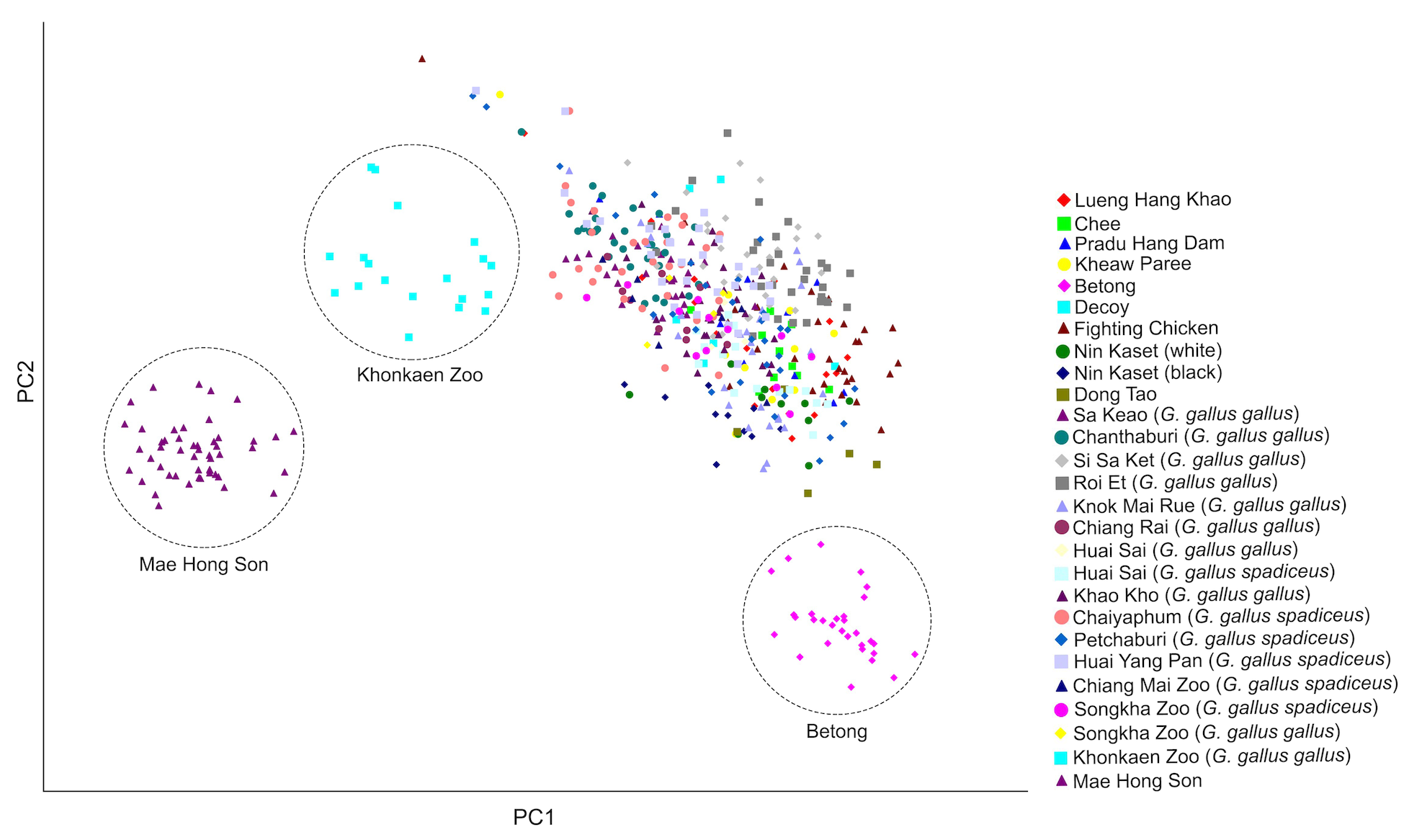

Supplement: Supplementary file 1 [file animals-13-01949-s001.zip › Figure S14.tif]

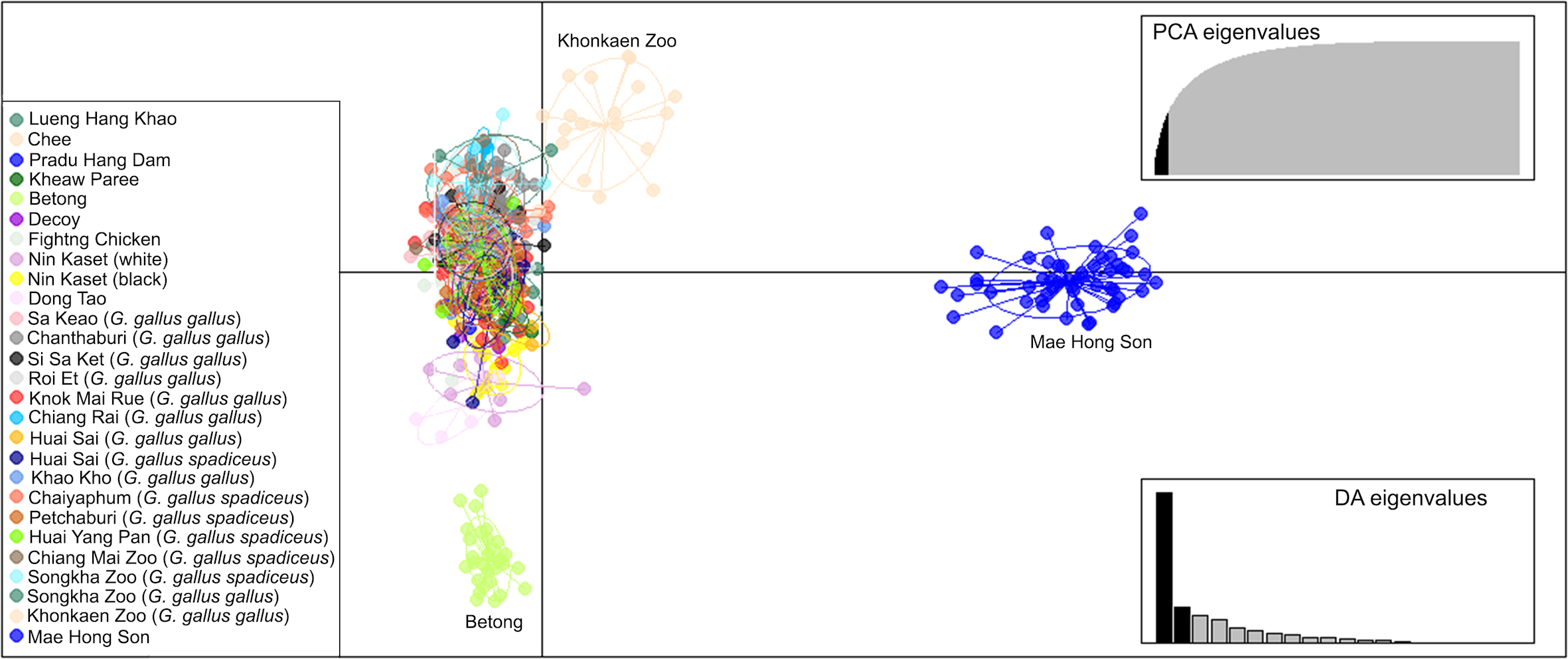

Supplement: Supplementary file 1 [file animals-13-01949-s001.zip › Figure S15.tif]

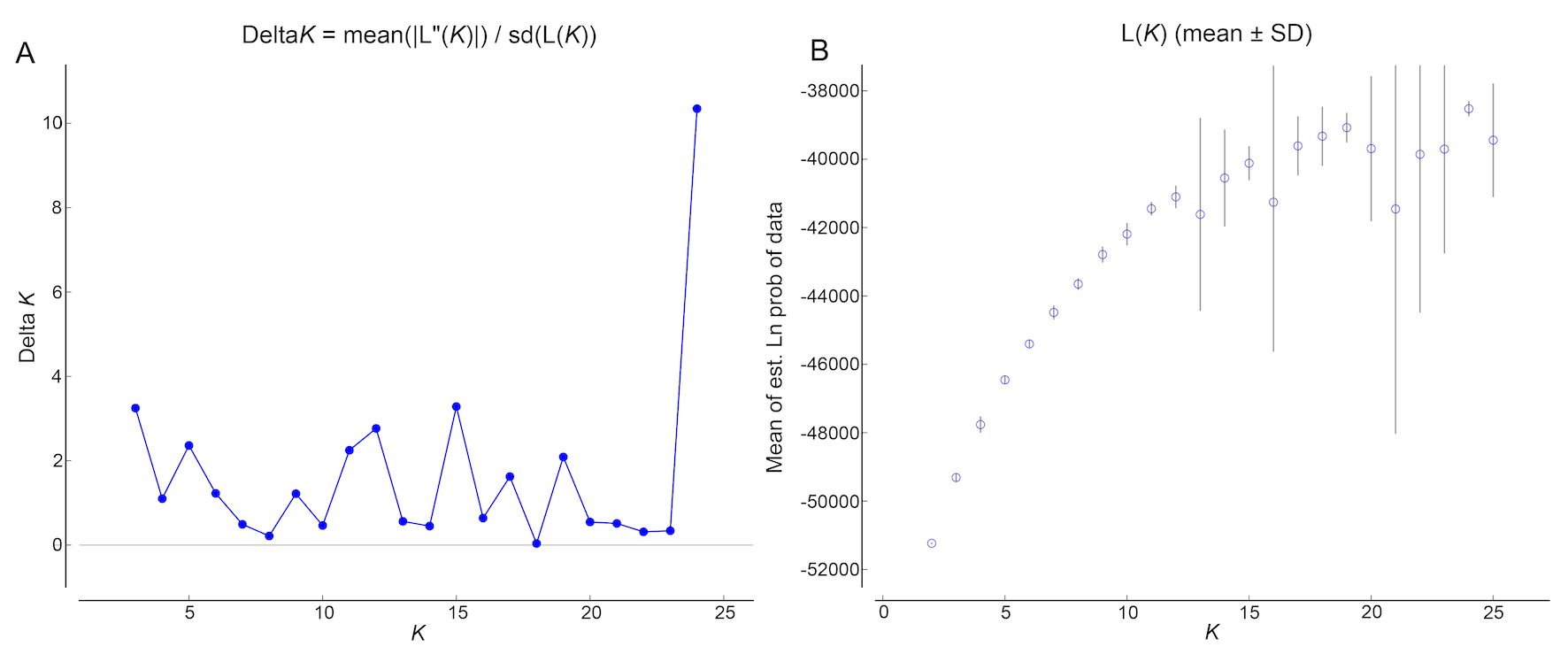

Supplement: Supplementary file 1 [file animals-13-01949-s001.zip › Figure S16.tif]

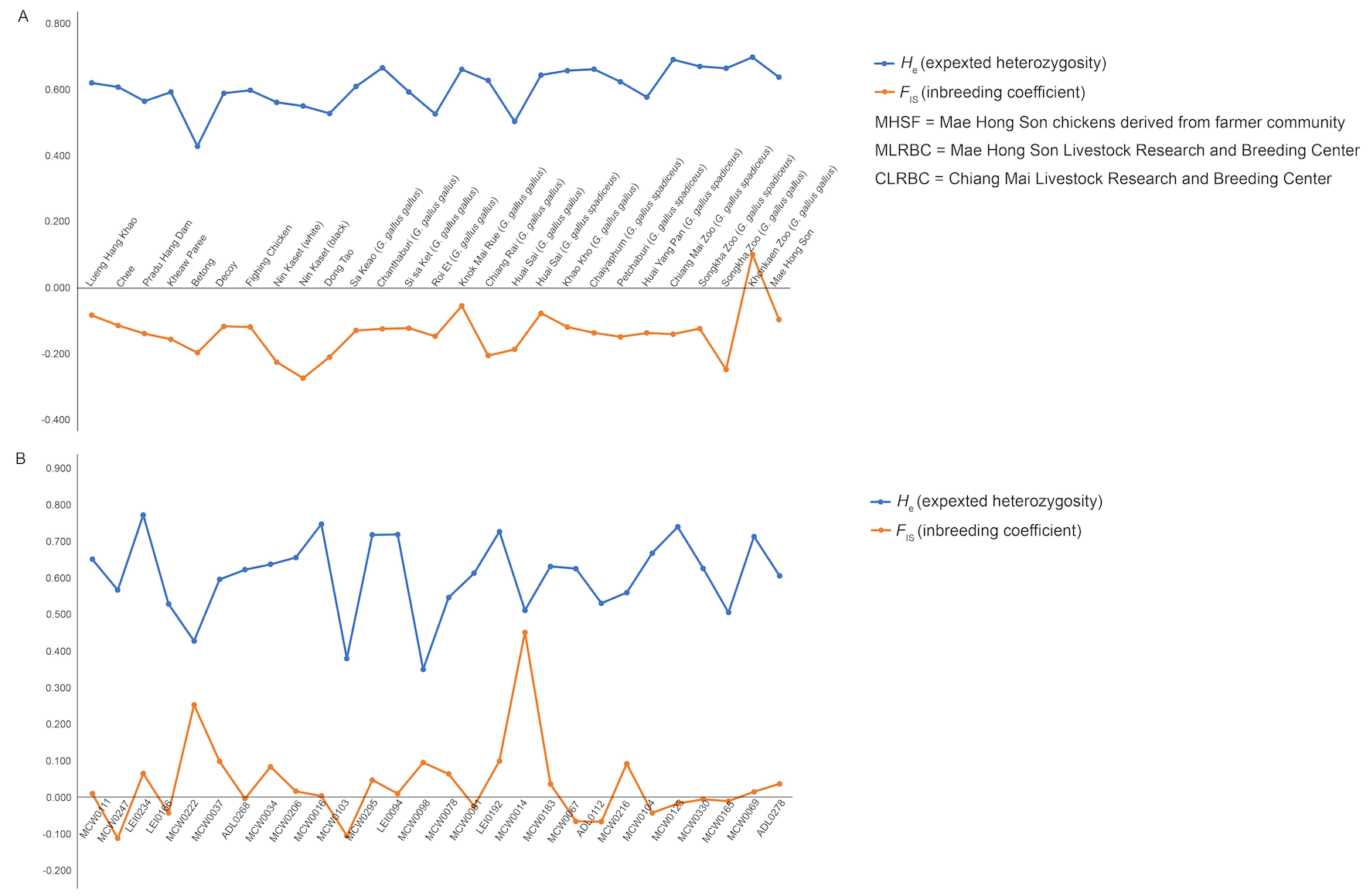

Supplement: Supplementary file 1 [file animals-13-01949-s001.zip › Figure S17.tif]

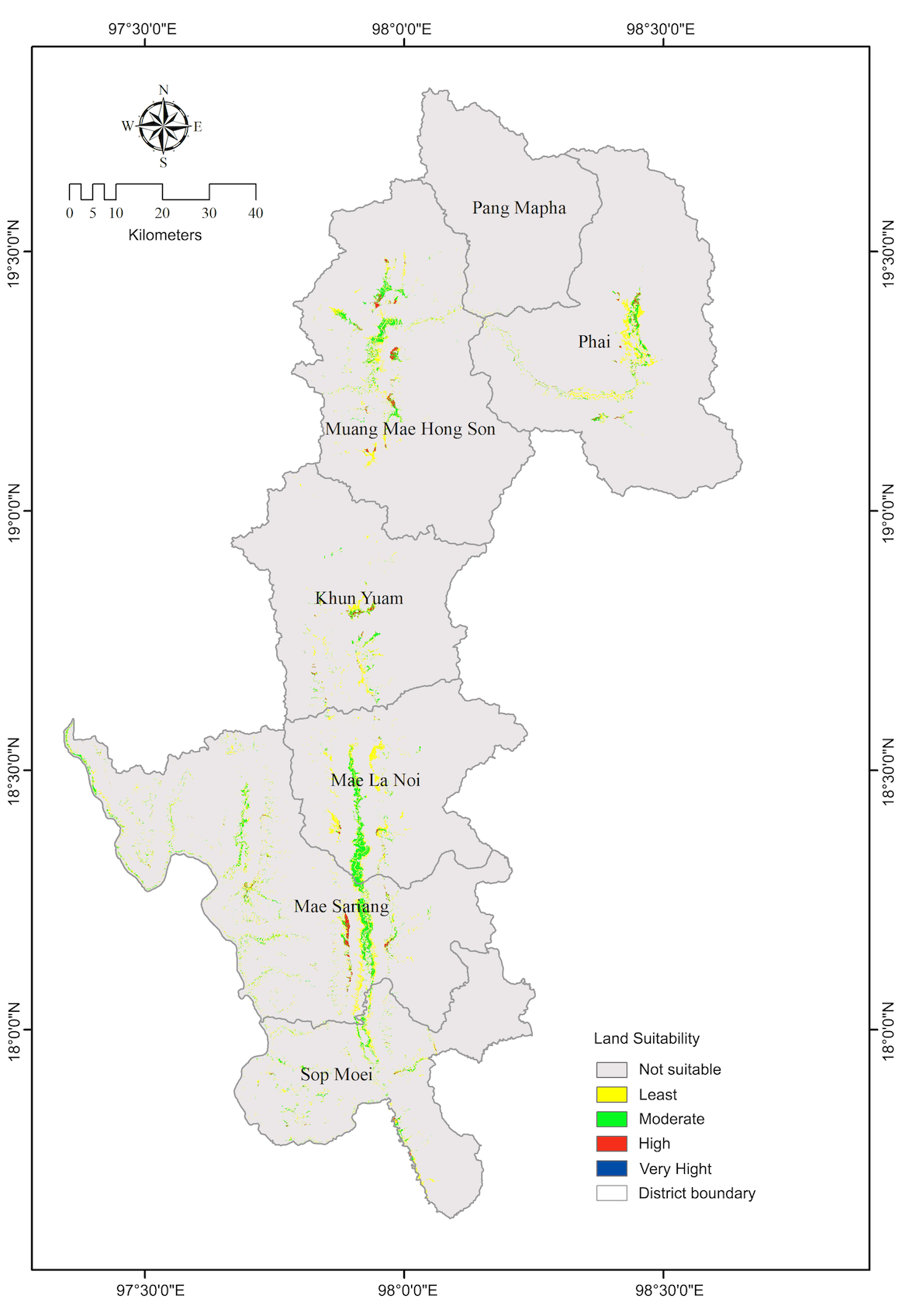

Supplement: Supplementary file 1 [file animals-13-01949-s001.zip › Figure S3.tif]

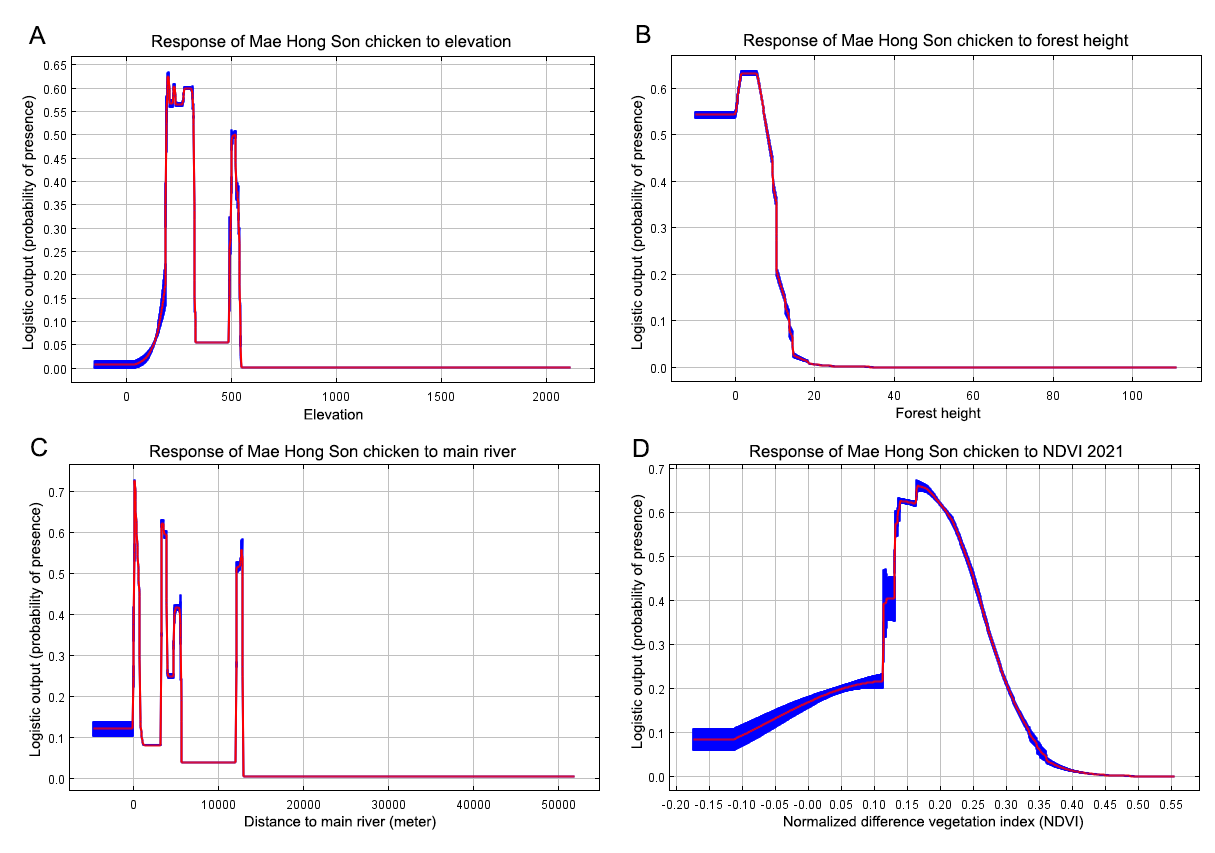

Supplement: Supplementary file 1 [file animals-13-01949-s001.zip › Figure S4_RV1.tiff]

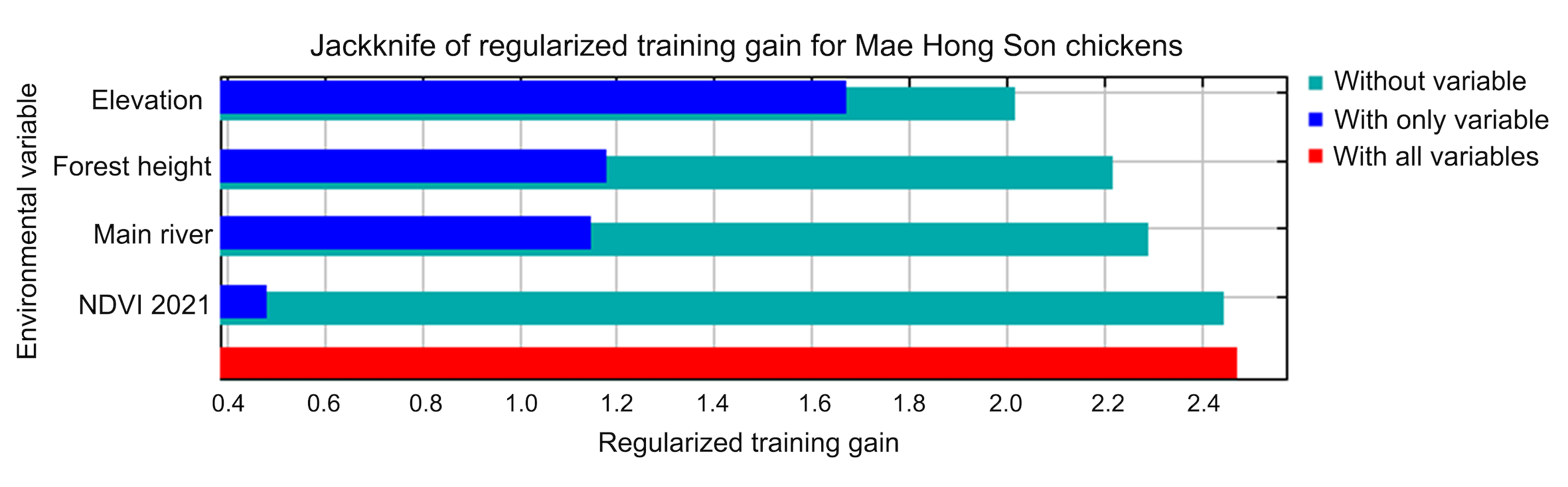

Supplement: Supplementary file 1 [file animals-13-01949-s001.zip › Figure S5.tif]

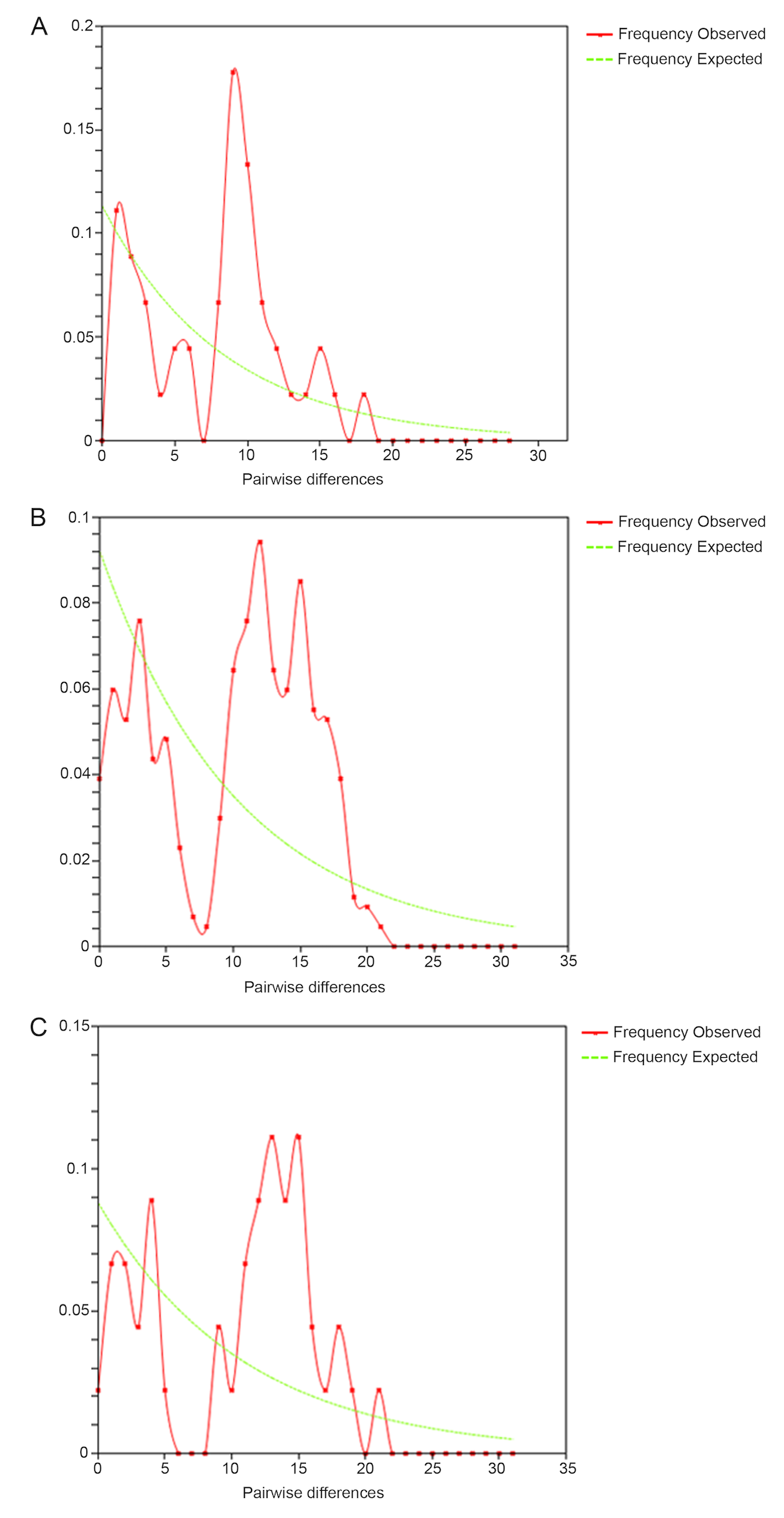

Supplement: Supplementary file 1 [file animals-13-01949-s001.zip › Figure S6.tif]

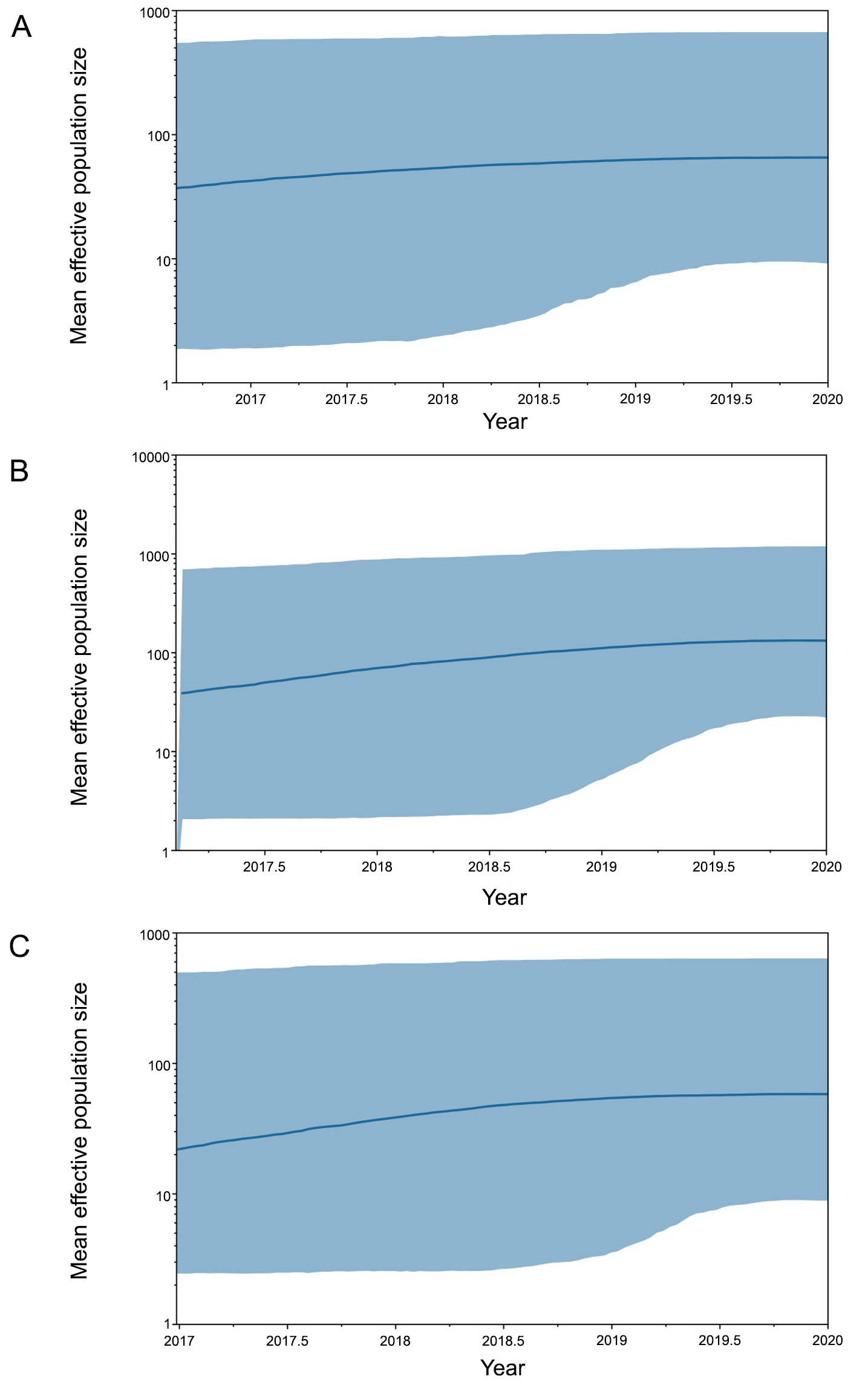

Supplement: Supplementary file 1 [file animals-13-01949-s001.zip › Figure S7.tif]

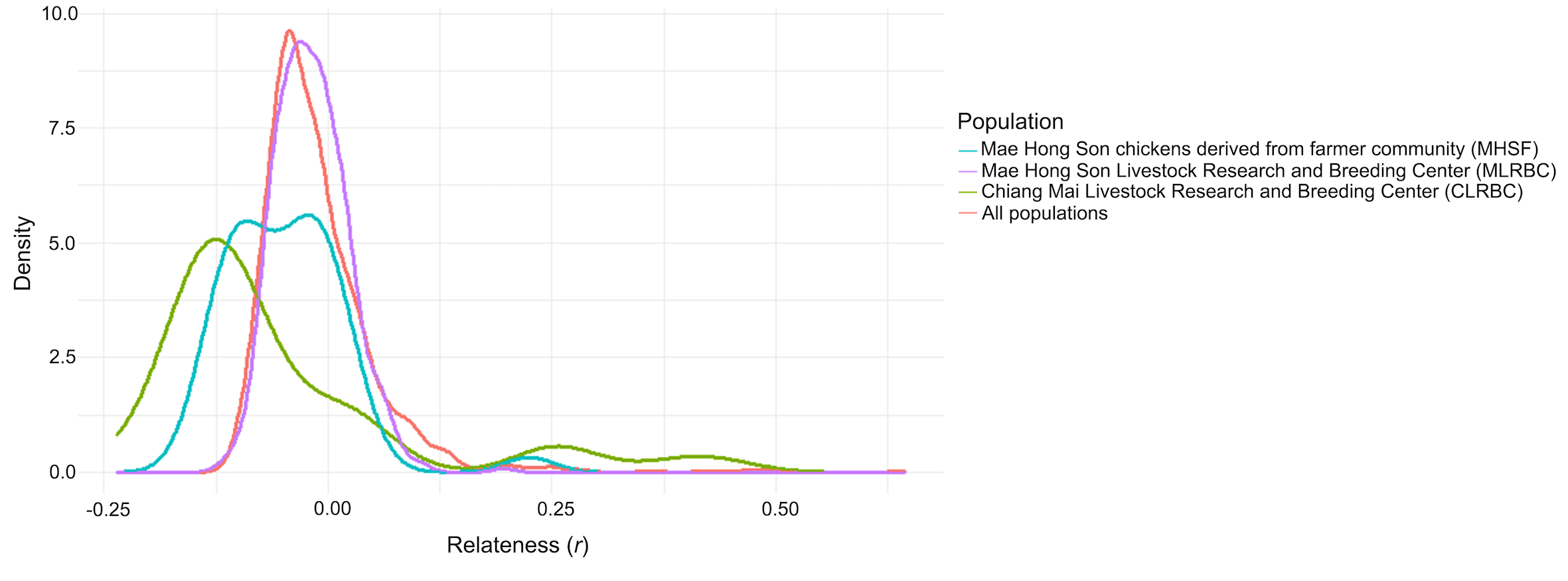

Supplement: Supplementary file 1 [file animals-13-01949-s001.zip › Figure S8.tif]

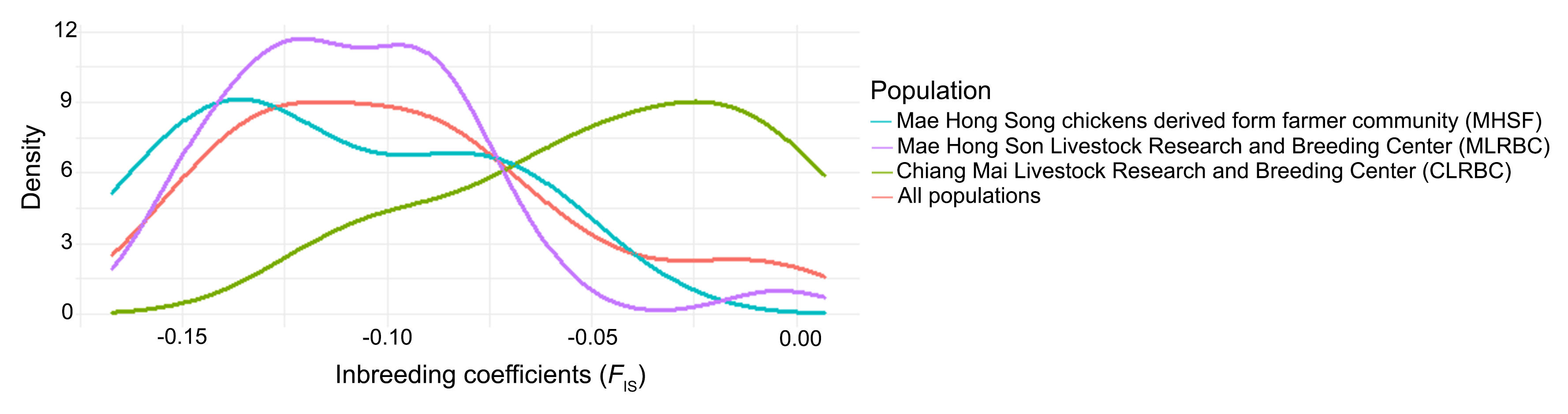

Supplement: Supplementary file 1 [file animals-13-01949-s001.zip › Figure S9.tif]
